# Differences in virus and immune dynamics for SARS-CoV-2 Delta and Omicron infections by age and vaccination histories

**DOI:** 10.1186/s12879-024-09572-x

**Published:** 2024-06-29

**Authors:** Maxine W Tan, Anet J.N. Anelone, An Ting Tay, Ren Ying Tan, Kangwei Zeng, Kelvin Bryan Tan, Hannah Eleanor Clapham

**Affiliations:** 1 Saw Swee Hock School of Public Health, National University of Singapore, Singapore, Singapore; 2 Ministry of Health, Singapore, Singapore; 3 National Centre for Infectious Diseases, Singapore, Singapore; 4 Duke-NUS Graduate Medical School, National University of Singapore, Singapore, Singapore

**Keywords:** SARS-Cov-2, Variants, Modelling, Bayesian inference, Within-host, Mechanistic model, Age, Immunity, Vaccination, Waning

## Abstract

Vaccination against COVID-19 was integral to controlling the pandemic that persisted with the continuous emergence of SARS-CoV-2 variants. Using a mathematical model describing SARS-CoV-2 within-host infection dynamics, we estimate differences in virus and immunity due to factors of infecting variant, age, and vaccination history (vaccination brand, number of doses and time since vaccination). We fit our model in a Bayesian framework to upper respiratory tract viral load measurements obtained from cases of Delta and Omicron infections in Singapore, of whom the majority only had one nasopharyngeal swab measurement. With this dataset, we are able to recreate similar trends in URT virus dynamics observed in past within-host modelling studies fitted to longitudinal patient data.

We found that Omicron had higher R_0,within_ values than Delta, indicating greater initial cell-to-cell spread of infection within the host. Moreover, heterogeneities in infection dynamics across patient subgroups could be recreated by fitting immunity-related parameters as vaccination history-specific, with or without age modification. Our model results are consistent with the notion of immunosenescence in SARS-CoV-2 infection in elderly individuals, and the issue of waning immunity with increased time since last vaccination. Lastly, vaccination was not found to subdue virus dynamics in Omicron infections as well as it had for Delta infections.

This study provides insight into the influence of vaccine-elicited immunity on SARS-CoV-2 within-host dynamics, and the interplay between age and vaccination history. Furthermore, it demonstrates the need to disentangle host factors and changes in pathogen to discern factors influencing virus dynamics. Finally, this work demonstrates a way forward in the study of within-host virus dynamics, by use of viral load datasets including a large number of patients without repeated measurements.

**Supplementary Information:**

The online version contains supplementary material available at 10.1186/s12879-024-09572-x.

## Introduction

The persistence of the COVID-19 pandemic can be largely attributed to the continuous evolution of the virus, resulting in the emergence of new virus variants over time. New variants may have differing properties such as their ability to infect and transmit, associated disease severity, and susceptibility to pre-existing immunity. Both Delta (B.1.617) and Omicron (B.1.1.529) have shown greater transmissibility and immune escape capacity than past variants of concern (VOC) [1, 2]. Vaccinated and previously infected individuals showed decreased neutralising capacity of sera against Delta infection compared to pre-Delta VOCs [3, 4]. Meanwhile, vaccinated and previously infected individuals demonstrated reduced levels of protection against re-infection and symptomatic disease for Omicron compared to previously circulating VOCs, although protection against severe disease during infection was maintained [5–7].

The effectiveness of the developed COVID-19 vaccines is challenged with each new variant. A systematic review reported that two doses of vaccine induced lower neutralisation titres against Omicron compared to Wild Type virus, highlighting the need for booster doses to increase antibody levels [8]. Moreover, individuals who received non-mRNA vaccines had poorer neutralising ability against Delta and Omicron VOCs and faster waning antibody and cellular immune responses compared to those with mRNA vaccines [9]. However, it must be noted that the global immunological landscape against SARS-CoV-2 has transformed in parallel with the pandemic. This is a result of natural infection and mass vaccination programmes that have elevated population immunity levels [10]. Hence, it is challenging to discern whether observations of new variants such as increased ability to evade prior immunity are a result of virus evolution, or increased population immunity.

**Fig. 1:**
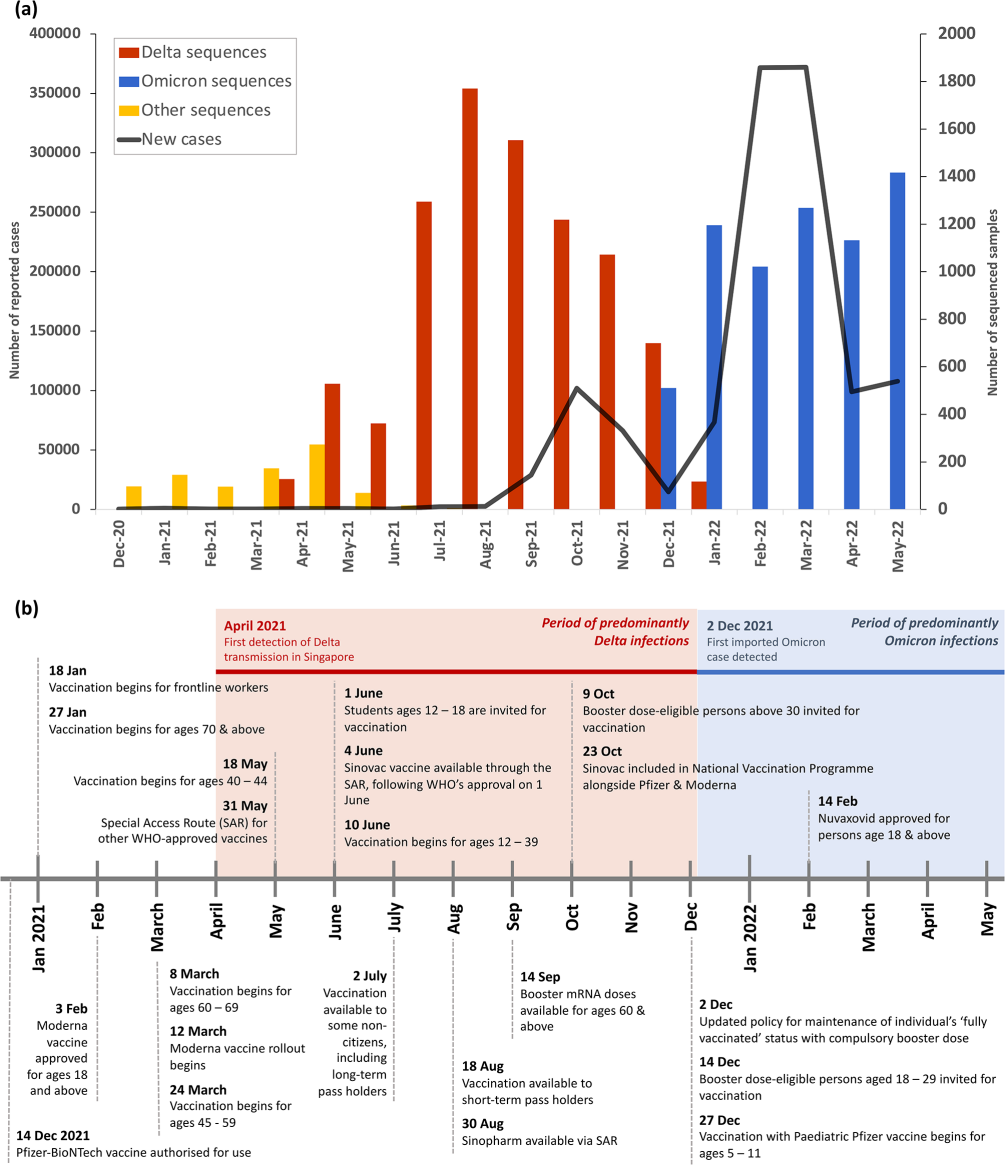
Timeline of Singapore’s COVID-19 pandemic and vaccination programme from December 2020 to May 2022. (**a**) The number of monthly reported COVID-19 cases is represented by the black line and follows the left axis. The number of genomic sequences for Delta, Omicron and other variants are represented by the red, blue, and yellow bars respectively, and follow the right axis. (**b**) Timeline of Singapore’s mass vaccination programme for COVID-19, including details of approvals for vaccine usage locally. The red block represents the approximate time period in which Delta was the predominantly circulating variant, April 2021 to early December 2021. The blue block represents the approximate time period in which Omicron was the predominantly circulating variant, early December 2021 onwards

Singapore launched its National Vaccination Programme against COVID-19 on 30 December 2020, with a key strategy of prioritising higher risk sub-populations during the gradual vaccine rollout, such as elderly and healthcare workers (Fig. 1b). Older age groups were identified to be of higher risk, with increased susceptibility to infection and propensity for onset of severe symptoms given infection. This is not only due to their comorbidities but possibly also due to the decay of immune function with age, known as immunosenescence [11]. Due to the time period of vaccination being determined by an individual’s age, waning immunity results in inevitable differences in immunity across age groups at any given point in time [12]. While vaccination was being made available to older age groups, Singapore saw an increasing number of cases due to the importation and local transmission of Delta VOC from April 2021 [13]. This resulted in a wave of infections due to Delta, as it replaced the previously circulating VOCs of Alpha, Beta and Gamma and became the predominantly circulating variant up until December 2021 with the introduction of Omicron (Fig. 1a). By January 2022, Omicron replaced Delta as the predominantly circulating variant [14] and Singapore faced a corresponding surge in COVID-19 cases while it commenced its booster dose administration.

Previous within-host models of SARS-CoV-2 infection have been developed to study the relationship between viraemia and disease parameters [15, 16], interactions between pathogen and host immune response [17–19], and treatments [20, 21]. Most of these within-host models have been focussed on the characterisation of disease and virus parameters, such as infectiousness [15, 22], and virus latent period [22], with recent work studying the effects of vaccination on antibody dynamics [23]. These models were fitted to a small number of patients with intensive follow up, often obtaining best-fit individual parameter values.

Here, we utilise a large dataset consisting of upper respiratory tract (URT) viral load measurements from 70,355 patients of whom the majority only had one nasopharyngeal swab measurement. By adopting a Bayesian approach, we estimate and analyse the best-fitting parameters to characterise Delta and Omicron infection dynamics of different patient subgroups categorised by age, vaccine brand and doses received, and time since last vaccine dose. Using a mathematical model describing SARS-CoV-2 URT infection with a simple clearing host immune response, we sought to gain insight into the role of infection rate of target cells, natural virus clearance, growth rate of immunity during infection, and infected cell clearance by immune response in influencing observed inter-group variability in infection and immune dynamics across VOCs, and patient subgroups.

## Materials and methods

### Data and the subsequent subsets used

The data was obtained from participating General Practitioner clinics that were registered into the local government’s Public Health Preparedness Clinics (PHPC) scheme and enrolled into the national Swab and Send Home (SASH) programme. This dataset includes 159,178 members of the community who attended Swab and Send Home clinics in Singapore, with PCR swabs collected from 25 April 2021 to 23 May 2022 (S1 Fig). Majority of the individuals (87.2%) only had one swab taken during their infection, as per the country’s swab protocol during the period of data collection. Individuals with more than one swab recorded (12.8%) had consequent recovery swabs taken while they were in recovery. These individuals were more closely monitored as they were identified to be at higher risk of severe disease, such as elderly, unvaccinated, or vaccinated with non-mRNA-based vaccines against COVID-19.

For our analysis, we only included the 70,355 individuals who were unvaccinated or received minimally two doses of a COVID-19 vaccine either made available through the national vaccination programme or that was commonly administered in Singapore. This includes Pfizer-BioNTech/Comirnaty, Moderna, Sinovac-CoronaVac and Sinopharm vaccines. We sought to study the effects of different vaccination histories and age on within-host dynamics of virus and immunity. Hence, patients were categorised into vaccine groupings based on the vaccine brand(s) and number of doses of COVID-19 vaccine received. Moreover, we grouped patients by age, according to the age stratification used in Singapore’s COVID-19 vaccination policies. Patients were categorised into one of six age groups: (1) 0–4 years, (2) 5–11 years, (3) 12–17 years, (4) 18–39 years, (5) 40–60 years, and (6) above 60 years. Lastly, to consider the effects of waning immunity, patients were grouped according to the time since their last received dose of COVID-19 vaccine; with the first group being those who are infected within 1 week of receiving their last dose of vaccine (0–7 days), then those within the 2nd week (8–14 days), within the 3rd to 4th week (15–28 days), within the 2nd month (29–60 days), within the 3rd month (61–90 days), followed by grouping by 90-day intervals (91–180 days, 181–270 days, 271–360 days).

Since no information on identity of infecting variant was provided, we inferred separation of Delta and Omicron B.1.1.529 waves using sequencing data made available by GISAID [14] (Fig. 1). Patients with date of symptom onset between 1 June 2021 to 30 November 2021 were categorised as Delta infections, and those between 1 January 2022 to 18 February 2022 as Omicron infections. We were unable to differentiate between infections with different B.1.1.529 sublineages in our dataset. However, the Omicron wave in Singapore was dominated by BA.1 infections till 4 February 2022, followed by co-dominance of BA.1 and BA.2, then with BA.2 being the predominantly circulating sublineage from 17 February 2022 [24]. As we defined Omicron infections in our dataset as infections with date of symptom onset between 1 January 2022 to 18 February 2022, the majority of our dataset should include BA.1 infections since the total nasopharyngeal swabs taken each day markedly decreased from mid-February (S1 Fig).

URT viral load data was recorded as cycle threshold (Ct) values, we converted this to viral RNA copy number values (copies/mL) using the formula ${log}_{10}\left(viral RNA copies,\frac{copies}{mL}\right)=-0.32\left(Ct\right)+14.11$ as in Kim et al. [25]. Each individual’s date of symptom onset and date of swab(s) taken were recorded. However, as we model within-host dynamics from the start of infection, we had to account for the incubation period of virus before symptom onset. We sampled each patient’s incubation period based on whether they were classified to have Delta or Omicron infection, assuming normal distributions with means of 4.41 and 3.42 days and standard deviations of 0.32 and 0.27 days respectively, based on the reported mean and 95% confidence interval from a meta-analysis of incubation periods for SARS-CoV-2 variants [26].

### Within-host model

Since only viral load data was available for model fitting, we use a simple compartmental deterministic model describing SARS-CoV-2 infection within an individual, detailing virus and immune dynamics. The model describes uninfected target cells (T), infected target cells (I), free virus (V), and a generic clearing immune response (Z). The model is detailed mathematically as:

$$\frac{dT}{dt}=A-\alpha T-\beta VT$$

$$\frac{dI}{dt}=\beta VT-\delta I-\gamma IZ$$

$$\frac{dV}{dt}=pI-cV$$

$$\frac{dZ}{dt}=\omega IZ$$

In the model, uninfected target cells are assumed to be produced at constant rate A and die at rate α per cell. The target cells are infected by virus in a mass action manner, resulting in productively infected cells, I. Infected cells die at rate δ and are cleared by the immune response via a mass action process. Free virus is produced at rate p per infected cell, and cleared at rate c. Although it is also possible for free virus to also be cleared by the immune response, we assume that infected cell clearance is the main mechanism of the immune response to inhibit infection. Immune response, Z, is assumed to grow in a mass action manner at rate ω, in response to infected cells. We recognise that the adaptive immune response in our model is an over-simplification of a complex system, however in the absence of lymphocyte and antibody data, incorporating a more complex and biologically representative form would result in issues of non-identifiability.

The within-host basic reproduction number, R_0,within_, is the expected number of susceptible target cells infected by a single infected cell. For our model, R_0,within_ is calculated as R_0,within_ = βpT_0_/[c(δ + γZ_0_)].

### Parameter estimation

The model consists of 8 parameters, 4 of which are fixed (Table 1). We assign values to these fixed parameters as not all parameters are independent given the lack of data of other compartments of the system. We estimate the infection rate of target cells (β), natural clearance of virus (c), infected cell clearance by immune response (γ), and growth rate of immunity (ω).

**Table 1:** Parameters of the within-host model. Values at which fixed parameters are held constant, and priors for estimated parameters are stated

Parameter	Parameter description	Assigned/Estimated	Value/Prior
A	Target cell production per mL and per day	Assigned	1.12 × 10^7^ [27, 28]
α	Target cell death rate per day	Assigned	0.14 [28]
β	Infection rate of target cells per virion	Estimated	[10^− 12^: 10^− 9^]
δ	Natural clearance of infected cells per day	Assigned	0.14 [28]
γ	Immune response clearance of infected cells per immune cell per day	Estimated	[0:10]
*p*	Virus production rate per infected cell per day	Assigned	1.12 × 10^4^ [29]
c	Natural clearance of virus per day	Estimated	[0.01:10]
ω	Growth rate of immunity per infected cell per day	Estimated	[0:1]
T_0_	Initial population size of uninfected target cells per mL	Assigned	8 × 10^7^
V_0_	Initial virus inoculum per mL	Assigned	1
Z_0_	Initial immune effector population per mL	Assigned	10 (arbitrary, scales with γ)
θ	Age modifier vector for parameters estimated at age modified vaccination history-specific level	Estimated	[0:inf]

Since a large majority of patients only had one nasopharyngeal swab taken, we were unable to estimate parameters at an individual level. Instead, we adopted a Bayesian approach and fit the model to individual patient’s data, with parameters estimated at a group level. The fitted parameters are estimated at one of three levels. The first level is variant-specific, such that all individuals with the same infecting VOC will have the same parameter estimate. The second level is vaccination history-specific, where there is one parameter estimate for all individuals from the same vaccine group who received their latest dose of vaccine within the same time period, estimated independently of other time periods. The third level is age modified vaccination history-specific, where there is one parameter estimate for individuals in the same age group, who have received the same vaccines and their latest vaccine dose within the same time period. To assess which parameters best explain differences in viral dynamics between vaccine histories and age groups, we consider four ODE within-host models that include different combinations of parameters fitted at different levels (S2 Table).

Depending on the model type, certain immunity-related parameters are estimated as age-modified vaccination history-specific. In these models, either none of the immunity-related parameters are age-modified, only growth rate of immunity (ω) is age-modified, only immune clearance of infected cells (γ) is age-modified, or both immunity-related parameters are age-modified. To fit a parameter as age-modified vaccination history-specific, we included an age modifier parameter, θ, that acted as a multiplier which scales the selected immunity-related parameter for each age group. We assign the 18–39 years age group as our reference group, with θ_reference_ = θ_18−39 yrs_ = 1.0. A multiplier value was estimated for each of the remaining age groups and scales their corresponding immunity-related parameter value, relative to the 18–39 years age group.

The within-host models are fitted to measurements of URT viral load using RStan, a programme interfacing R and Stan, allowing for statistical inference by use of Markov chain Monte Carlo (MCMC) [30]. We assumed log viremia measurements to have normally distributed errors, with σ^2^ taken to be 1. Below, φ is the probability density function (pdf) of the normal distribution, D_i_ are the URT viral load measurements, X_i_ are the model predictions, and n is the total number of measurements in the dataset.

$$\prod _{i=1}^{n}\varphi \left(\text{log}\left({D}_{i}\right)\right|\text{log}\left({X}_{i}\right), {\sigma }^{2})$$

Akaike information criterion, corrected Akaike information criterion, Bayesian information criterion, and median log-likelihood are used to compare model performance.

### Model selection procedure

We considered multiple ordinary differential equation (ODE) within-host models, henceforth referred to as model types, in which parameters are varied at different levels to determine which model type is able to recreate trends in the data and assess which parameters best explain differences in viral dynamics between subgroups. We explored four model types in which the immunity-related parameters, immune clearance of infected cells (γ) and growth rate of immunity (ω), were fitted at one of two levels: vaccination history-specific, or age-modified vaccination history-specific (S2 Table). The two virus-related parameters, infection rate of target cells (β) and natural clearance rate of free virus (c) were assumed to be intrinsic to the SARS-CoV-2 variant, and were hence varied by VOC across all four model types.

### Sensitivity analyses

We conducted sensitivity analyses to assess the impact of our assumptions and potential biases on model fit results. To assess model robustness to incubation period, we repeated the above model fitting with the selected model type 2 using a dataset in which we swapped the incubation period distributions used for Delta and Omicron infections. To evaluate the impact of longitudinal sampling on virus dynamics, we fitted the model type 2 to two datasets. The first dataset only includes the first recorded swab of each individual while the second only includes patients who were swabbed once. We also fitted an alternative model in which virus clearance by immunity was assumed to be the main mechanism by which immunity cleared infection (see supplemental material, Equations S1).

### Linear regression model

We regressed log10-transformed URT viral loads against day of symptom onset. For these linear regression models, the intercept describes the URT viral load at the time of symptom onset (virus peak), the slope represents the rate of virus clearance. As we did not have URT viral load measurements taken during the early stage of infection, it is unclear if the time of symptom onset corresponds to the time of virus load peak in virus growth trajectory. We used the same categorisation method for patients, such that they are stratified by age, vaccine brand and doses received, and time since last vaccine dose. We then performed separate linear regressions for each patient subgroup.

Model fitting was performed in R version 4.1.3 and RStan version 2.21.7. Analyses were performed in R version 4.3.1.

## Results

### Data descriptions

URT viral load measurements were taken from community samples during the Delta and Omicron waves in Singapore (S1 Fig). Patients were categorised according to age, time since vaccination, and vaccine group. Vaccine group is determined by the brand(s) of COVID-19 vaccine and number of doses received. We herein refer to a vaccine group and its time since vaccination subgroup as a vaccination history. The number of measurements per vaccination history for each age subgroup is summarised in Table 2. Patients presented to clinics to conduct a nasopharyngeal swab after symptom onset (S2 Fig).

**Table 2:** Summary of number of viral load data points for each vaccination history and age subgroup for Delta and Omicron infections

		Delta								Omicron								
VACCINE GROUP	Age group (years)	Number of viral load data points								Number of viral load data points								
UNVACCINATED	0–4	926								5007								
	5–11	884								2027								
	12–17	525								57								
	18–39	1100								135								
	40–60	1225								152								
	> 60	1502								218								
		Time since last dose (days)								Time since last dose (days)								
		0–7	8–14	15–28	29–60	61–90	91–180	181–270	271–360		0–7	8–14	15–28	29–60	61–90	91–180	181–270	271–360
2-dose Pfizer (PFIZER 2)	5–11	-	-	-	-	-	-	-	-		119	67	64	-	-	-	-	-
	12–17	13	-	30	262	887	836	15	-		-	-	-	33	33	391	758	-
	18–39	94	46	309	1068	870	861	943	173		-	-	-	37	104	2667	1008	128
	40–60	80	49	171	1050	940	905	991	41		-	-	-	18	49	778	468	34
	> 60	81	81	167	769	1066	1151	1009	-		-	-	-	42	77	1124	389	174
2-dose Moderna (MODERNA 2)	18–39	21	-	25	584	917	849	353	-		-	-	-	-	10	434	290	-
	40–60	-	-	-	175	943	876	513	-		-	-	-	-	-	96	145	-
	> 60	-	-	-	54	118	1017	152	-		-	-	-	-	-	111	69	12
2-dose Sinovac (SINOVAC 2)	18–39	-	17	48	549	907	899	38	-		-	-	-	13	12	21	-	-
	40–60	26	16	37	304	619	499	18	-		-	-	-	16	12	11	-	-
	> 60	-	-	19	99	158	109	-	-		-	-	-	44	31	29	-	-
2-dose Sinopharm (SINOPHARM 2)	18–39	28	22	44	41	14	139	53	-		-	-	-	-	22	29	-	-
	40–60	33	34	47	60	-	64	37	-		-	-	-	10	35	36	-	-
	> 60	40	21	45	35	-	-	-	-		-	-	-	-	31	55	-	-
3-dose Pfizer (PFIZER 3)	12–17	-	-	-	-	-	-	-	-		29	-	-	-	-	-	-	-
	18–39	323	140	164	162	-	-	-	-		278	273	511	801	351	603	-	-
	40–60	842	363	372	412	-	-	-	-		102	94	185	644	957	1082	-	-
	> 60	1039	677	876	898	175	-	-	-		123	109	203	601	898	7814	-	-
3-dose Moderna (MODERNA 3)	18–39	73	-	-	-	-	-	-	-		96	79	164	301	49	-	-	-
	40–60	127	43	25	12	-	-	-	-		35	22	57	236	279	121	-	-
	> 60	66	32	36	47	-	-	-	-		15	-	27	79	99	403	-	-
3-dose Sinovac (SINOVAC 3)	18–39	86	75	72	-	-	-	-	-		-	-	13	75	83	30	-	-
	40–60	42	40	-	11	-	-	-	-		-	-	-	42	33	-	-	-
	> 60	-	-	-	-	-	-	-	-		-	-	14	50	59	19	-	-
3-dose Sinopharm (SINOPHARM 3)	> 60	-	-	-	-	-	-	-	-		11	-	16	-	-	-	-	-
2-dose Pfizer, Moderna booster (PPM)	18–39	45	16	21	14	-	-	-	-		108	98	201	352	70	58	-	-
	40–60	120	33	36	37	-	-	-	-		20	18	47	158	150	118	-	-
	> 60	47	29	26	75	-	-	-	-		13	-	14	50	61	344	-	-
2-dose Moderna, Pfizer booster (MMP)	18–39	-	-	-	-	-	-	-	-		29	22	47	100	19	-	-	-
	40–60	56	23	14	-	-	-	-	-		-	-	17	91	90	40	-	-
	> 60	18	-	-	-	-	-	-	-		-	-	-	23	24	79	-	-

### Regressing URT viral load against day of symptom onset

URT viral load measurements were regressed against day of symptom onset, with patients categorised by vaccination history and age group (S1 Table). Measured peak viremia and clearance rate were inferred from the intercept and gradient of linear regression respectively. Delta infections tended to have higher measured peak viremia and faster rate of virus clearance than Omicron infections. With increasing time since last vaccine dose received, we observed a slower rate of virus clearance but no clear trend for measured peak viremia in both Delta and Omicron infections. We observed that generally for both VOCs, older age groups have higher measured peak viremia across vaccine histories. Although older age groups were observed to have slower rate of virus clearance for Delta infections, there was no such trend observed for Omicron infections.

### Mechanistic models and model selection

In addition to exploring the dataset using linear regression models, we developed a mechanistic model to understand the acute infection process. We considered four ODE within-host models with parameters varied at different levels (VOC-specific, vaccination history-specific and age modified, vaccination history-specific) that were fitted in a Bayesian framework to the URT viral load data (S2 Table). Virus-related parameters of infection rate of target cells (β) and natural clearance rate of virus (c) were assumed to be intrinsic to the infecting variant, and were fitted as VOC-specific in all four models. Hence, individuals infected with the same VOC would have the same parameter estimates for virus-related parameters, regardless of vaccination history and age. Meanwhile, immune-related parameters of growth rate of immunity during infection (ω) and infected cell clearance by immunity (γ) were fitted as either vaccination history-specific or age modified, vaccination history-specific.

Akaike Information Criterion (AIC) [31], corrected Akaike Information Criterion (AICc) [32], Bayesian Information Criterion (BIC) [33] and median log-likelihood are used to compare model performance. For all metrics, the closer the score to 0, the better the model fit to each dataset. Model types 2 and 4 had the best fit, with scores closest to 0 across all metrics for both Delta and Omicron datasets (Table 3). Both of these models had growth rate of immunity (ω) fitted as age modified, vaccination history-specific, while infected cell clearance by immunity (γ) was fitted as age modified, vaccination history-specific for model type 4 and without age modification for model type 2. These results indicate that minimally the growth rate of immunity parameter (ω) had to be age-dependent in order to recreate differences between different patient subgroups. Comparing model types 2 and 4, estimating the immune clearance parameter (γ) as age-modified slightly improved model fit (Table 3, Model type 4). However, we found that it was not essential to recreate differences between age groups. Posterior distributions of the age modifier (θ) for immune clearance parameter (γ) showed that age modifier values were close to or overlapped with the reference value of 1.0 (S4 Fig), implying that there was little difference in immune clearance parameter (γ) across age groups for the same vaccination history. Given that model type 2 was sufficient to recreate differences between patient subgroups, we selected model type 2 for its ease of interpretability.

**Table 3:** Summary of goodness-of-fit

Dataset (Infecting VOC)	Model	Number of estimated Parameters	AIC	AICc	BIC	Median LL
Delta	(1) VH: γ, ω	100	676,217	676,217	677,078	-338,008
	(2) VH: γ, AM: ω	105	669,661	669,661	670,565	-334,725
	(3) VH: ω, AM: γ	105	675,683	675,683	676,587	-337,736
	(4) AM: γ, ω	110	669,351	669,352	670,299	-334,565
Omicron	(1) VH: γ, ω	100	618,031	618,032	618,887	-308,915
	(2) VH: γ, AM: ω	105	615,663	615,664	616,562	-307,726
	(3) VH: ω, AM: γ	105	617,840	617,841	618,739	-308,815
	(4) AM: γ, ω	110	615,560	615,560	616,501	-307,670

Akaike Information Criterion (AIC), corrected Akaike Information Criterion (AICc), Bayesian Information Criterion (BIC) and median log-likelihood (LL) of the 4 model variants, for each VOC. VH refers to vaccination history-specific level; AM refers to age modified, vaccination history-specific level.

**Fig. 2:**
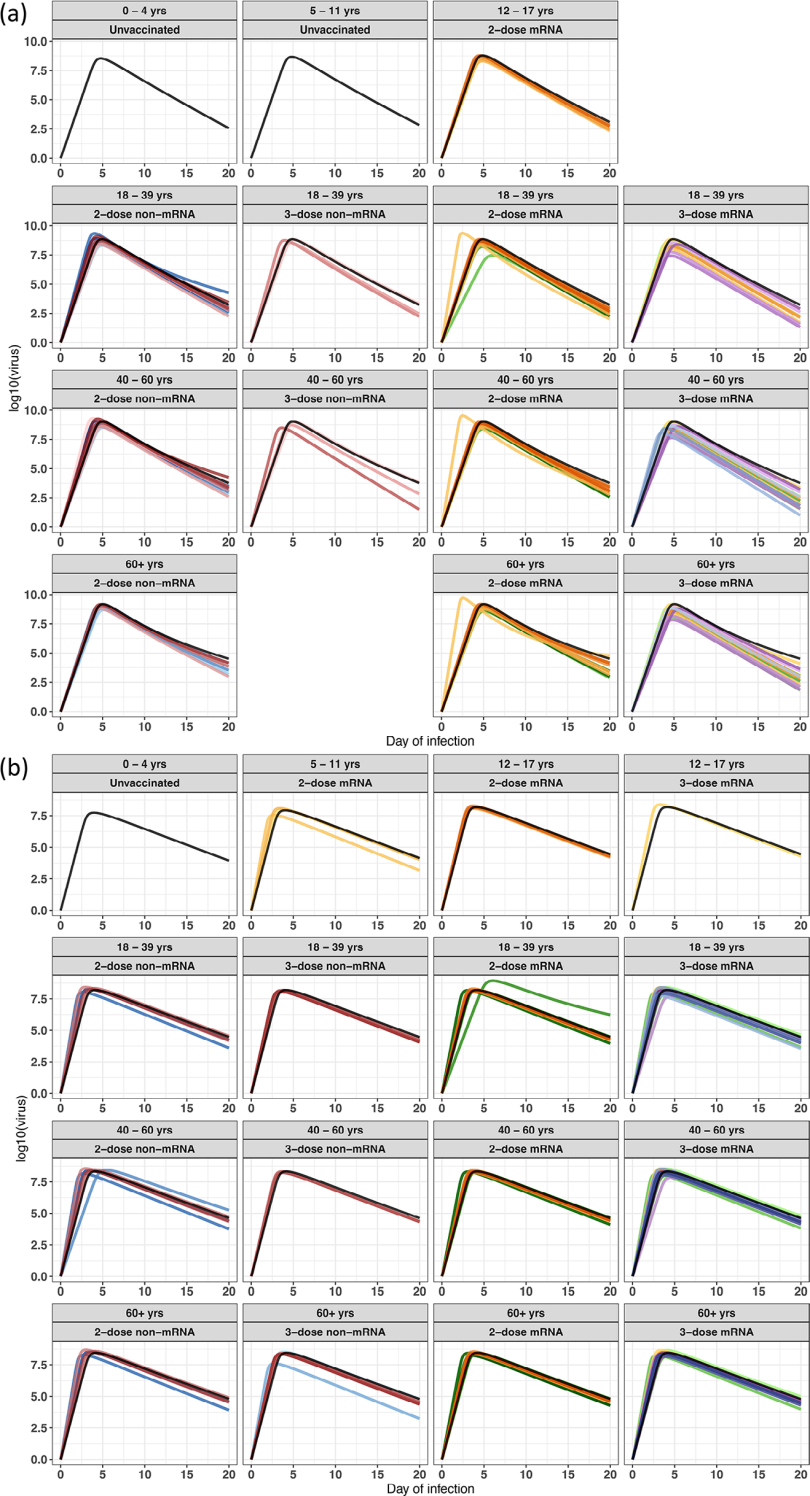
Posterior median viral load trajectories from Model 2 fit, stratified by vaccination history and age subgroup. mRNA-based vaccines are represented by the following colour gradients; yellow to orange for Pfizer-only groups, light green to emerald for Moderna-based groups, lilac to dark purple for PPM, and light blue to navy for MMP. Non-mRNA-based vaccines are represented as follows; light blue to navy for Sinopharm groups, and pink to maroon for Sinovac. Colour gradient scales with increasing time since last vaccinated. Unvaccinated groups are shown in black, and plotted as a reference. (**a**) Delta infections (**b**) Omicron infections. (See Fig S6 for plot with samples from posterior)

Resulting URT viral dynamics based on the parameter estimates of model type 2 for all vaccination histories and age subgroups were computed (Fig. 2). URT viremia in Omicron infections peak earlier than for Delta, although with a lower virus peak value. Moreover, Omicron infections have faster virus proliferation but slower virus clearance compared to Delta infections (Fig. 2). These model dynamics corroborate with the results of regressing URT viral RNA against day of symptom onset (S1 Table) in which Omicron infections were noted to have slower rate of virus decrease and lower viremia levels before decrease than for Delta infections. Both Delta and Omicron infections were observed to have an increasing peak virus value with age. Although rate of virus decay for Delta infections decreases with age, no such trend was observed for Omicron infections, similar to the results of regression for rate of virus decrease. For Delta infections, vaccinated groups have decreased overall viremia compared to unvaccinated counterparts, as indicated by the former’s virus trajectories being below that of the latter. Furthermore, 3-dose mRNA-vaccinated groups have lower viremia than the other doses and vaccine type groups. Comparing 2-dose mRNA and non-mRNA groups, the mRNA groups have reduced overall URT viremia, with majority of the virus trajectories being below the reference line. However, virus dynamics of Omicron infections are largely similar across vaccination histories of the same vaccine group, with only slight differences in time and magnitude of virus peak.

### Modelled components of immunity

In our selected model type, infected cell clearance by immune response (γ) and growth rate of immunity during infection (ω) both varied by vaccination history while the latter also varied by age. Doing so allowed us to examine differences between vaccine brands, doses, and across time since vaccination for both parameters, and age effects on growth rate of immunity. All parameter estimates for infected cell clearance by immune response (γ) and growth rate of immunity during infection (ω) were estimated independently, with no relationship between time since vaccination subgroups.

**Fig. 3:**
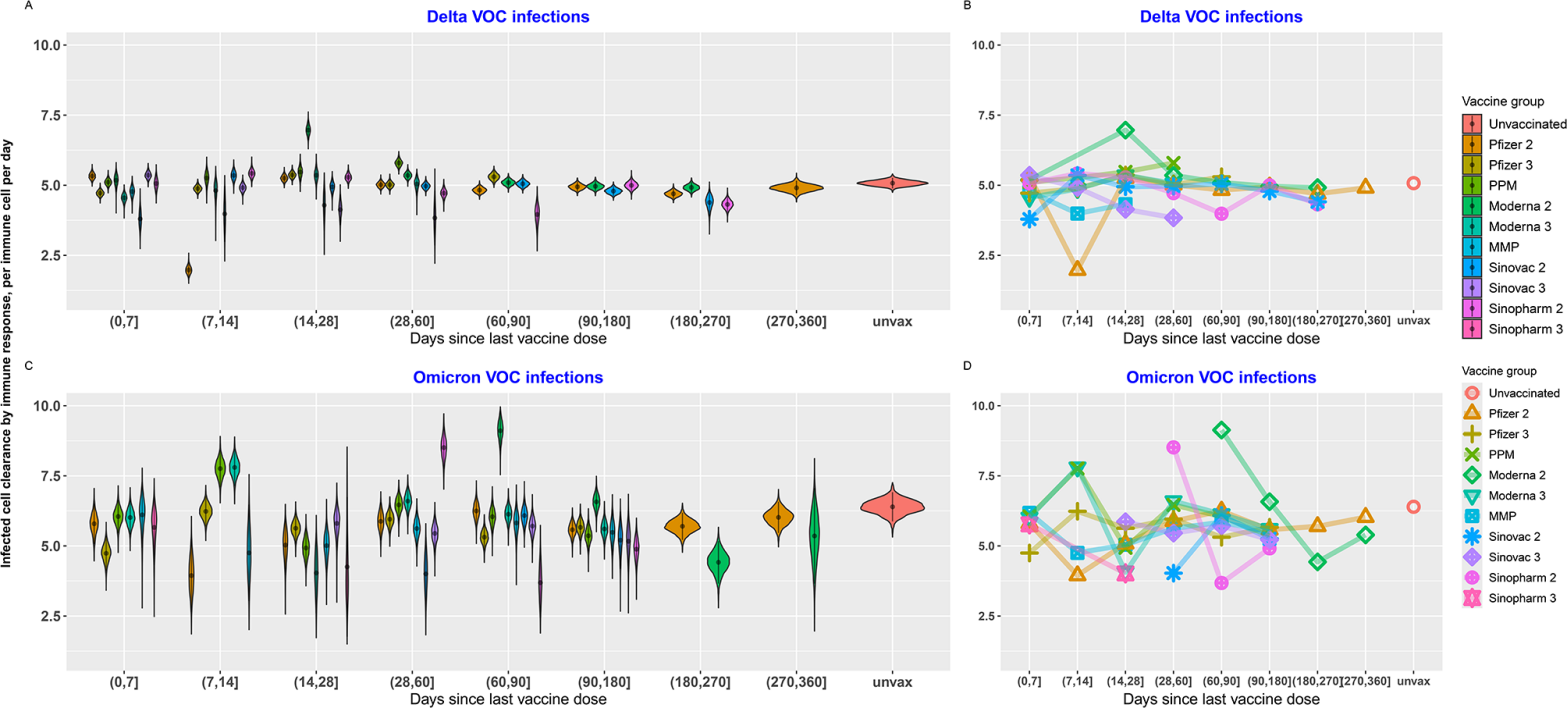
Distribution of parameter estimates for immune clearance of infected cells (γ) for each vaccination history of Model type 2 (**A** and **C**). Coloured lines connect the median values of immune clearance of infected cells (γ) for each vaccine and time since vaccination subgroup (**B** and **D**)

In vaccine histories with data available 6–9 months post-vaccination, infected cell clearance by immune response (γ) was observed to stabilise at a value similar to that of the unvaccinated group after 4 weeks since receiving the last vaccine dose (Fig. 3). For Delta infections, vaccinated group estimates for immune clearance parameter (γ) are comparable to the unvaccinated group. However, for Omicron infections vaccinated groups tended to have lower immune clearance parameter (γ) value than the unvaccinated group (Fig. 3).

**Fig. 4:**
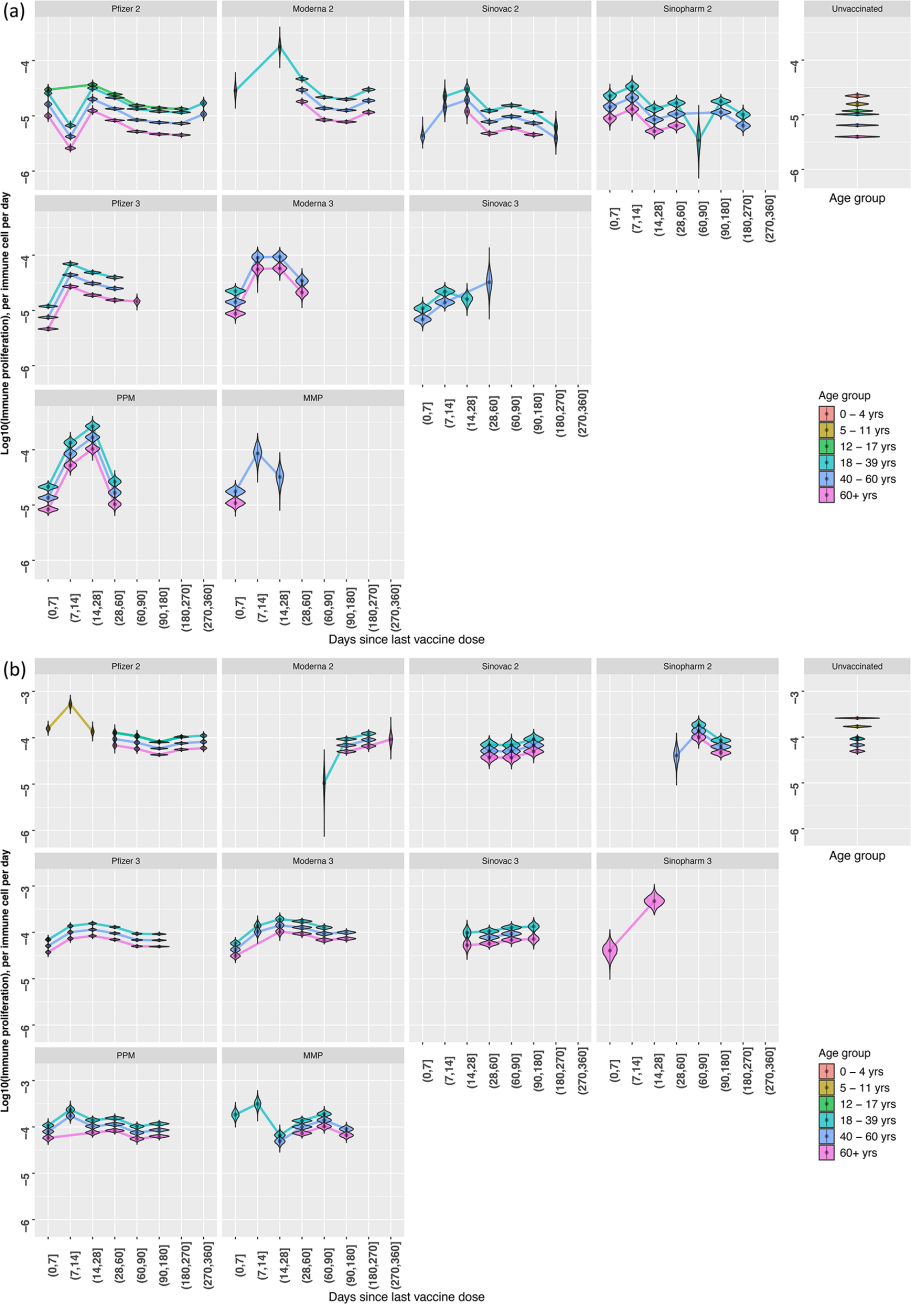
Distribution of growth rate of immunity during infection (ω) estimates for each vaccination history and age subgroup, plotted on the log10 scale. Coloured lines connect the estimated median value of the growth rate of immunity estimate for the given subgroup. Unvaccinated group facet shows estimates for growth rate of immunity for each age group. (**a**) Delta infections, (**b**) Omicron infections

Posterior distributions of the age-modifier for growth rate of immunity (θ) values for Delta and Omicron infections showed a decreasing trend in age modifier value with increasing age (S5 Fig). Age modifier values are highest for the 0–4 years age group (median values for Delta and Omicron: 2.17 and 2.84) and lowest for the 60 + years age group (median values for Delta and Omicron: 0.388 and 0.537). For both VOCs, posterior distributions of the age modifier for the 12–17 years age group are close to or overlap with reference value of 1 (S5 Fig), indicating little to no difference in age effects on growth rate of immunity during infection (ω) from the 18–39 years reference group. For both Delta and Omicron infections, vaccine groups with sufficient datapoints show an increasing, decreasing, then plateauing trend with increasing time since vaccination (Fig. 4). A more gradual increasing then decreasing trend was observed in Omicron infections with growth rate of immunity beginning to plateau at 61–90 days post vaccination, later than Delta infections’ 28–60 days. For Delta infections, growth rate of immunity (ω) is broadly greater in those vaccinated after 14 days of having received their last vaccine dose, as compared to unvaccinated counterparts of the same age group. However, growth rate of immunity (ω) of the vaccinated is similar to or slightly greater than the unvaccinated for the same age group.

### Differences between Delta and Omicron

Next, we compared parameter estimates and within-host basic reproduction numbers, R_0,within_., for Delta and Omicron infections. R_0,within_ is calculated from parameter estimates of each VOC. This is a measure of within-host viral fitness representing the infection potential of a virus that indicates whether an infecting viral population will establish itself in the host.

**Fig. 5:**
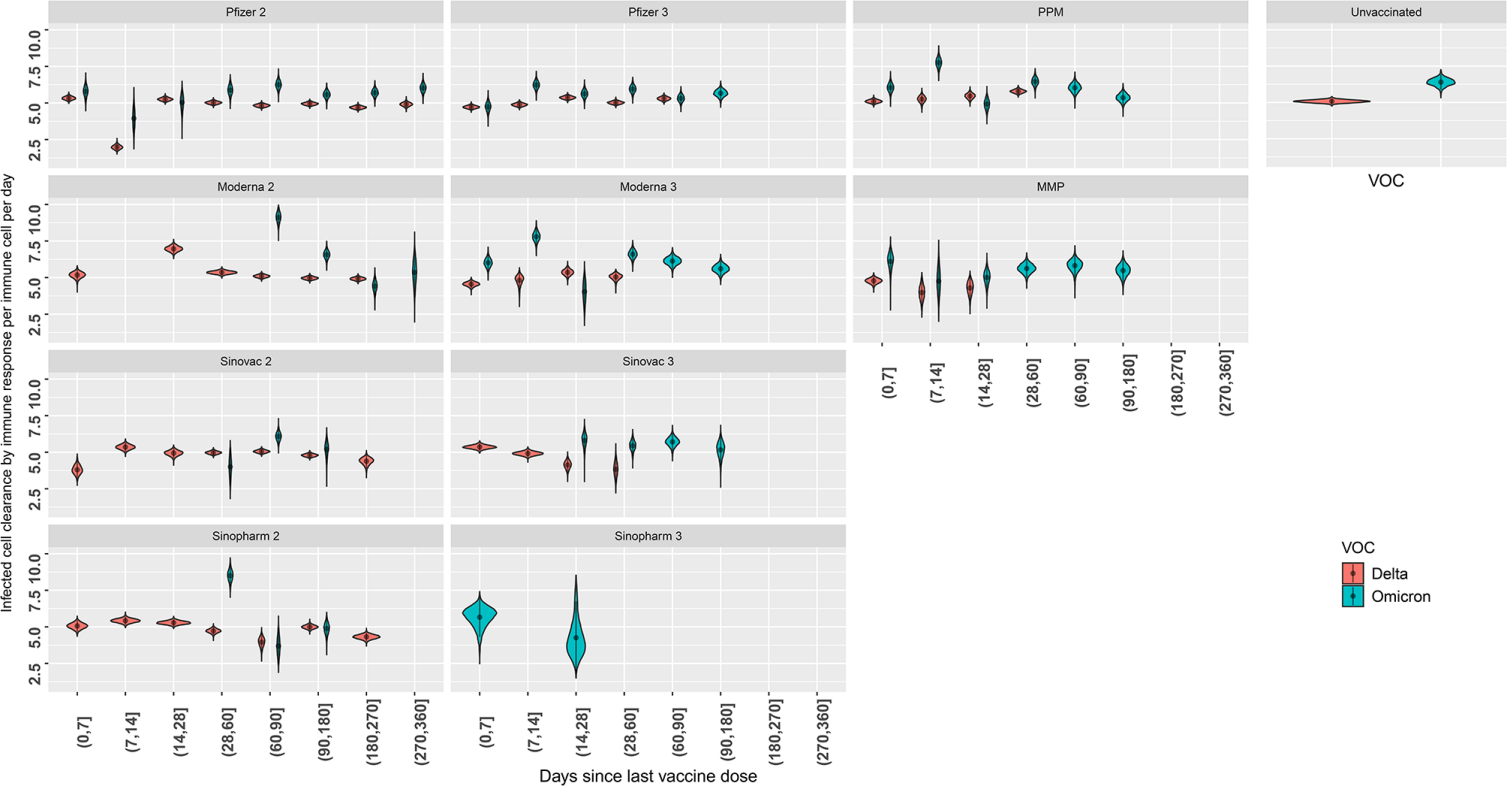
Distribution of estimates for immune clearance of infected cells parameter (γ) for each vaccination history, for Delta and Omicron

**Fig. 6:**
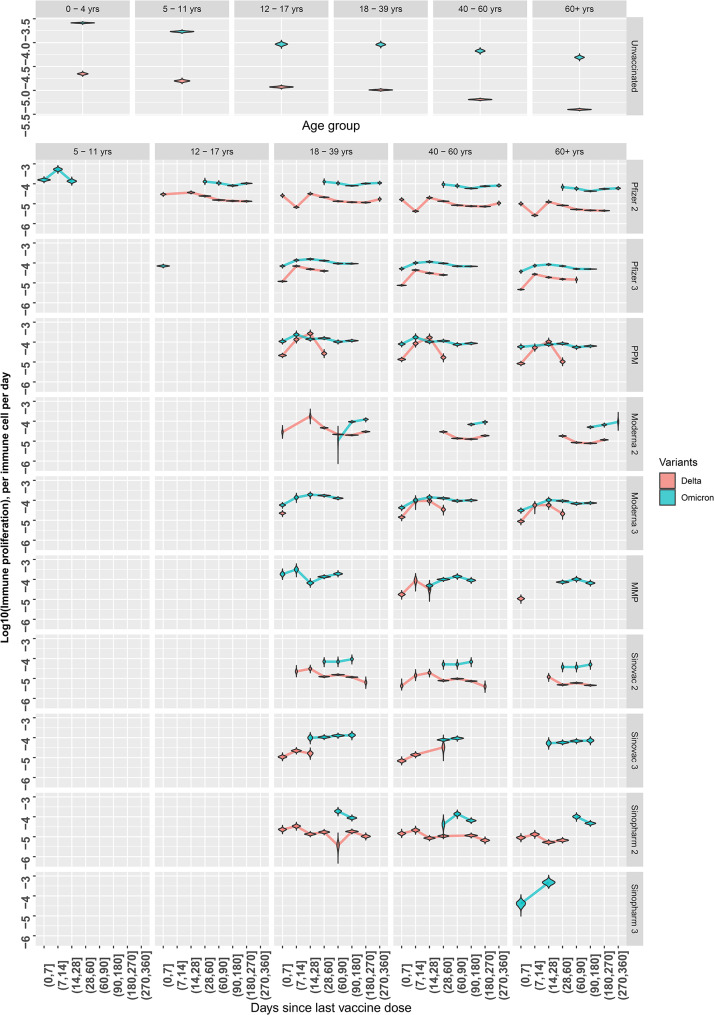
Estimates for growth rate of immunity during infection (ω) for each vaccination history and age subgroup for Delta and Omicron infections, plotted on the log10 scale. The first row shows the unvaccinated group’s estimates for immune proliferation for Delta and Omicron, split by age. Coloured lines connect the median values of immune proliferation estimates for each subgroup

We found that for the same patient subgroups, values for infected cell clearance by immune response (γ) and growth rate of immunity during infection (ω) are greater for Omicron infections (Figs. 5 and 6).

We observed higher virus infection rate of target cells (β (mean [95% CI]): 5.18 [4.78, 5.54] x 10^− 10^ ml^− 1^ day^− 1^ vs. 3.94 [3.82, 4.08] x 10^− 10^ ml^− 1^ day^− 1^) and lower natural clearance rate of free virus (c (mean [95% CI]): 0.81 day^− 1^ [0.80, 0.821] vs. 1.56 day^− 1^ [1.54, 1.57]) for Omicron compared to Delta. R_0, within_ values for Omicron infections are higher than Delta infections (Table 4). Moreover, Delta infections in vaccinated groups have similar R_0, within_ values to the unvaccinated group, while Omicron infections in vaccinated groups have higher R_0, within_ values than the unvaccinated group.

**Table 4:** Estimated R_0,within_ values for each vaccination history, for Delta and Omicron infections

VACCINE GROUP	VOC	*R*_0, within_ value [mean; 95% CI]							
UNVACCINATED	Delta	4.44 [4.40; 4.49]							
	Omicron	8.90 [8.77; 9.05]							
		Time since last dose (days)							
		0–7	8–14	15–28	29–60	61–90	91–180	181–270	271–360
2-dose Pfizer (PFIZER 2)	Delta	4.23 [4.15; 4.33]	11.38 [10.06; 13.00]	4.29 [4.22, 4.37]	4.49 [4.44; 4.55]	4.67 [4.62; 4.73]	4.56 [4.51; 4.61]	4.80 [4.74; 4.87]	4.59 [4.45; 4.78]
	Omicron	9.80 [9.10; 10.98]	14.38 [11.01; 21.40]	11.16 [9.78; 14.45]	9.66 [9.05; 10.65]	9.11 [8.75; 9.57]	10.21 [9.98; 10.45]	9.99 [9.76; 10.24]	9.45 [9.09; 9.91]
2-dose Moderna (MODERNA 2)	Delta	4.35 [4.11; 4.72]		3.24 [3.12; 3.38]	4.22 [4.15; 4.28]	4.43 [4.37; 4.48]	4.54 [4.49; 4.60]	4.59 [4.51; 4.67]	
	Omicron					6.25 [6.00; 6.54]	8.66 [8.48; 8.86]	12.80 [11.39; 15.49]	10.52 [8.38; 15.63]
2-dose Sinovac (SINOVAC 2)	Delta	5.94 [5.15; 6.99]	4.21 [4.03; 4.44]	4.56 [4.34; 4.89]	4.54 [4.47; 4.61]	4.47 [4.41; 4.52]	4.71 [4.64; 4.78]	5.11 [4.75; 5.84]	
	Omicron				14.10 [11.03; 21.32]	9.35 [8.93; 9.88]	10.78 [9.65; 14.00]		
2-dose Sinopharm (SINOPHARM 2)	Delta	4.45 [4.19; 4.79]	4.16 [4.02; 4.32]	4.27 [4.17; 4.38]	4.77 [4.59; 5.02]	5.66 [5.00; 6.89]	4.51 [4.38; 4.68]	5.22 [4.97; 5.56]	
	Omicron				6.69 [6.40; 7.04]	15.39 [11.67; 22.90]	11.57 [10.50; 13.74]		
3-dose Pfizer (PFIZER 3)	Delta	4.78 [4.71; 4.85]	4.62 [4.53; 4.71]	4.20 [4.16; 4.25]	4.49 [4.43; 4.56]	4.25 [4.16; 4.37]			
	Omicron	11.97 [11.06; 13.57]	9.14 [8.87; 9.45]	10.11 [9.75; 10.54]	9.57 [9.34; 9.82]	10.71 [10.38; 11.09]	10.05 [9.87; 10.25]		
3-dose Moderna (MODERNA 3)	Delta	4.94 [4.74; 5.24]	4.63 [4.26; 5.73]	4.20 [3.97; 4.52]	4.45 [4.24; 4.92]				
	Omicron	9.45 [8.99; 10.09]	7.30 [7.10; 7.52]	13.95 [10.76; 22.21]	8.63 [8.41; 8.88]	9.28 [9.01; 9.63]	10.14 [9.68; 10.79]		
3-dose Sinovac (SINOVAC 3)	Delta	4.21 [4.10; 4.34]	4.59 [4.43; 4.78]	5.44 [4.92; 6.29]	5.87 [4.75; 7.84]				
	Omicron			9.69 [8.87; 12.03]	10.41 [9.69; 11.73]	9.96 [9.43; 10.75]	10.89 [9.57; 14.12]		
3-dose Sinopharm (SINOPHARM 3)	Delta								
	Omicron	9.82 [8.73; 13.82]		14.10 [7.75; 24.06]					
2-dose Pfizer, Moderna booster (PPM)	Delta	4.41 [4.32; 4.52]	4.28 [4.03; 4.68]	4.12 [3.92; 4.37]	3.89 [3.82; 3.97]				
	Omicron	9.40 [8.97; 9.97]	7.34 [7.13; 7.58]	11.51 [10.47; 13.20]	8.81 [8.58; 9.07]	9.41 [8.99; 10.03]	10.60 [10.08; 11.36]		
2-dose Moderna, Pfizer booster (MMP)	Delta	4.71 [4.48; 5.07]	5.66 [4.61; 7.54]	5.22 [4.48; 6.77]					
	Omicron	9.18 [8.28; 11.94]	11.95 [8.97; 18.54]	11.25 [9.95; 14.10]	10.10 [9.48; 11.09]	9.70 [9.06; 11.30]	10.33 [9.39; 12.01]		

### Sensitivity analyses

To assess model sensitivity to data limitations, choices of incubation period distribution and infection-clearing mechanism, we conducted sensitivity analyses.

First, we tested model sensitivity to the types of patients with longitudinal samples in the dataset. We created two alternative datasets for model fitting. The first dataset only included the first recorded swab of each individual. The second dataset only included patients who had one swab taken. Results of both model fits showed similar observed differences in parameter estimates between Delta and Omicron infections, vaccination histories, and age groups. However, we observed that virus decay phase for both model fits for Delta and Omicron infections were noticeably slower than in the original model fit (S7 & S8 Figs).

Next, we investigated the model’s sensitivity to chosen value of standard deviation for the normally distributed errors of log viremia measurements. We performed two model fittings with different standard deviations of viral load errors, σ^2^ = 0.5 and σ^2^ = 2. Results of these fittings showed that certain parameters, namely infection rate of target cells (β) and clearance of infected cells by immunity (γ) were sensitive to σ^2^ value. Parameters of natural clearance rate of virus (c), age modifier (θ), and growth rate of immunity (ω) were insensitive (S13 & S14 Figs). We note that R_0,within_ values are consequently affected by the σ^2^ value, since it incorporates β and c in its calculation. Despite this, all trends observed for infected cell clearance by immunity (γ) and growth rate of immunity (ω) were conserved regardless of assumed value of σ^2^.

Thirdly, to investigate model sensitivity to incubation periods for Delta and Omicron infections, we fitted our model to data with switched distributions for incubation periods of Delta and Omicron infections. The model fit to dataset with swapped incubation periods maintained similar magnitudes of virus peak and rate of virus decay to the original model fits (S9 Fig). Observed differences and trends in immunity-related parameters for vaccination histories and age subgroups were also robust. However, there was a marked difference in the time of virus peak for both Delta and Omicron infections, with Delta infection peaking earlier and Omicron infection peaking later than in the original model fit. These results indicate that the early stages of infection in our model are sensitive to our choice in incubation period.

Lastly, to assess our choice in infection-clearing mechanism by immunity, we developed and fitted an alternative model in which clearance of free virus by immunity was assumed to be the main mechanism by which immunity controls infection (see supplemental material, Equations S1). Model diagnostics for this alternative model were poor (S10 Fig) and it was unable to recreate virus dynamics. These results justify our choice of model with clearance of infected cells by immunity as the main mechanism.

## Discussion

From our initial data exploration via the regression analysis (S1 Table), we observed higher measured peak viremia in Delta infections, faster rate of virus clearance in Delta infections, and slower rate of virus clearance in older age groups, similar to the results of the within-host model. The mathematical model further allowed us to examine dynamics of both virus and immune dynamics during infection, and their interplay.

We have constructed a simple mathematical model describing the within-host dynamics of infection with SARS-CoV-2, fitted to URT viral load data collected in Singapore. We characterised heterogeneities in infection dynamics across vaccine histories and ages by fitting immunity-related parameters as vaccination history-specific, with or without age-modification. Moreover, by varying virus-related parameters by infecting VOC, it was possible for us to recreate differences between Delta and Omicron infections.

Resulting URT virus dynamics from model fitting showed rapid virus growth, early peak, followed by gradual decay in circulating virus. The shapes of our model fits for URT viremia (Fig. 2) are broadly similar to that observed in a human challenge trial with wild-type virus for both nasal and throat viral load trajectories [34]. Despite us fitting our models to individual data where a majority of the individuals only had one nasopharyngeal swab taken, by estimating parameters at a group level, we were able to recreate similar trends in URT virus dynamics observed in other within-host models of URT SARS-CoV-2 infection fitted to longitudinal patient data [15, 17, 25]. The Bayesian approach we adopted made it possible for us to utilise our dataset which included a large number of individuals with only one measurement taken during acute infection. Hence, it is possible for our model to be used for other swab datasets with a large number of patients, but without repeated measurements allowing exploration of the impact on virus dynamics of a wide range of vaccination histories and ages.

Model selection results showed that it was necessary to vary growth rate of immunity during infection (ω) by age and vaccination history to reproduce differences observed between patient subgroups (Table 3). As a result of this age modifier, growth rate of immunity (ω) decreased with increasing age for the same vaccination history, suggesting weakened immune function in older age groups (Fig. 4). These results provide supporting evidence for existing hypotheses on the role of immunosenescence in SARS-CoV-2 infection in older individuals [35–37]. We further noted that for a given age group, growth rate of immunity (ω) remained higher for vaccinated groups compared to the unvaccinated regardless of time since vaccination. These results in Fig. 4 align with findings that although both older and younger age groups had raised antibody responses following vaccination, antibody titres were lower in older age groups than in younger individuals despite vaccination [38, 39]. We found that clearance by immune response (γ) could be varied by vaccination history without the need for age modification (Table 3). Although there was a clear trend over time since vaccination for growth rate of immunity (ω), no trend over time since vaccination was observed for immune clearance (γ). This suggests that there are little changes in the function of clearing immunity over time since vaccination and instead, time since vaccination is perhaps more relevant to the activation of immunity given infection. However, this conclusion remains tentative with the lack of immunological measurements in our data. Overall, our model is able to recreate overall differences in parameter estimates for growth rate of immunity (ω) with differing vaccination history and age.

By considering time since last vaccine dose received as a means of subgrouping, we are able to capture differences in infection dynamics across time since vaccination subgroups that was possibly due to waning immunity. We found that both Delta and Omicron infections showed increasing growth rate of immunity during infection (ω) for the first 2 weeks post-vaccination before decreasing, then finally plateauing from approximately 60–90 days post-vaccination. For Delta and Omicron, growth rate of immunity (ω) at 0–7 days for a vaccine group and age subgroup is typically lower than that at 8–14 days or 15–21 days. These findings are consistent with studies of T-cell and antibody response after 2 doses of mRNA vaccines that showed increased levels after 21 days post-vaccination [40, 41]. Furthermore, Gao et al. reported that antigen-specific effector T-cells contracted to approximately pre-vaccination levels by day 90 post-vaccination [41], with similar declining of antibody titres [42] observed in individuals who completed their primary series of vaccination. Waning of humoral response after third mRNA dose was also observed, albeit at a slower rate than after the second dose [5]. Although we did not model specific components of immunity, trends observed for the generic clearing immunity included in our model appears to mimic these observations of the waning adaptive immune response. In the absence of measurements of the effector immune response we are unable to draw definitive conclusions regarding the reasons for this eventual decline in growth rate of immunity (ω).

Comparing Delta and Omicron infections, model results showed faster virus growth, earlier and lower virus peaks followed by slower virus decline for all Omicron infections (Fig. 2). We found Omicron infections to have higher R_0, within_ values than Delta infections (Table 4), with higher estimates for virus infection rate of target cells (β) and modelled components of immunity, and lower estimates for natural clearance rate of free virus (c). These results corroborate with a previous study using a model of virus infection dynamics of Delta and Omicron variants in nasal and lung cell types, whose results showed Omicron to have greater fitness advantage in human nasal epithelial cells with higher target cell infection rate and faster growth rate of virus, but lower peak viral load than Delta [43]. Although other studies have reported lower viral load for Omicron than Delta [44, 45], results in literature remain conflicting. Some studies have shown Delta and Omicron infections to have little difference in their viral load regardless of vaccination status [46, 47], while others have reported higher viral load for Omicron infections [48]. However, these studies had not accounted for differences in incubation period of infected individuals. As such, their results compare viral load measurements for Delta and Omicron infections regardless of date of symptom onset, with measurements likely to have been taken at different timepoints of individual virus dynamics trajectories. A recent study of Delta and Omicron viral kinetics accounting for time since last infection or vaccination reported higher peak viral loads for Delta infections compared to Omicron (BA.1), similar to our results [49].

We found that vaccination altered URT viral dynamics more in Delta infections than Omicron. The difference in virus dynamics is especially apparent in the 3-dose mRNA vaccine groups that had lowered virus peak value and, in some cases, increased rate of virus decline for Delta infections (Fig. 2). There was no such discernible trend for 3-dose mRNA vaccine groups with Omicron infections, with URT viral load dynamics being similar regardless of vaccine brand. These results align with findings of other studies. Yang et al. found largely similar viral dynamics among individuals who were unvaccinated, received 2 doses of inactivated vaccine, and received 3 doses of inactivated vaccine during Omicron infection [50]. A study on Delta and Omicron virus dynamics showed a greater difference in viral load of the 2-/3-dose vaccine groups compared to those unvaccinated for Delta infections compared to Omicron [44]. However, Puhach et al. found that although vaccination decreased both RNA and infectious viral loads for Delta infections, the same was not observed for Omicron infections. The study showed that for Omicron infections, although 3-dose vaccinated individuals had lower infectious viral loads than 2-dose vaccinated individuals, RNA viral load of the two groups were largely similar [51]. Moreover, we found that Omicron infections in vaccinated groups had greater R_0,within_ values and lower value for clearance of infected cells by immunity (γ) compared to the unvaccinated groups. Our results show that vaccination was not able to subdue virus dynamics in Omicron infections as well as it had for Delta infections. This is possibly because the vaccines included in this study were the monovalent COVID-19 vaccines that did not confer as good protection against Omicron infections compared to past VOCs. As such, our results motivate for a vaccine that is better able to reduce Omicron virus load while minimising side effects.

The model has several limitations. First, we fitted our models to nasopharyngeal viral load measurements obtained post-symptom onset. Due to the lack of data during the early stages of infection, assumptions regarding incubation period, virus production rate and uninfected target cell density affect estimates of infection rate of target cells (β). Moreover, the large number of patients in our dataset resulted in our inability to estimate individual-level incubation periods during model-fitting, and in us assigning individual incubation periods. Sensitivity analysis showed that the early stages of infection in our model are sensitive to the assumed distributions of incubation period, corroborating with findings of Ke et al. [52]. We observed that some vaccinated groups had steeper virus increase and higher peak viremia compared to unvaccinated counterparts, which is likely due to this uncertainty in early infection dynamics and small numbers in some groups. As such, in the absence of early-stage viral load data, we are limited in our ability to model variations in early virus dynamics between vaccination regimes and age groups, with our conclusions pertaining to early virus dynamics remaining tentative. To address this issue, further studies can be conducted with data including measurements taken prior to symptom onset.

Second, we are characterising infection in patients with a narrow range of symptoms. The clinical manifestations of disease experienced by the patients in our dataset were likely to be mild to moderate. Individuals in the dataset presented to clinics for confirmatory testing of SARS-CoV-2 infection based on nasopharyngeal PCR swabs. This implies that individuals perceived their symptoms, but these symptoms were not sufficiently severe for individuals to present to hospitals or other forms of emergency care. Hence when making inferences regarding differences between vaccinated and unvaccinated groups, and between vaccine groups, we note that we are studying breakthrough infections, and that vaccine efficacy might differ between vaccine groups.

Third, majority of individuals in our dataset were swabbed once, with swabs taken earlier in the course of infection, closer to the date of symptom onset. As a result, these individuals mostly contribute to data closer to the time of virus peak. Individuals with longitudinal samples were typically elderly, unvaccinated individuals, or individuals vaccinated with non-mRNA vaccines who had multiple swabs taken as per the swab protocol. Sensitivity analysis by model fitting showed model fits without longitudinal data to have slower virus decay phase compared to the original model fit that included longitudinal data. Sampling from the posterior for these model fits showed similar widths of virus trajectories (S11 & S12 Figs). Hence in this study, it remains uncertain if longitudinal samples assist in informing the virus decay phase, or counterintuitively bias the model towards having a faster virus clearance. To address this limitation, further modelling studies using data in which all patients are longitudinally sampled for similar time periods can be conducted.

Another limitation of this study is the simplicity of the model. Without measurements of the uninfected and infected target cell populations, and effector immune response, we are unable to consider more complex models of infection. For example, we could not explicitly model effector cell populations nor include more than one infection-clearing immune mechanism. Moreover, we are only able to consider a clearing (adaptive) immune response that affects either the infected cell population or the free virus population in our model. In addition, we opted to account for age by incorporating it as a modifier to limit the number of parameters to be fitted in our model and preserving the model’s simplicity. Although this allowed us to clearly compare age effects for growth rate of immunity (ω) via the age modifier (θ), opting for this simpler model resulted in us being unable to capture age-dependent immune priming and waning.

Lastly, we assumed that the main mechanism by which immunity controls infection was infected cell clearance and did not include clearance of free virus by immunity in our model. As such, we are only modelling one arm of immunity, mainly the T-cell mechanisms for infected cell clearance. In our model results, we are unable to distinguish between infected cell clearance and clearance of free virus, as well as between clearance by immunity and natural clearance. We opted to model one mechanism for immune control of infection and fix the natural clearance rate of the component being cleared. In these models, modelled adaptive immunity affects only the infected cell compartment or free virus compartment. However, regardless of the immunity-clearing forms of action used, observed patterns for patient subgroups should uphold and our study findings would remain consistent. Our original model was able to recreate virus dynamics similar in shape to human challenge studies and past modelling work, unlike the alternate model. However, the alternative model’s inability to recreate viral load trajectories is likely due to the lack of data on immunity-related components. Further studies could compare model fits for both models if such data is available alongside viral load measurements during infection.

By fitting a mathematical model of SARS-CoV-2 infection to Delta and Omicron infection data, in which information on patients’ vaccines received, age, and time since last vaccine dose is available, we were able to provide insights into the age effects on immunity, immunity waning, the role of different levels of vaccine-induced protection, and VOC-specific infection dynamics of SARS-CoV-2. Our work highlights the importance of age-targeted public health policy, updating immunity with booster doses, and the need for vaccines better targeted against Omicron infection. A possible extension to this work would involve the comparison of Omicron infections in individuals who received monovalent vaccines with those who received bivalent vaccines to investigate the effects of these vaccines on virus dynamics. Such work could consider infection with different Omicron sublineages if data is available, in order to examine differences in virus dynamics between Omicron variants.

### Electronic supplementary material

Below is the link to the electronic supplementary material.

Supplementary Material 1

Supplementary Material 2

## Data Availability

The data that support the findings of this study are available from the authors upon reasonable request and with the permission of MOH. Restrictions apply to the availability of these data, which were used under license for the current study, and so are not publicly available. R and Stan scripts for analyses and generating of figures are available at https://github.com/ID-Modelling-Lab/Covid_withinhost.

## Abbreviations

AIC: Akaike Information Criterion
AICc: Corrected Akaike Information Criterion
BIC: Bayesian Information Criterion
LL: Log–likelihood
URT: Upper respiratory tract
VOC: Variant of concern
