# Supplementary material for: Differences in virus and immune dynamics for SARS-CoV-2 Delta and Omicron infections by age and vaccination histories
Source: BMC Infect Dis. 2024 Jun 29;24:654. doi: 10.1186/s12879-024-09572-x (PMC11218222; doi:10.1186/s12879-024-09572-x)

Differences in virus and immune dynamics for SARS-CoV-2 Delta and Omicron by age and vaccination histories

Maxine W Tan[1], Anet J.N. Anelone[1], An Ting Tay[2], Ren Ying Tan[2], Kangwei Zeng[2,3], Kelvin Bryan Tan[1,2,4], Hannah Eleanor Clapham[1]
1 Saw Swee Hock School of Public Health, National University of Singapore, Singapore; 2 Ministry of Health, Singapore; 3 National Centre for Infectious Diseases, Singapore, Singapore; 4 Duke-NUS Graduate Medical School, National University of Singapore, Singapore.

Figure S1. COVID-19 case count in Singapore reported to WHO from April 2021 to June 2022, and the number of swabs taken each day in the unfiltered and filtered datasets. COVID-19 case count is represented by the black line, following the primary axis. The number of swabs taken each day is represented by the yellow bars, while the data subset used for our study is represented by the blue bars. Both colours of bar follow the secondary axis.

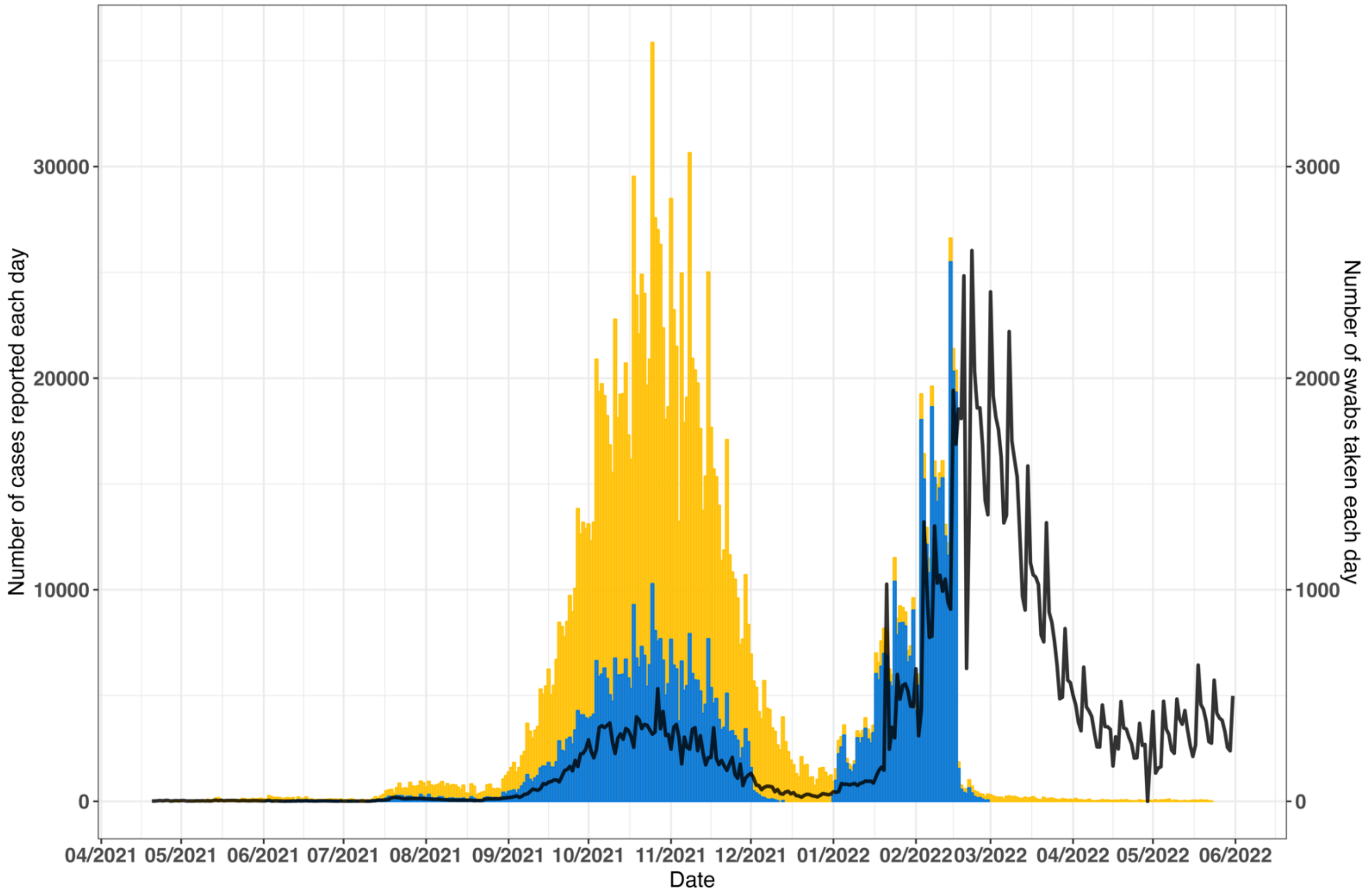

Figure S2. Plots of viral load data over day of symptoms from swabbed individuals included in this study, by vaccine group and age subgroup, with coloured points representing the time since last vaccine dose subgrouping. (a) Delta infections, (b) Omicron infections. Delta infections are defined as cases with date of symptom onset between 1 June 2021 to 30 November 2021, Omicron infections are defined as 1 January 2022 to 18 February 2022.

(a) Delta

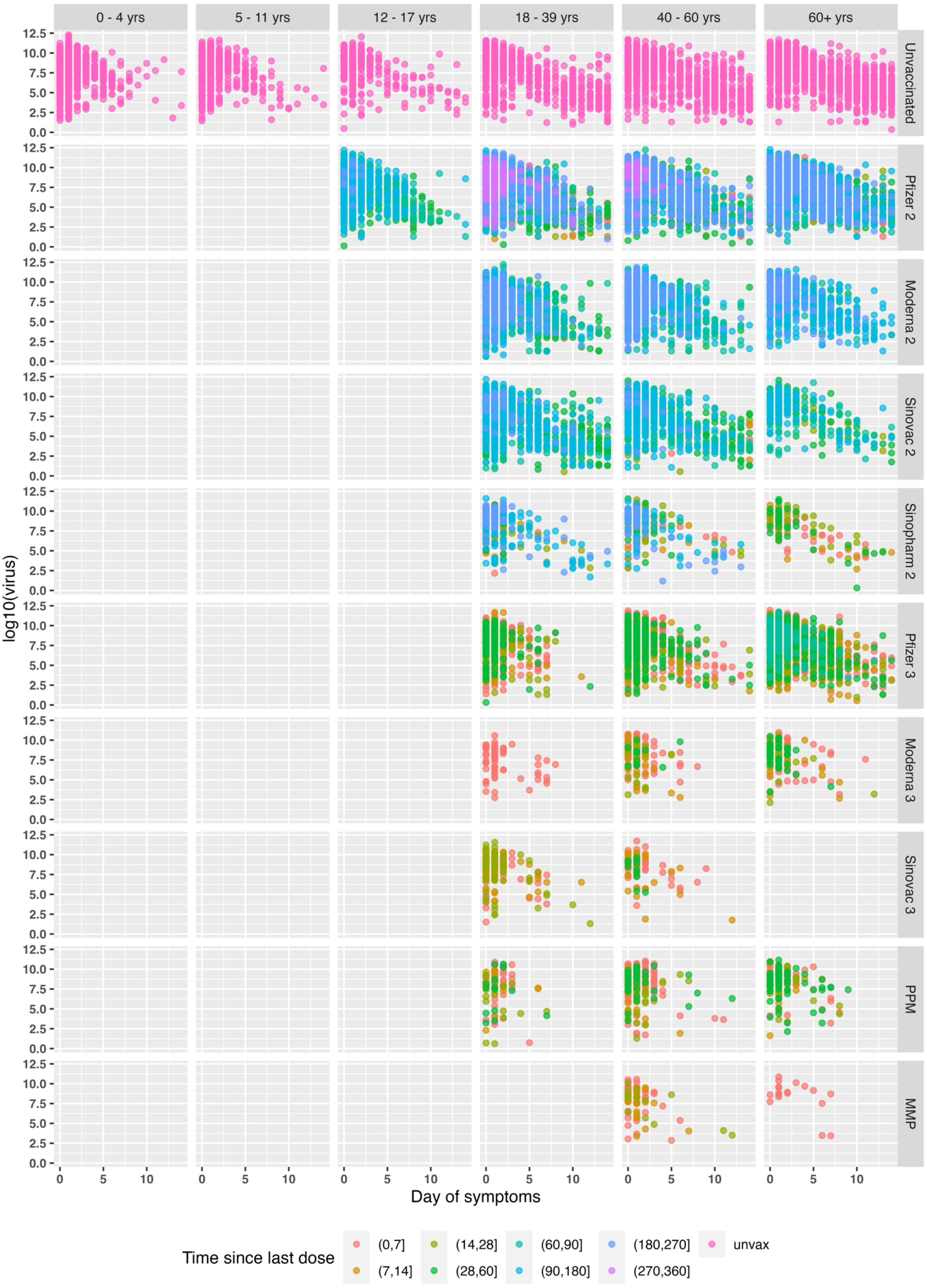
0 - 4 yrs
5 - 11 yrs
12 - 17 yrs
18 - 39 yrs
40 - 60 yrs
60+ yrs
Unvaccinated
Pfizer 2
Moderna 2
Sinovac 2
Sinopharm 2
Pfizer 3
Moderna 3
Sinovac 3
PPM
MMP
log10(virus)
Day of symptoms
Time since last dose
(0,7]
(7,14]
(14,28]
(28,60]
(60,90]
(90,180]
(180,270]
(270,360]
unvax

(b) Omicron

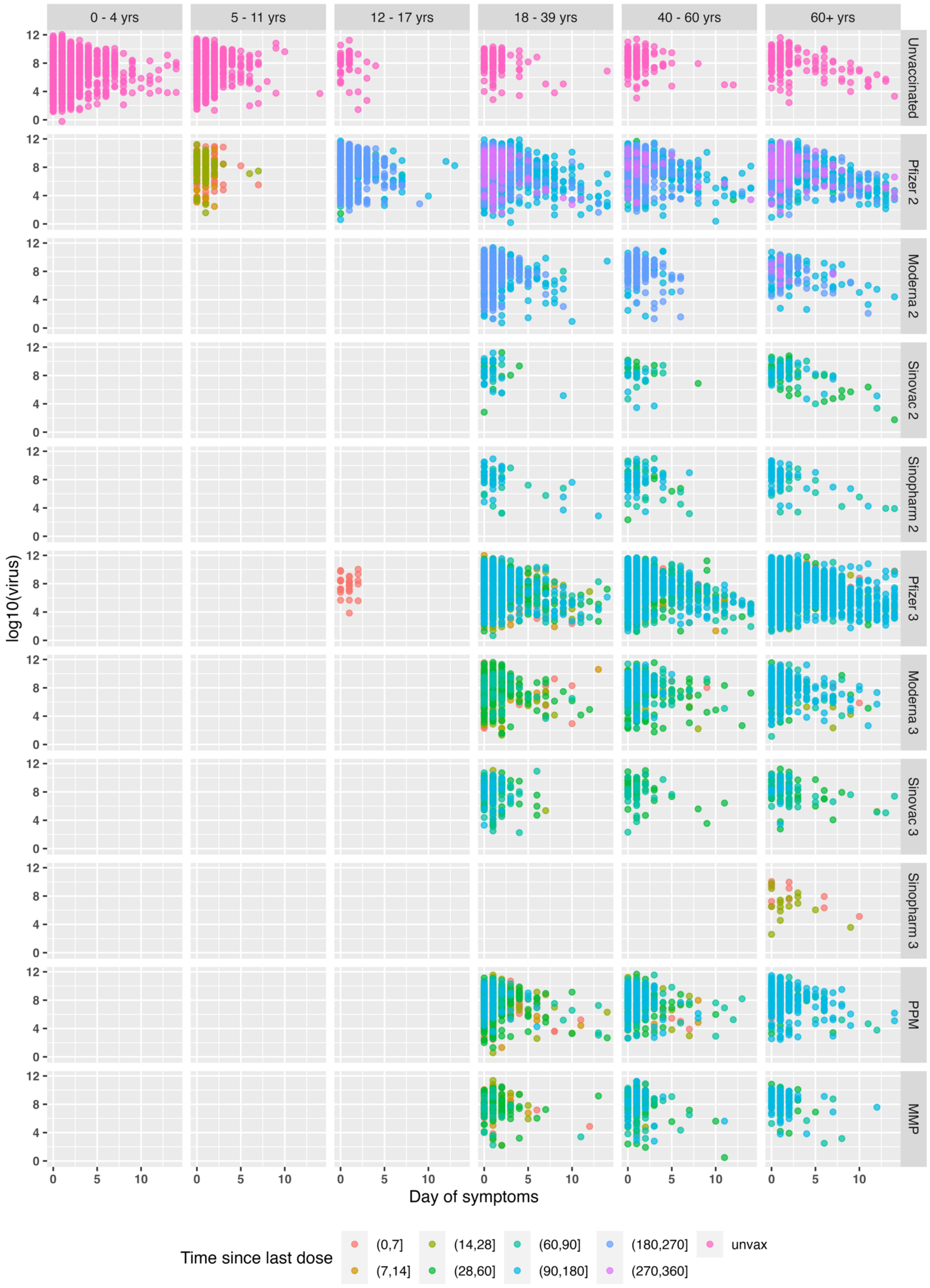

0 - 4 yrs
5 - 11 yrs
12 - 17 yrs
18 - 39 yrs
40 - 60 yrs
60+ yrs
Unvaccinated
Pfizer 2
Moderna 2
Sinovac 2
Sinopharm 2
Pfizer 3
Moderna 3
Sinovac 3
Sinopharm 3
PPM
MMP
log10(virus)
Day of symptoms
Time since last dose
(0,7]
(7,14]
(14,28]
(28,60]
(60,90]
(90,180]
(180,270]
(270,360]
unvax

Fig S3. Model diagnostics for model types 1-4.

(1a) Trace plots for Model Type 1. (Left) Delta infections, (Right) Omicron infections.

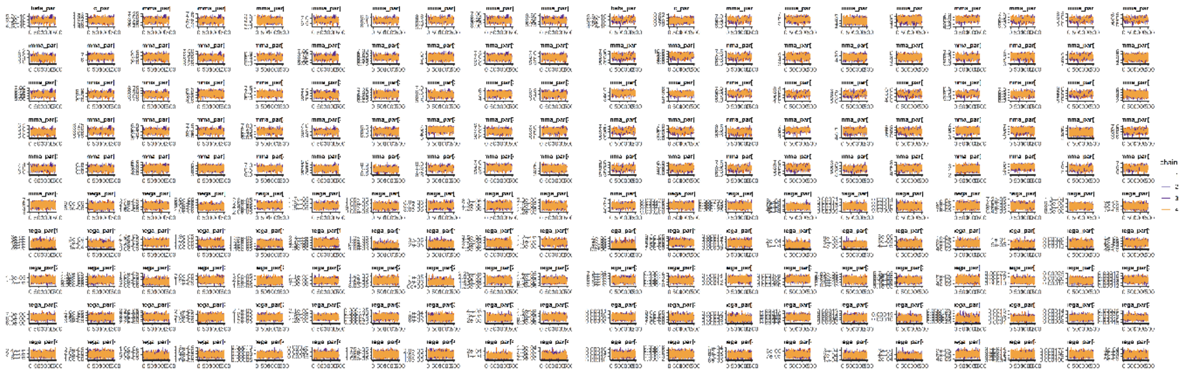

(1b) R-hat plot for Model Type 1. (Left) Delta infections, (Right) Omicron infections.

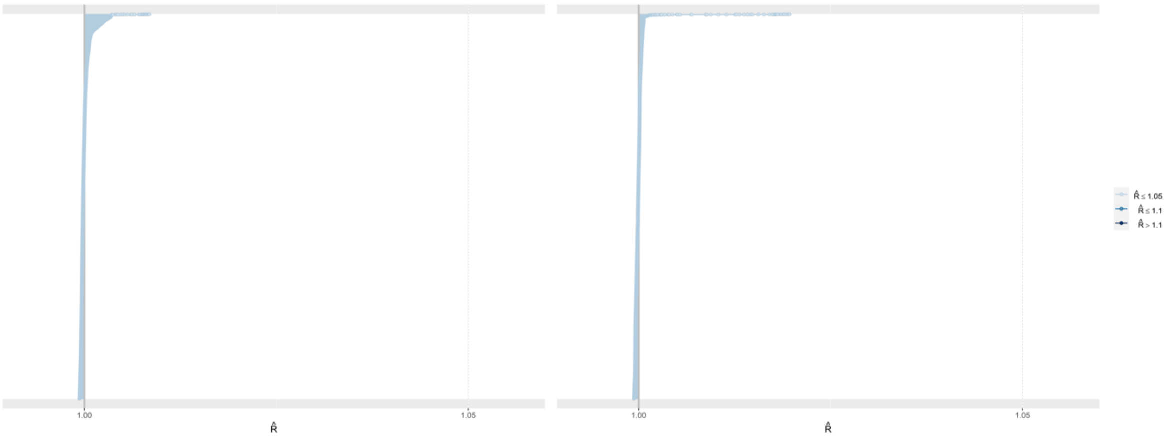

(2a) Trace plots for Model Type 2. (Top) Delta infections, (Bottom) Omicron infections.

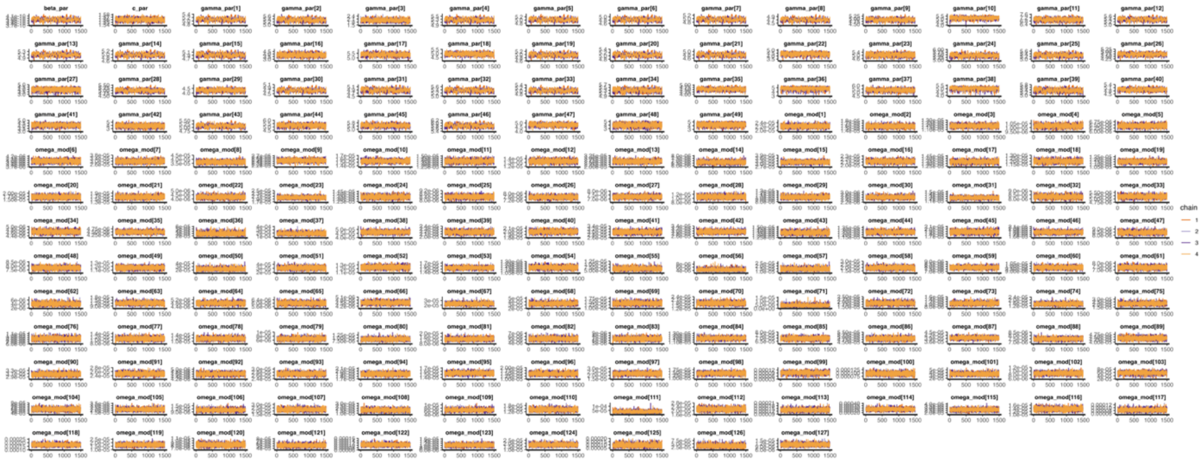

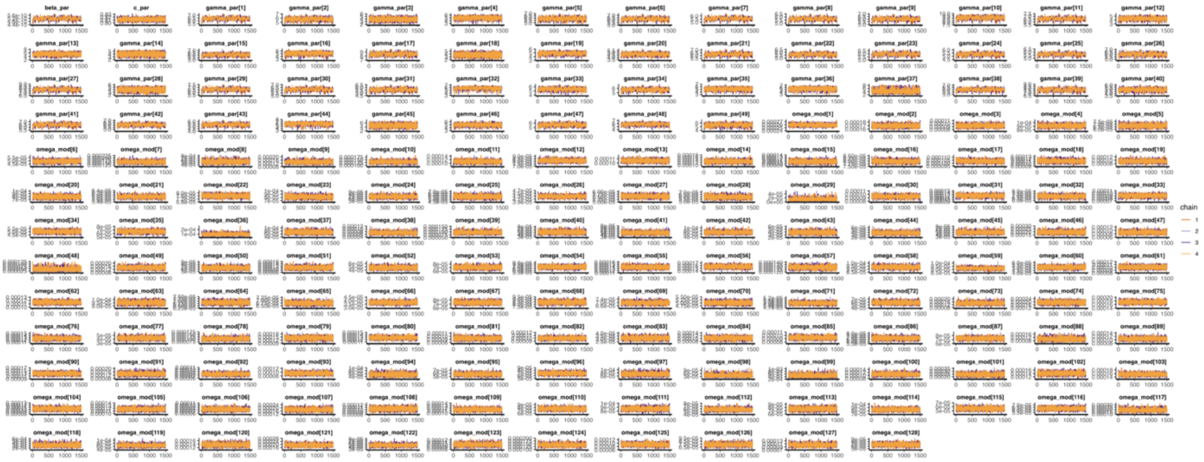

(2b) R-hat plot for Model Type 2. (Left) Delta infections, (Right) Omicron infections.

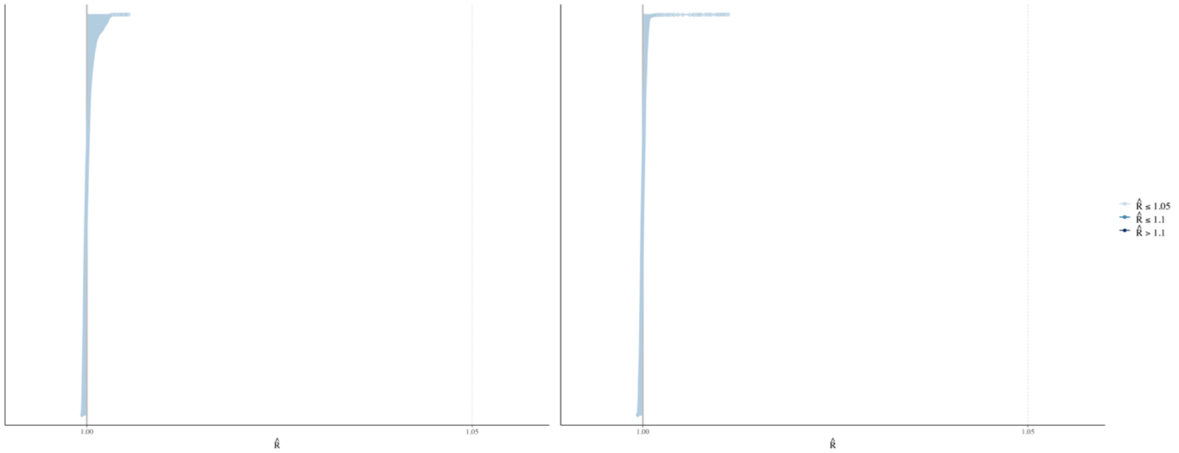

(3a) Trace plots for Model Type 3. (Top) Delta infections, (Bottom) Omicron infections.

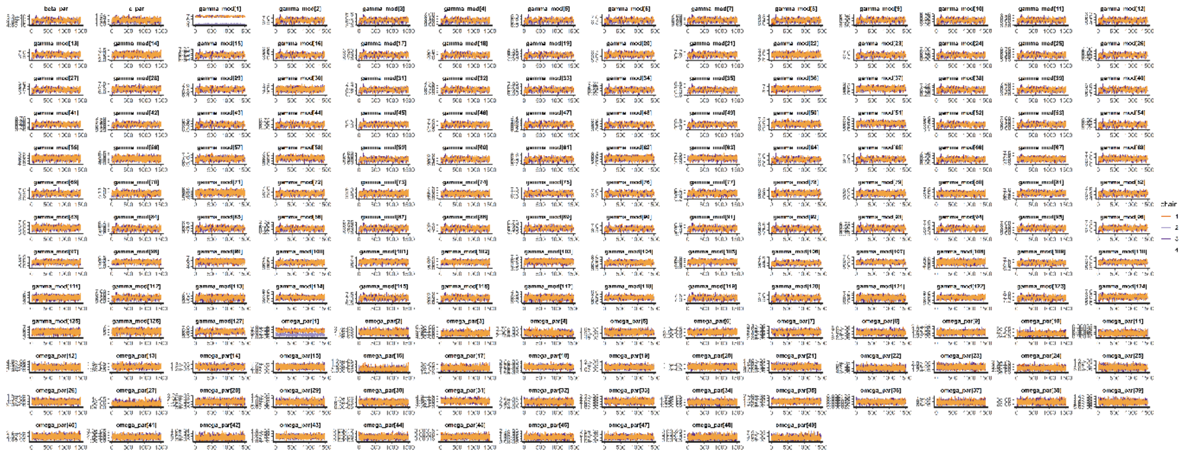

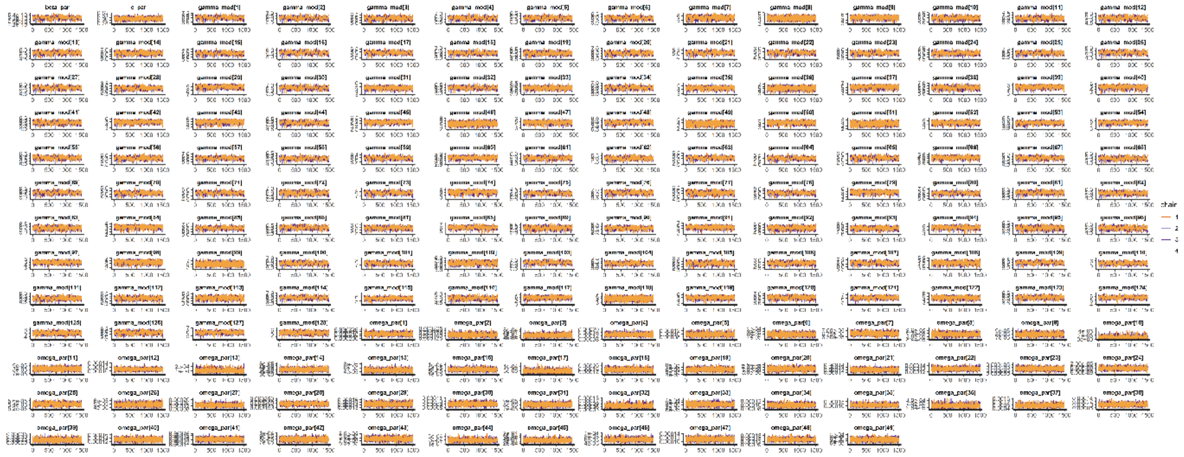

(3b) R-hat plot for Model Type 3. (Left) Delta infections, (Right) Omicron infections.

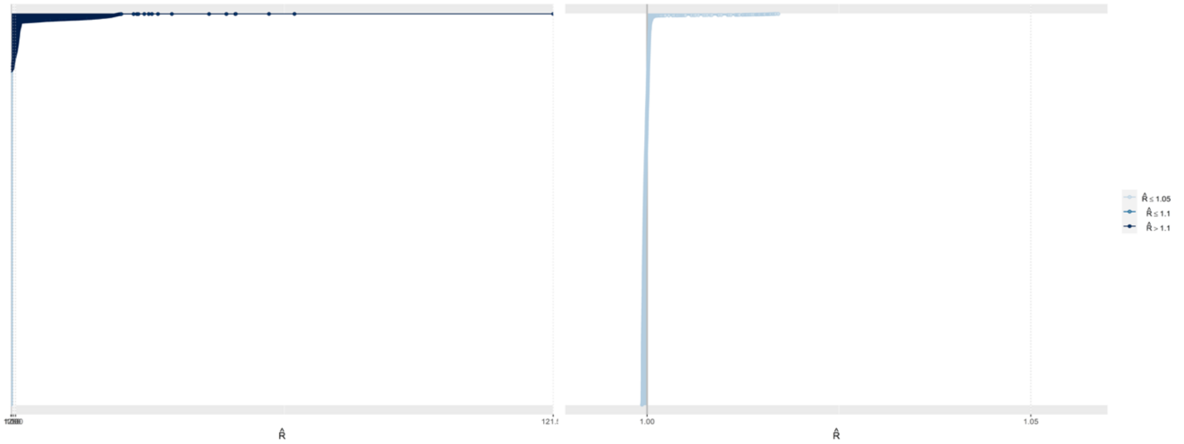

(4a) Trace plots for Model Type 4. (Top) Delta infections, (Bottom) Omicron infections.

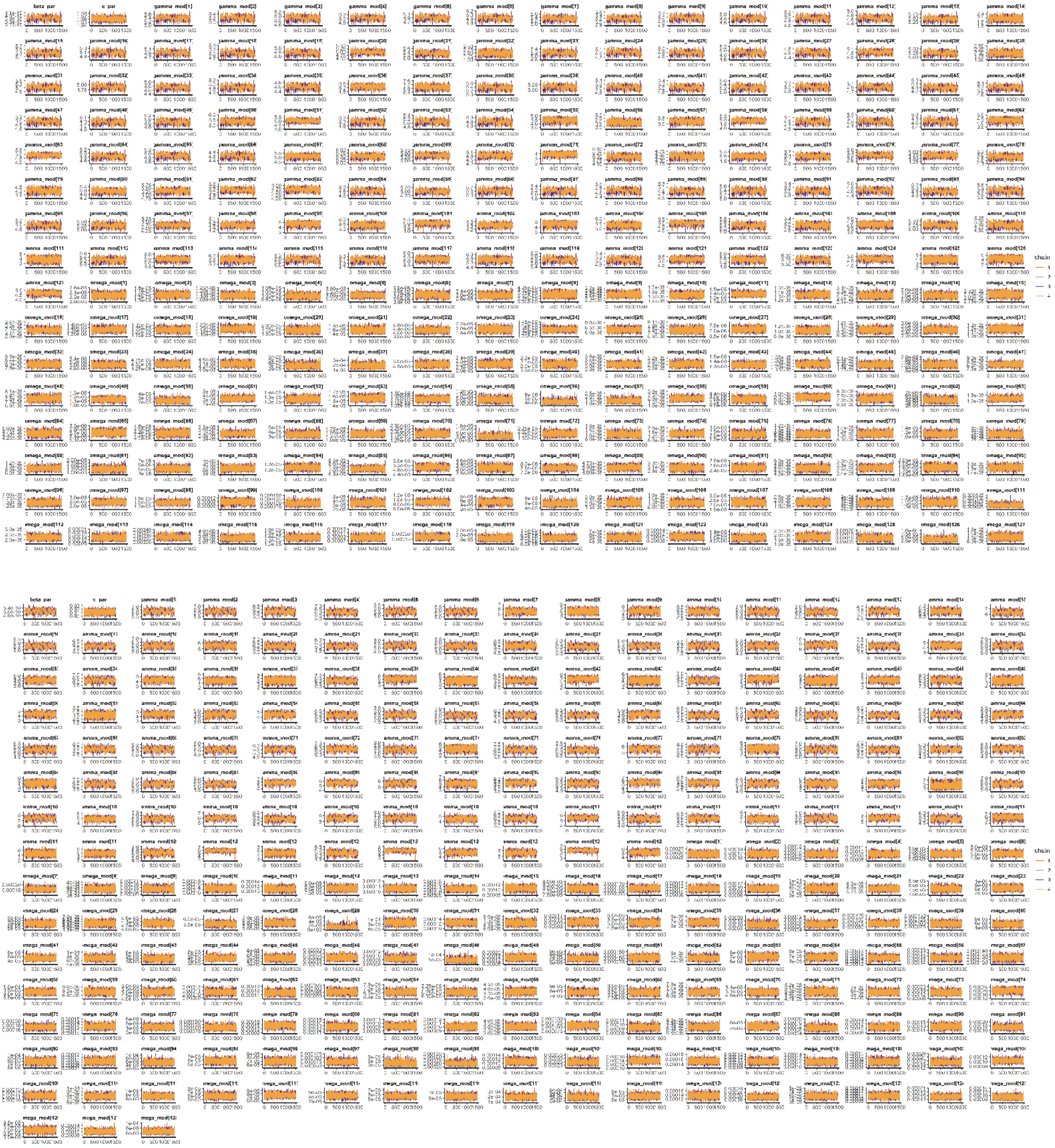

(4b) R-hat plot for Model Type 4. (Left) Delta infections, (Right) Omicron infections.

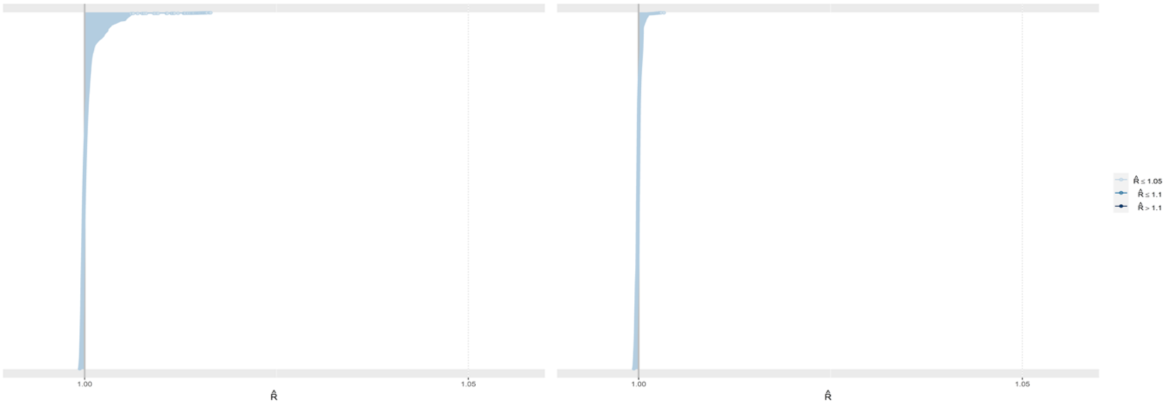

Fig S4. Posterior distributions of age modifier values (θ) for infected cell clearance by immune response parameter (γ) in Model Type 4 with medians and 95% intervals. Theta_g[1] is fixed at value of 1.0 for our chosen reference group of 18- 39 years. Theta_g[2] is the age modifier value for 0-4 years, theta_g[3] for 5-11 years, theta_g[4] for 12-17 years, theta_g[5] for 40-60 years, and theta_g[6] for 60+ years. (a) Delta dataset, (b) Omicron dataset.

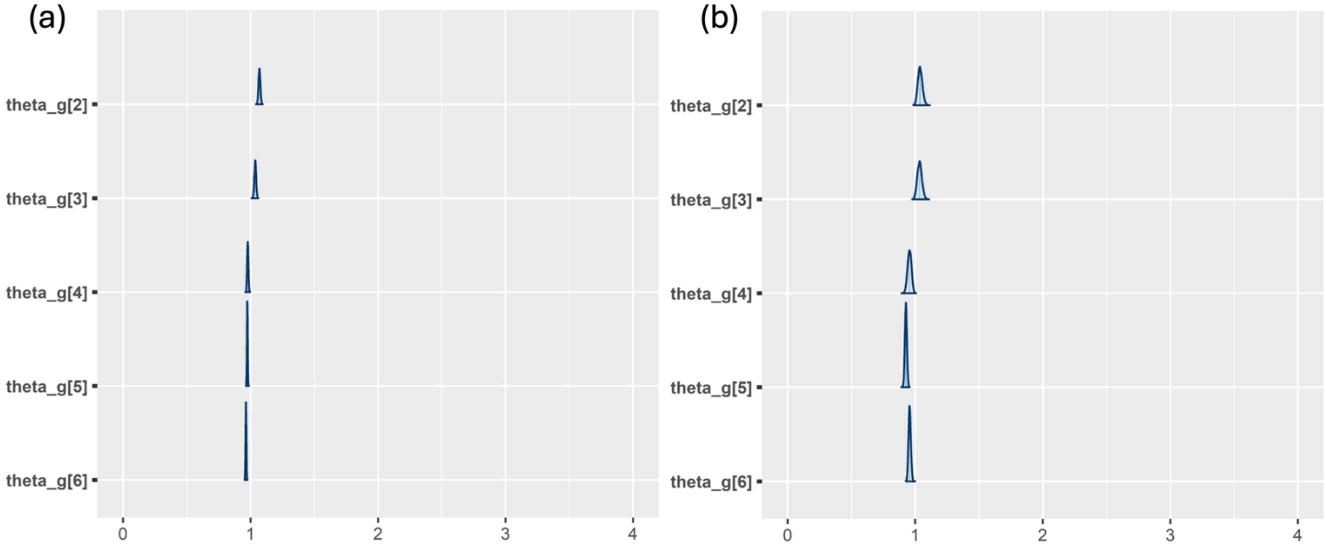

Fig S5. Posterior distributions of age modifier values (θ) for growth rate of immunity parameter (ω) in Model Type 2 with medians and 95% intervals. Theta[1] is fixed at value of 1.0 for our chosen reference group of 18- 39 years. Theta[2] is the age modifier value for 0-4 years, theta[3] for 5-11 years, theta [4] for 12-17 years, theta[5] for 40-60 years, and theta[6] for 60+ years. (a) Delta dataset, (b) Omicron dataset.

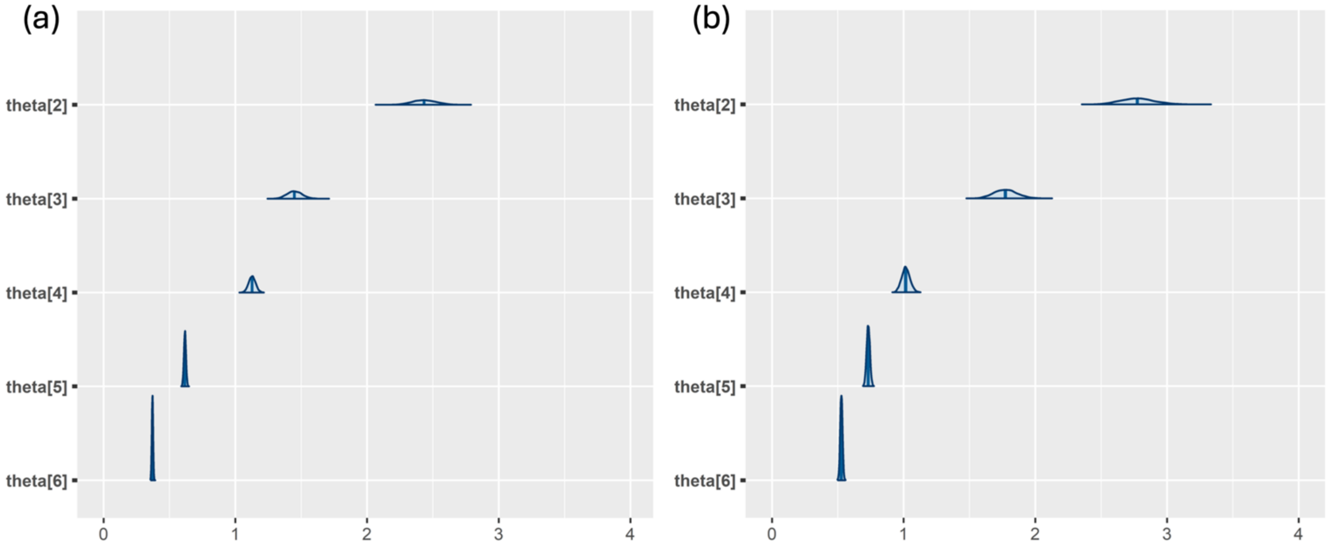

Equations S1

Model in which the main mechanism immunity controls infection is via clearance of free virus. In this model, free virus is cleared by immunity via a mass action process, and is represented by γ parameter. For this model, we estimated infection rate of target cells (β), natural clearance of infected cells (δ), rate of clearance of free virus by immunity (γ) and growth rate of immunity (ω). The natural clearance of virus (c) was fixed at 1.0 /day for this model.

$$\frac{dT}{dt} = A - \alpha T - \beta VT$$
$$\frac{dI}{dt} = \beta VT - \delta I$$
$$\frac{dV}{dt} = pI - cV - \gamma VZ$$
$$\frac{dZ}{dt} = \omega IZ$$

Fig S6. Observed viral load and output from Model Type 2 fit, the selected model which fits infected cell clearance rate by immunity (γ) as vaccination history-specific, and growth rate of immunity (ω) as age-modified, vaccination history-specific. Grey dots represent observed viral loads. Black lines are median posterior for virus dynamics. Green lines are samples from the posterior of uninfected target cell dynamics. Purple lines are samples from the posterior of immune response dynamics. Red lines are samples from the posterior of virus dynamics. (a) Delta infections, (b) Omicron infections.

(a) Delta

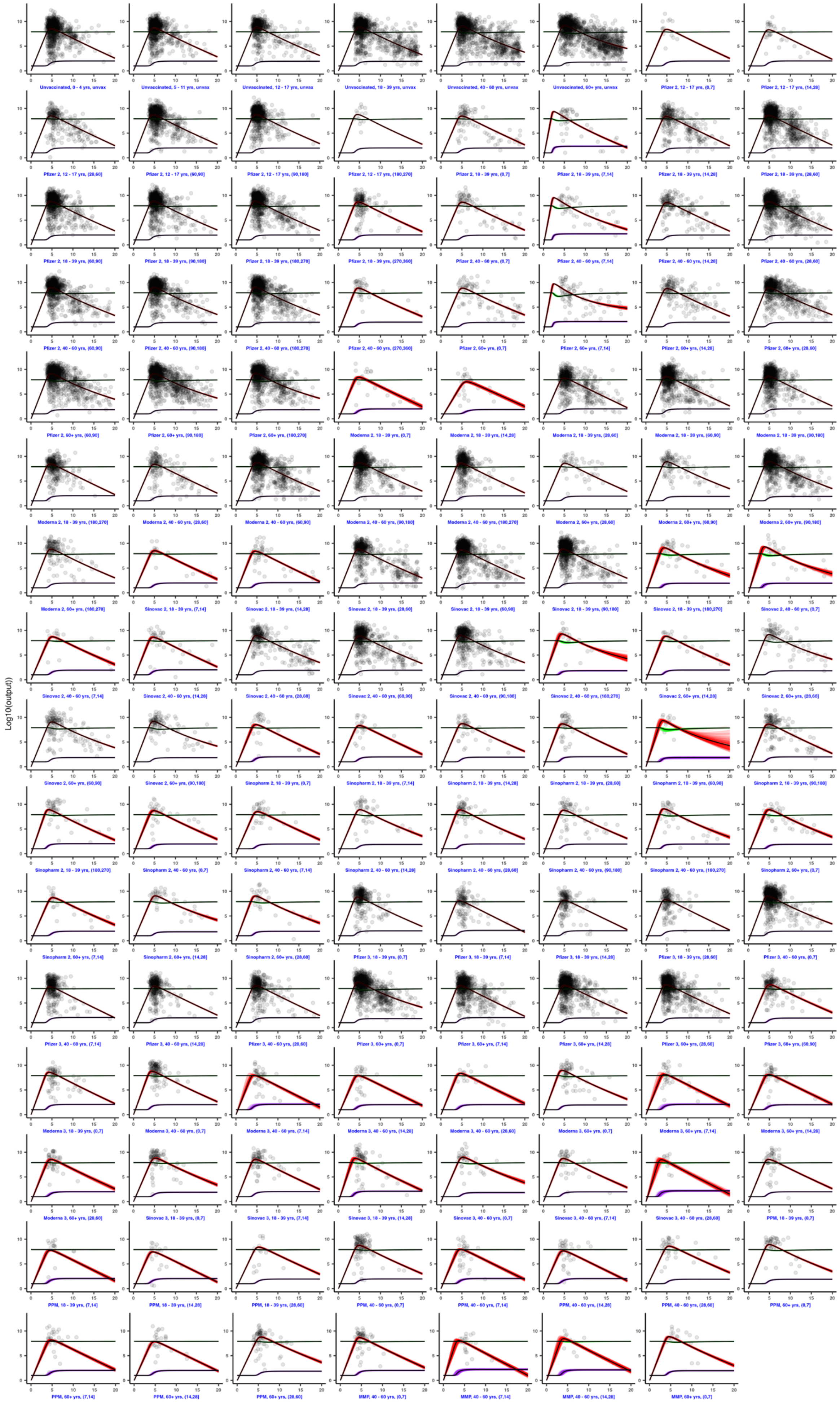
Log10(output))
Unvaccinated, 0 - 4 yrs, unvax
Unvaccinated, 5 - 11 yrs, unvax
Unvaccinated, 12 - 17 yrs, unvax
Unvaccinated, 18 - 39 yrs, unvax
Unvaccinated, 40 - 60 yrs, unvax
Unvaccinated, 60+ yrs, unvax
Pfizer 2, 12 - 17 yrs, (0,7]
Pfizer 2, 12 - 17 yrs, (14,28]
Pfizer 2, 12 - 17 yrs, (28,60]
Pfizer 2, 12 - 17 yrs, (60,90]
Pfizer 2, 12 - 17 yrs, (90,180]
Pfizer 2, 12 - 17 yrs, (180,270]
Pfizer 2, 18 - 39 yrs, (0,7]
Pfizer 2, 18 - 39 yrs, (7,14]
Pfizer 2, 18 - 39 yrs, (14,28]
Pfizer 2, 18 - 39 yrs, (28,60]
Pfizer 2, 18 - 39 yrs, (60,90]
Pfizer 2, 18 - 39 yrs, (90,180]
Pfizer 2, 18 - 39 yrs, (180,270]
Pfizer 2, 18 - 39 yrs, (270,360]
Pfizer 2, 40 - 60 yrs, (0,7]
Pfizer 2, 40 - 60 yrs, (7,14]
Pfizer 2, 40 - 60 yrs, (14,28]
Pfizer 2, 40 - 60 yrs, (28,60]
Pfizer 2, 40 - 60 yrs, (60,90]
Pfizer 2, 40 - 60 yrs, (90,180]
Pfizer 2, 40 - 60 yrs, (180,270]
Pfizer 2, 40 - 60 yrs, (270,360]
Pfizer 2, 60+ yrs, (0,7]
Pfizer 2, 60+ yrs, (7,14]
Pfizer 2, 60+ yrs, (14,28]
Pfizer 2, 60+ yrs, (28,60]
Pfizer 2, 60+ yrs, (60,90]
Pfizer 2, 60+ yrs, (90,180]
Pfizer 2, 60+ yrs, (180,270]
Moderna 2, 18 - 39 yrs, (0,7]
Moderna 2, 18 - 39 yrs, (14,28]
Moderna 2, 18 - 39 yrs, (28,60]
Moderna 2, 18 - 39 yrs, (60,90]
Moderna 2, 18 - 39 yrs, (90,180]
Moderna 2, 18 - 39 yrs, (180,270]
Moderna 2, 40 - 60 yrs, (28,60]
Moderna 2, 40 - 60 yrs, (60,90]
Moderna 2, 40 - 60 yrs, (90,180]
Moderna 2, 40 - 60 yrs, (180,270]
Moderna 2, 60+ yrs, (28,60]
Moderna 2, 60+ yrs, (60,90]
Moderna 2, 60+ yrs, (90,180]
Moderna 2, 60+ yrs, (180,270]
Sinovac 2, 18 - 39 yrs, (7,14]
Sinovac 2, 18 - 39 yrs, (14,28]
Sinovac 2, 18 - 39 yrs, (28,60]
Sinovac 2, 18 - 39 yrs, (60,90]
Sinovac 2, 18 - 39 yrs, (90,180]
Sinovac 2, 18 - 39 yrs, (180,270]
Sinovac 2, 40 - 60 yrs, (0,7]
Sinovac 2, 40 - 60 yrs, (7,14]
Sinovac 2, 40 - 60 yrs, (14,28]
Sinovac 2, 40 - 60 yrs, (28,60]
Sinovac 2, 40 - 60 yrs, (60,90]
Sinovac 2, 40 - 60 yrs, (90,180]
Sinovac 2, 40 - 60 yrs, (180,270]
Sinovac 2, 60+ yrs, (14,28]
Sinovac 2, 60+ yrs, (28,60]
Sinovac 2, 60+ yrs, (60,90]
Sinovac 2, 60+ yrs, (90,180]
Sinopharm 2, 18 - 39 yrs, (0,7]
Sinopharm 2, 18 - 39 yrs, (7,14]
Sinopharm 2, 18 - 39 yrs, (14,28]
Sinopharm 2, 18 - 39 yrs, (28,60]
Sinopharm 2, 18 - 39 yrs, (60,90]
Sinopharm 2, 18 - 39 yrs, (90,180]
Sinopharm 2, 18 - 39 yrs, (180,270]
Sinopharm 2, 40 - 60 yrs, (0,7]
Sinopharm 2, 40 - 60 yrs, (7,14]
Sinopharm 2, 40 - 60 yrs, (14,28]
Sinopharm 2, 40 - 60 yrs, (28,60]
Sinopharm 2, 40 - 60 yrs, (90,180]
Sinopharm 2, 40 - 60 yrs, (180,270]
Sinopharm 2, 60+ yrs, (0,7]
Sinopharm 2, 60+ yrs, (7,14]
Sinopharm 2, 60+ yrs, (14,28]
Sinopharm 2, 60+ yrs, (28,60]
Pfizer 3, 18 - 39 yrs, (0,7]
Pfizer 3, 18 - 39 yrs, (7,14]
Pfizer 3, 18 - 39 yrs, (14,28]
Pfizer 3, 18 - 39 yrs, (28,60]
Pfizer 3, 40 - 60 yrs, (0,7]
Pfizer 3, 40 - 60 yrs, (7,14]
Pfizer 3, 40 - 60 yrs, (14,28]
Pfizer 3, 40 - 60 yrs, (28,60]
Pfizer 3, 60+ yrs, (0,7]
Pfizer 3, 60+ yrs, (7,14]
Pfizer 3, 60+ yrs, (14,28]
Pfizer 3, 60+ yrs, (28,60]
Pfizer 3, 60+ yrs, (60,90]
Moderna 3, 18 - 39 yrs, (0,7]
Moderna 3, 40 - 60 yrs, (0,7]
Moderna 3, 40 - 60 yrs, (7,14]
Moderna 3, 40 - 60 yrs, (14,28]
Moderna 3, 40 - 60 yrs, (28,60]
Moderna 3, 60+ yrs, (0,7]
Moderna 3, 60+ yrs, (7,14]
Moderna 3, 60+ yrs, (14,28]
Moderna 3, 60+ yrs, (28,60]
Sinovac 3, 18 - 39 yrs, (0,7]
Sinovac 3, 18 - 39 yrs, (7,14]
Sinovac 3, 18 - 39 yrs, (14,28]
Sinovac 3, 40 - 60 yrs, (0,7]
Sinovac 3, 40 - 60 yrs, (7,14]
Sinovac 3, 40 - 60 yrs, (28,60]
PPM, 18 - 39 yrs, (0,7]
PPM, 18 - 39 yrs, (7,14]
PPM, 18 - 39 yrs, (14,28]
PPM, 18 - 39 yrs, (28,60]
PPM, 40 - 60 yrs, (0,7]
PPM, 40 - 60 yrs, (7,14]
PPM, 40 - 60 yrs, (14,28]
PPM, 40 - 60 yrs, (28,60]
PPM, 60+ yrs, (0,7]
PPM, 60+ yrs, (7,14]
PPM, 60+ yrs, (14,28]
PPM, 60+ yrs, (28,60]
MMP, 40 - 60 yrs, (0,7]
MMP, 40 - 60 yrs, (7,14]
MMP, 40 - 60 yrs, (14,28]
MMP, 60+ yrs, (0,7]

(b) Omicron

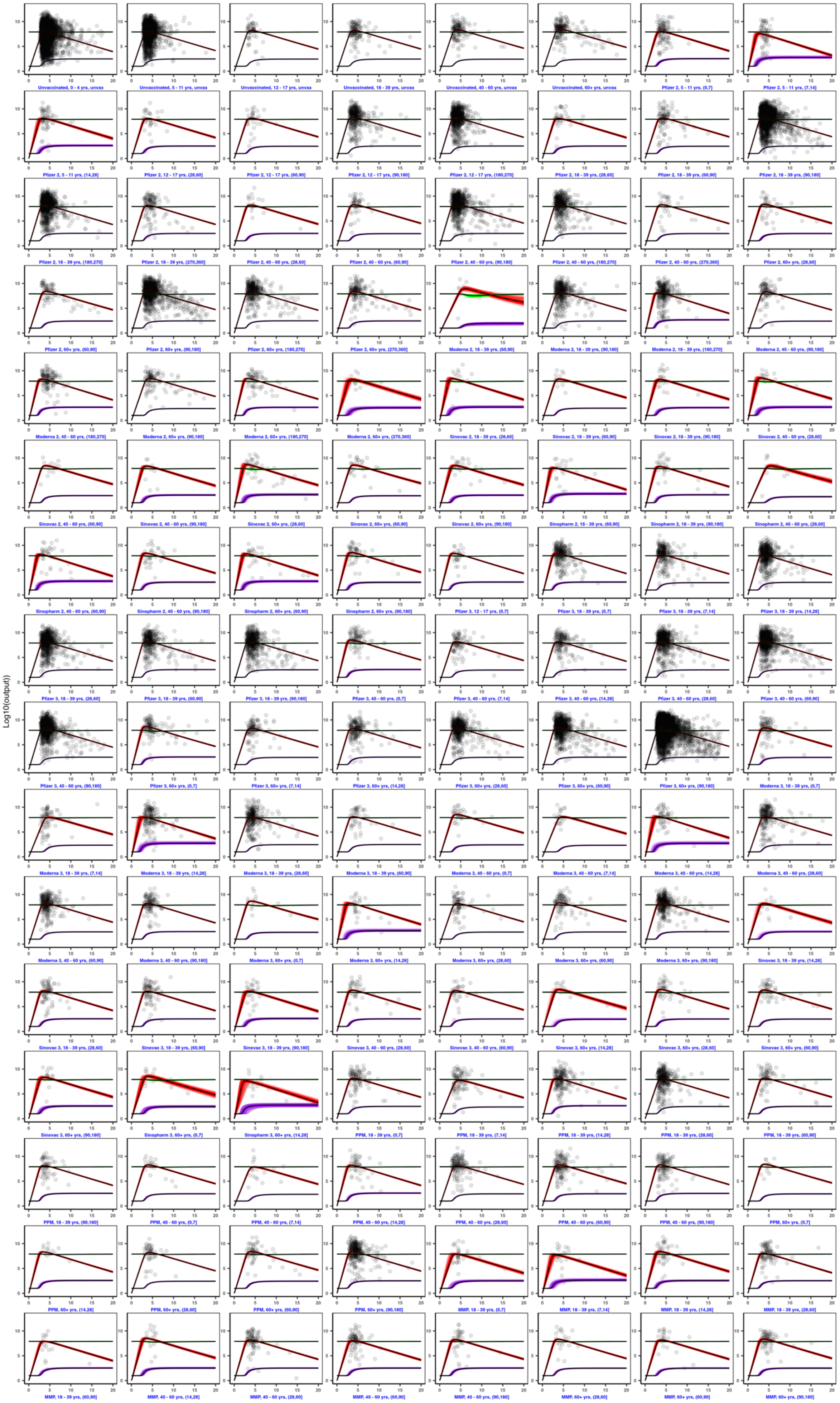

Log10(output))
Unvaccinated, 0 - 4 yrs, unvax
Unvaccinated, 5 - 11 yrs, unvax
Unvaccinated, 12 - 17 yrs, unvax
Unvaccinated, 18 - 39 yrs, unvax
Unvaccinated, 40 - 60 yrs, unvax
Unvaccinated, 60+ yrs, unvax
Pfizer 2, 5 - 11 yrs, (0,7]
Pfizer 2, 5 - 11 yrs, (7,14]
Pfizer 2, 5 - 11 yrs, (14,28]
Pfizer 2, 12 - 17 yrs, (28,60]
Pfizer 2, 12 - 17 yrs, (60,90]
Pfizer 2, 12 - 17 yrs, (90,180]
Pfizer 2, 12 - 17 yrs, (180,270]
Pfizer 2, 18 - 39 yrs, (28,60]
Pfizer 2, 18 - 39 yrs, (60,90]
Pfizer 2, 18 - 39 yrs, (90,180]
Pfizer 2, 18 - 39 yrs, (180,270]
Pfizer 2, 18 - 39 yrs, (270,360]
Pfizer 2, 40 - 60 yrs, (28,60]
Pfizer 2, 40 - 60 yrs, (60,90]
Pfizer 2, 40 - 60 yrs, (90,180]
Pfizer 2, 40 - 60 yrs, (180,270]
Pfizer 2, 40 - 60 yrs, (270,360]
Pfizer 2, 60+ yrs, (28,60]
Pfizer 2, 60+ yrs, (60,90]
Pfizer 2, 60+ yrs, (90,180]
Pfizer 2, 60+ yrs, (180,270]
Pfizer 2, 60+ yrs, (270,360]
Moderna 2, 18 - 39 yrs, (60,90]
Moderna 2, 18 - 39 yrs, (90,180]
Moderna 2, 18 - 39 yrs, (180,270]
Moderna 2, 40 - 60 yrs, (90,180]
Moderna 2, 40 - 60 yrs, (180,270]
Moderna 2, 60+ yrs, (90,180]
Moderna 2, 60+ yrs, (180,270]
Moderna 2, 60+ yrs, (270,360]
Sinovac 2, 18 - 39 yrs, (28,60]
Sinovac 2, 18 - 39 yrs, (60,90]
Sinovac 2, 18 - 39 yrs, (90,180]
Sinovac 2, 40 - 60 yrs, (28,60]
Sinovac 2, 40 - 60 yrs, (60,90]
Sinovac 2, 40 - 60 yrs, (90,180]
Sinovac 2, 60+ yrs, (28,60]
Sinovac 2, 60+ yrs, (60,90]
Sinovac 2, 60+ yrs, (90,180]
Sinopharm 2, 18 - 39 yrs, (60,90]
Sinopharm 2, 18 - 39 yrs, (90,180]
Sinopharm 2, 40 - 60 yrs, (28,60]
Sinopharm 2, 40 - 60 yrs, (60,90]
Sinopharm 2, 40 - 60 yrs, (90,180]
Sinopharm 2, 60+ yrs, (60,90]
Sinopharm 2, 60+ yrs, (90,180]
Pfizer 3, 12 - 17 yrs, (0,7]
Pfizer 3, 18 - 39 yrs, (0,7]
Pfizer 3, 18 - 39 yrs, (7,14]
Pfizer 3, 18 - 39 yrs, (14,28]
Pfizer 3, 18 - 39 yrs, (28,60]
Pfizer 3, 18 - 39 yrs, (60,90]
Pfizer 3, 18 - 39 yrs, (90,180]
Pfizer 3, 40 - 60 yrs, (0,7]
Pfizer 3, 40 - 60 yrs, (7,14]
Pfizer 3, 40 - 60 yrs, (14,28]
Pfizer 3, 40 - 60 yrs, (28,60]
Pfizer 3, 40 - 60 yrs, (60,90]
Pfizer 3, 40 - 60 yrs, (90,180]
Pfizer 3, 60+ yrs, (0,7]
Pfizer 3, 60+ yrs, (7,14]
Pfizer 3, 60+ yrs, (14,28]
Pfizer 3, 60+ yrs, (28,60]
Pfizer 3, 60+ yrs, (60,90]
Pfizer 3, 60+ yrs, (90,180]
Moderna 3, 18 - 39 yrs, (0,7]
Moderna 3, 18 - 39 yrs, (7,14]
Moderna 3, 18 - 39 yrs, (14,28]
Moderna 3, 18 - 39 yrs, (28,60]
Moderna 3, 18 - 39 yrs, (60,90]
Moderna 3, 40 - 60 yrs, (0,7]
Moderna 3, 40 - 60 yrs, (7,14]
Moderna 3, 40 - 60 yrs, (14,28]
Moderna 3, 40 - 60 yrs, (28,60]
Moderna 3, 40 - 60 yrs, (60,90]
Moderna 3, 40 - 60 yrs, (90,180]
Moderna 3, 60+ yrs, (0,7]
Moderna 3, 60+ yrs, (14,28]
Moderna 3, 60+ yrs, (28,60]
Moderna 3, 60+ yrs, (60,90]
Moderna 3, 60+ yrs, (90,180]
Sinovac 3, 18 - 39 yrs, (14,28]
Sinovac 3, 18 - 39 yrs, (28,60]
Sinovac 3, 18 - 39 yrs, (60,90]
Sinovac 3, 18 - 39 yrs, (90,180]
Sinovac 3, 40 - 60 yrs, (28,60]
Sinovac 3, 40 - 60 yrs, (60,90]
Sinovac 3, 60+ yrs, (14,28]
Sinovac 3, 60+ yrs, (28,60]
Sinovac 3, 60+ yrs, (60,90]
Sinovac 3, 60+ yrs, (90,180]
Sinopharm 3, 60+ yrs, (0,7]
Sinopharm 3, 60+ yrs, (14,28]
PPM, 18 - 39 yrs, (0,7]
PPM, 18 - 39 yrs, (7,14]
PPM, 18 - 39 yrs, (14,28]
PPM, 18 - 39 yrs, (28,60]
PPM, 18 - 39 yrs, (60,90]
PPM, 18 - 39 yrs, (90,180]
PPM, 40 - 60 yrs, (0,7]
PPM, 40 - 60 yrs, (7,14]
PPM, 40 - 60 yrs, (14,28]
PPM, 40 - 60 yrs, (28,60]
PPM, 40 - 60 yrs, (60,90]
PPM, 40 - 60 yrs, (90,180]
PPM, 60+ yrs, (0,7]
PPM, 60+ yrs, (14,28]
PPM, 60+ yrs, (28,60]
PPM, 60+ yrs, (60,90]
PPM, 60+ yrs, (90,180]
MMP, 18 - 39 yrs, (0,7]
MMP, 18 - 39 yrs, (7,14]
MMP, 18 - 39 yrs, (14,28]
MMP, 18 - 39 yrs, (28,60]
MMP, 18 - 39 yrs, (60,90]
MMP, 40 - 60 yrs, (14,28]
MMP, 40 - 60 yrs, (28,60]
MMP, 40 - 60 yrs, (60,90]
MMP, 40 - 60 yrs, (90,180]
MMP, 60+ yrs, (28,60]
MMP, 60+ yrs, (60,90]
MMP, 60+ yrs, (90,180]

Fig S7. Viral load outputs from model fit to dataset which only includes the first swab taken per patient. Viral loads are stratified by age group, vaccine brand and number of doses received. mRNA-based vaccines are represented by the following colour gradients; yellow to orange for Pfizer-only groups, light green to emerald for Moderna-based groups, lilac to dark purple for PPM, and light blue to navy for MMP. Non-mRNA-based vaccines are represented as follows; light blue to navy for Sinopharm groups, and pink to maroon for Sinovac. Colour gradient scales with increasing time since last vaccinated. Unvaccinated groups are shown in black, and plotted as a reference. (a) Delta infections (b) Omicron infections.

(a) Delta

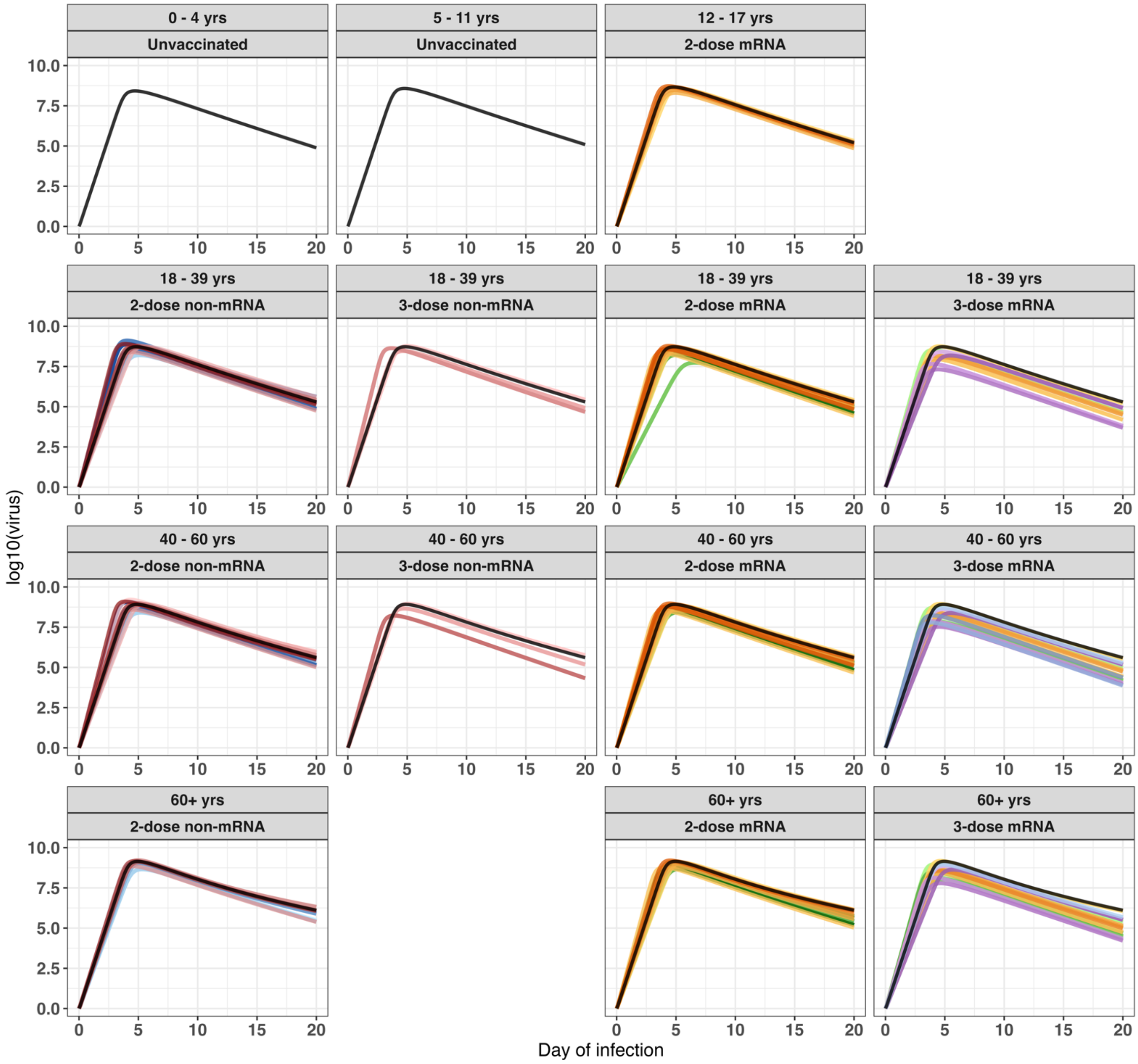

(b) Omicron

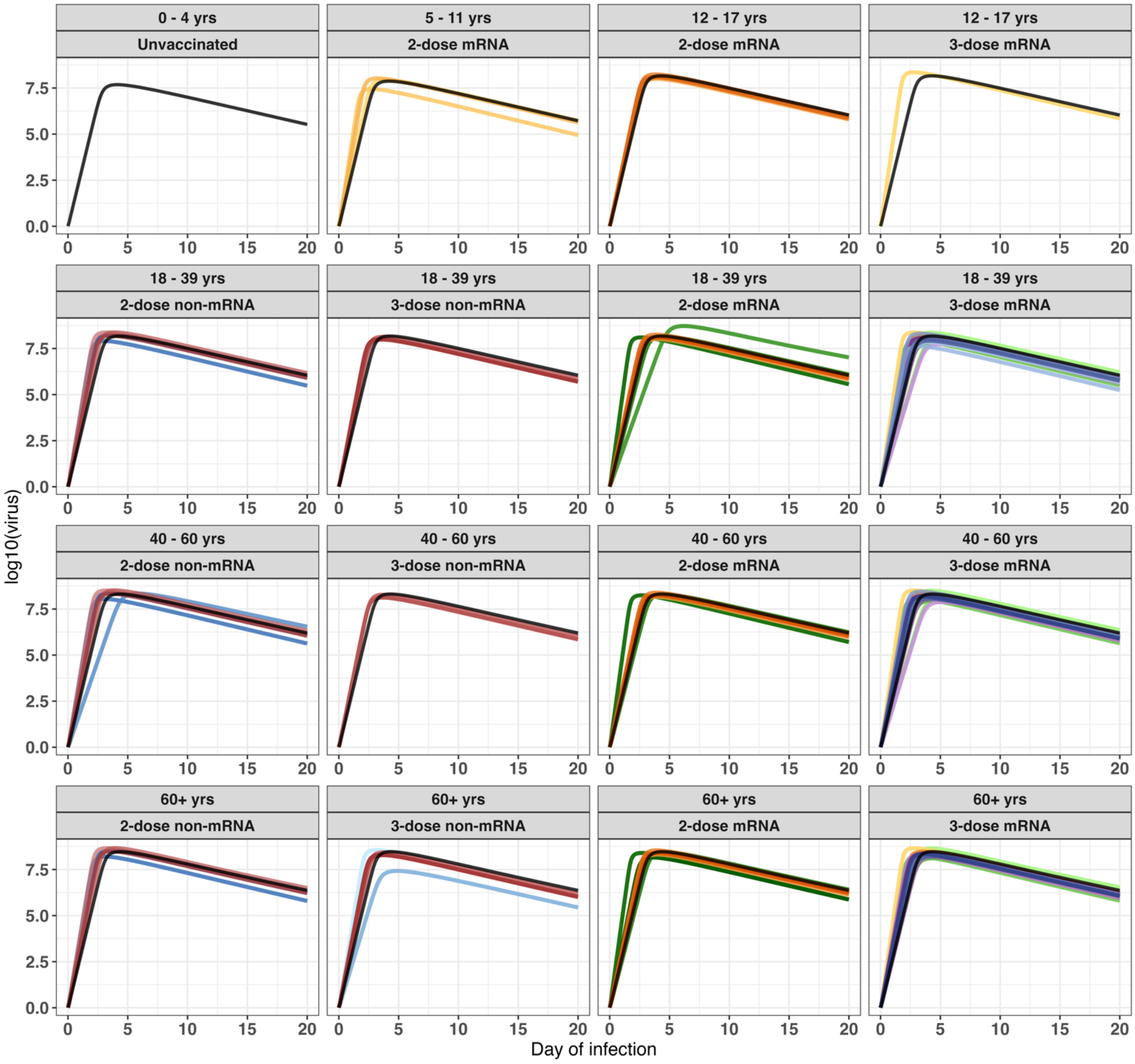
0 - 4 yrs
Unvaccinated
5 - 11 yrs
2-dose mRNA
12 - 17 yrs
2-dose mRNA
12 - 17 yrs
3-dose mRNA
18 - 39 yrs
2-dose non-mRNA
18 - 39 yrs
3-dose non-mRNA
18 - 39 yrs
2-dose mRNA
18 - 39 yrs
3-dose mRNA
40 - 60 yrs
2-dose non-mRNA
40 - 60 yrs
3-dose non-mRNA
40 - 60 yrs
2-dose mRNA
40 - 60 yrs
3-dose mRNA
60+ yrs
2-dose non-mRNA
60+ yrs
3-dose non-mRNA
60+ yrs
2-dose mRNA
60+ yrs
3-dose mRNA
log10(virus)
0.0
2.5
5.0
7.5
0
5
10
15
20
Day of infection

Fig S8. Viral load outputs from model fit to dataset which only includes patients with one swab taken. Viral loads are stratified by age group, vaccine brand and number of doses received. mRNA-based vaccines are represented by the following colour gradients; yellow to orange for Pfizer-only groups, light green to emerald for Moderna-based groups, lilac to dark purple for PPM, and light blue to navy for MMP. Non-mRNA-based vaccines are represented as follows; light blue to navy for Sinopharm groups, and pink to maroon for Sinovac. Colour gradient scales with increasing time since last vaccinated. Unvaccinated groups are shown in black, and plotted as a reference. (a) Delta infections (b) Omicron infections.

(a) Delta

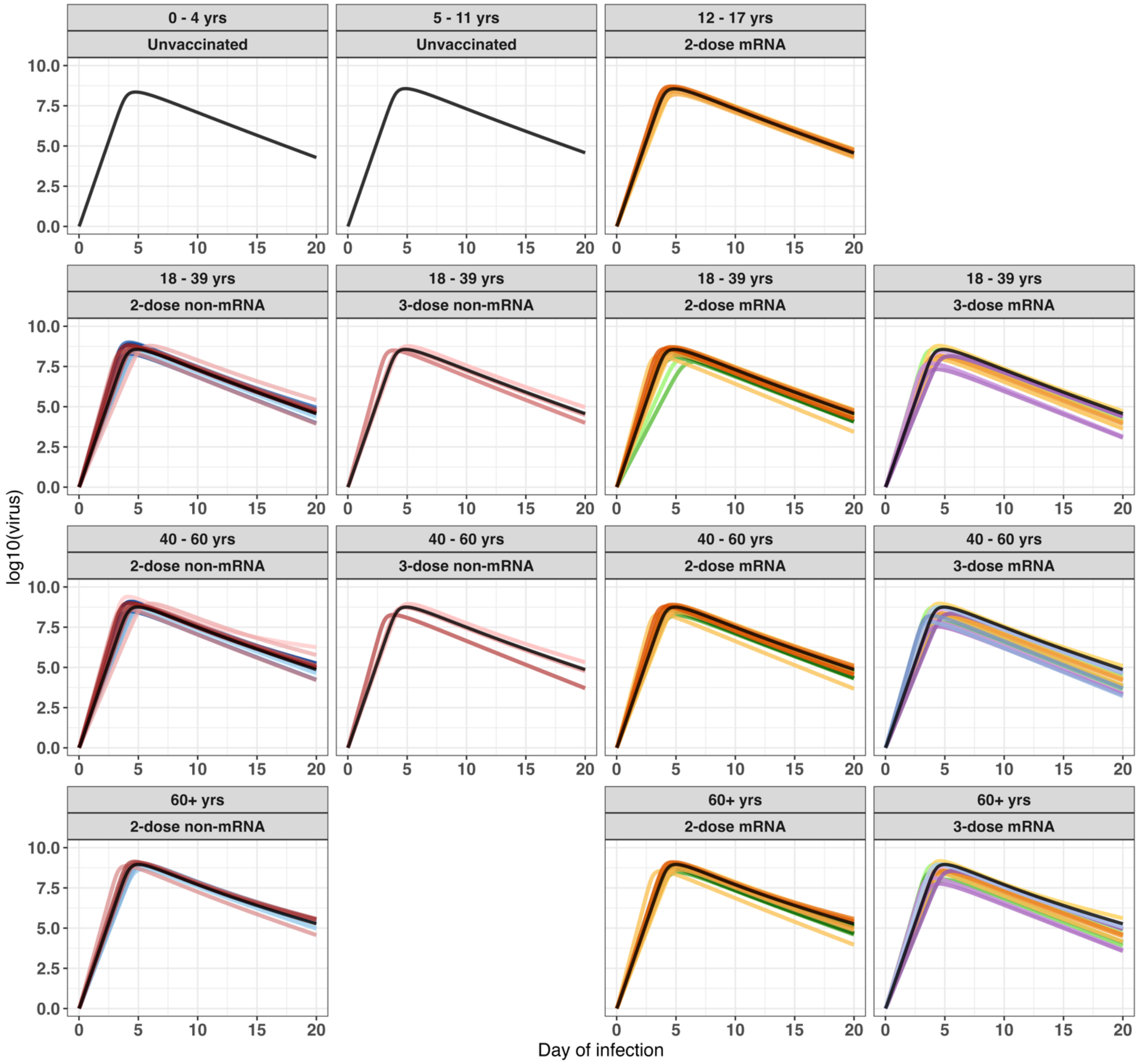

(b) Omicron

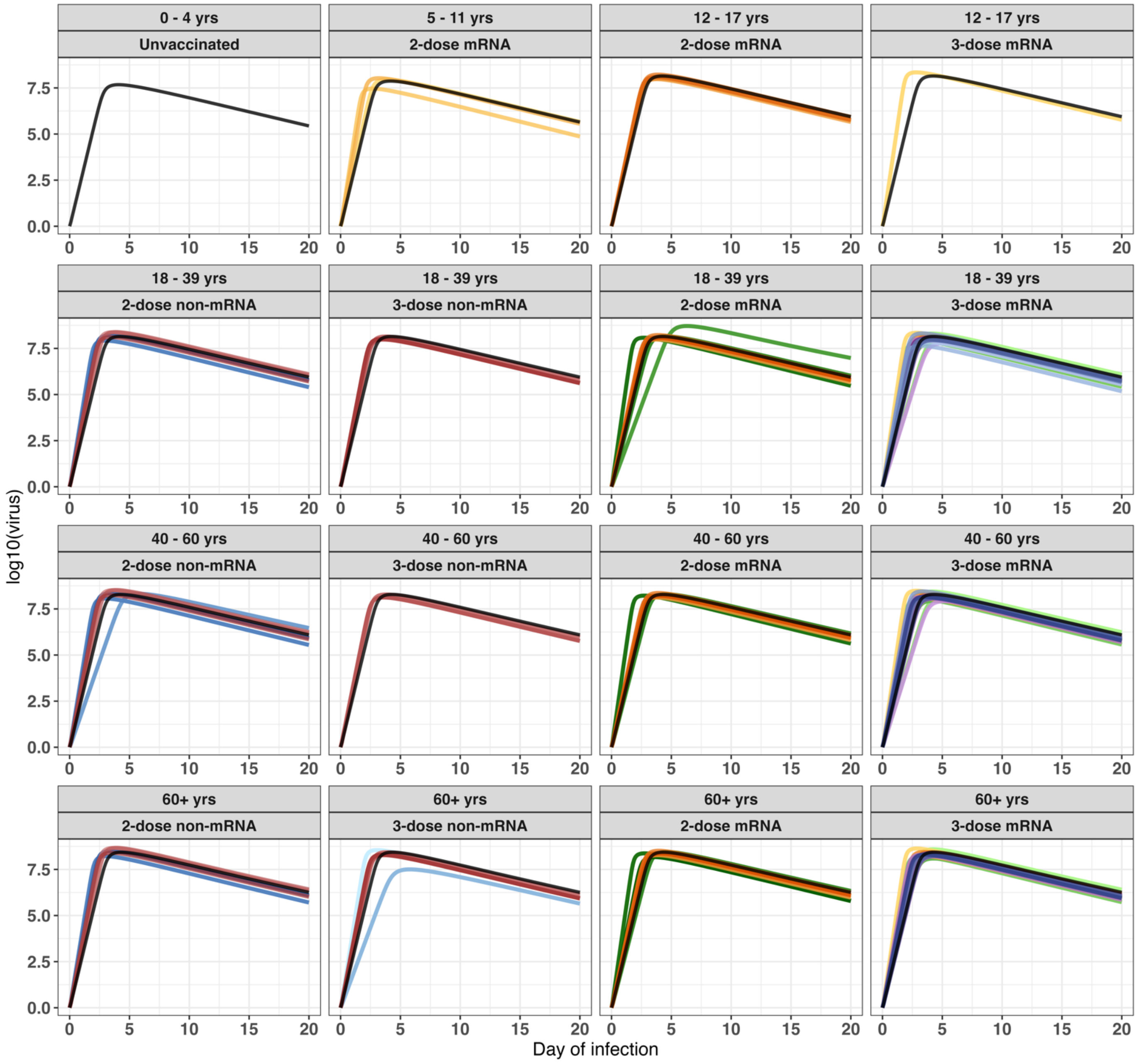
0 - 4 yrs
Unvaccinated
5 - 11 yrs
2-dose mRNA
12 - 17 yrs
2-dose mRNA
12 - 17 yrs
3-dose mRNA
18 - 39 yrs
2-dose non-mRNA
18 - 39 yrs
3-dose non-mRNA
18 - 39 yrs
2-dose mRNA
18 - 39 yrs
3-dose mRNA
40 - 60 yrs
2-dose non-mRNA
40 - 60 yrs
3-dose non-mRNA
40 - 60 yrs
2-dose mRNA
40 - 60 yrs
3-dose mRNA
60+ yrs
2-dose non-mRNA
60+ yrs
3-dose non-mRNA
60+ yrs
2-dose mRNA
60+ yrs
3-dose mRNA
log10(virus)
Day of infection
0.0
2.5
5.0
7.5
0
5
10
15
20

Fig S9. Viral load outputs from model fit to dataset with swapped incubation period distributions for Delta and Omicron infections. Viral loads are stratified by age group, vaccine brand and number of doses received. mRNA-based vaccines are represented by the following colour gradients; yellow to orange for Pfizer-only groups, light green to emerald for Moderna-based groups, lilac to dark purple for PPM, and light blue to navy for MMP. Non-mRNA-based vaccines are represented as follows; light blue to navy for Sinopharm groups, and pink to maroon for Sinovac. Colour gradient scales with increasing time since last vaccinated. Unvaccinated groups are shown in black, and plotted as a reference. (a) Delta infections (b) Omicron infections.

(a) Delta

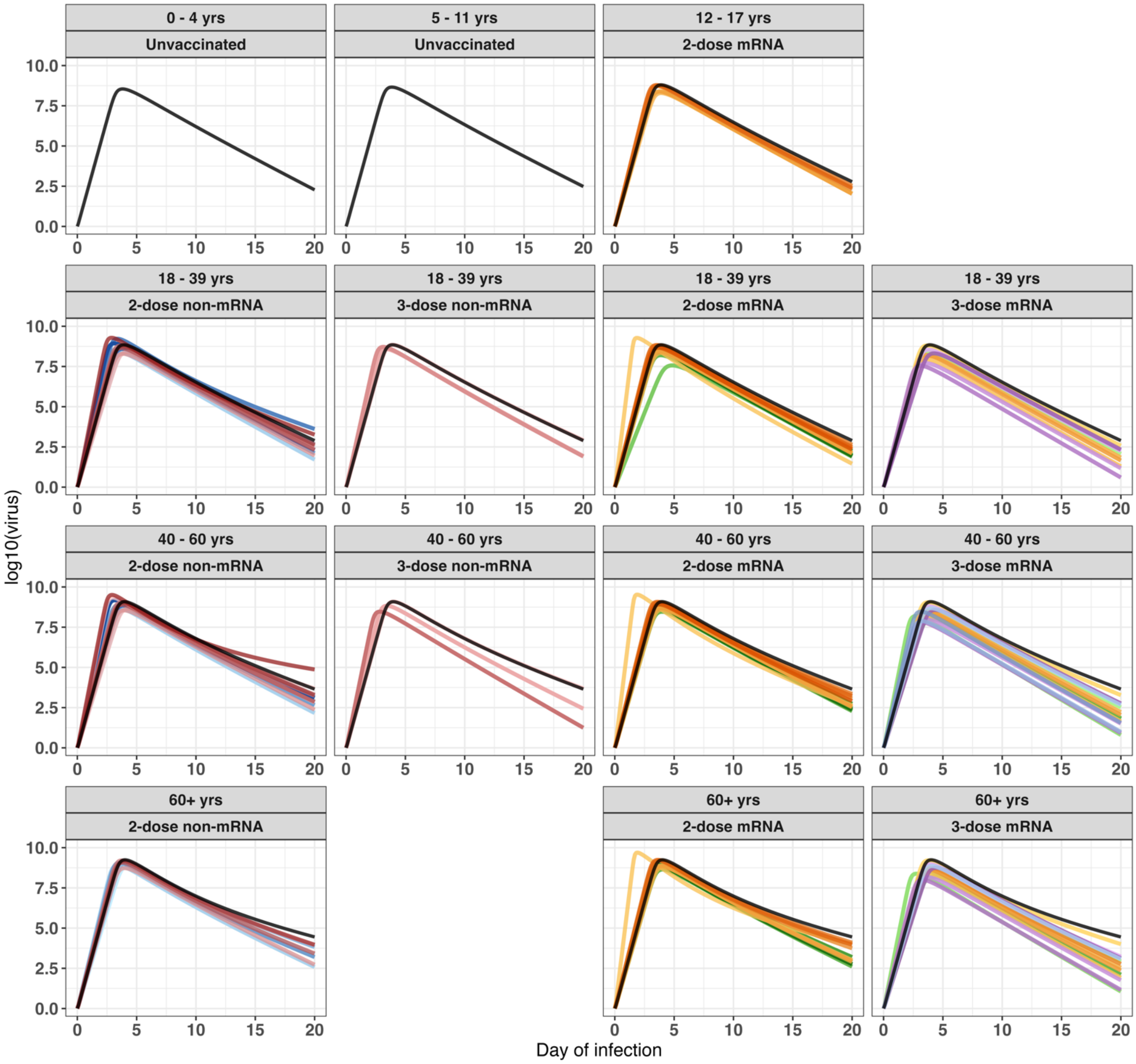

(b) Omicron

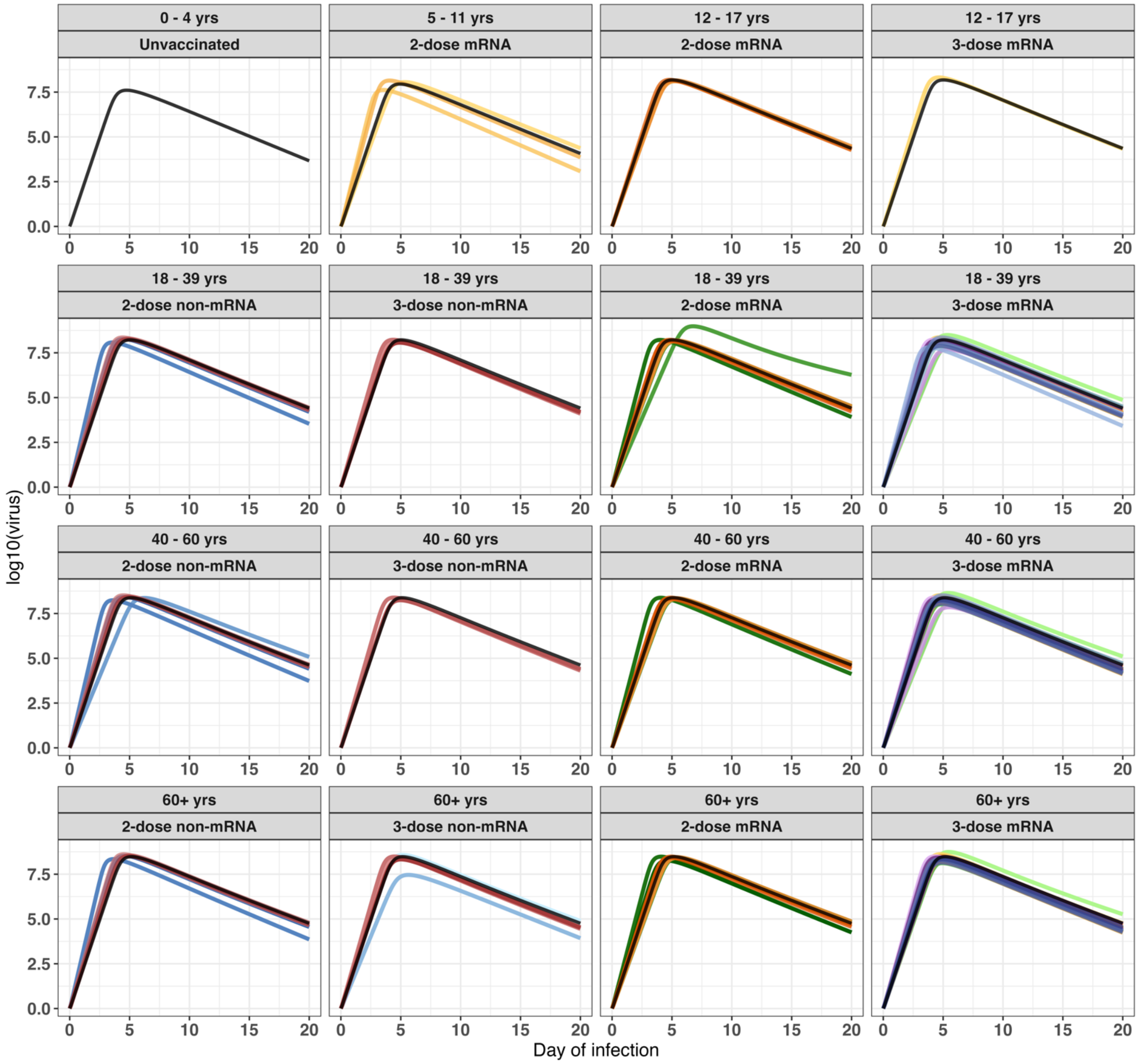

Fig S10

(a) Trace plots for virus neutralisation model (Top) Delta infections, (Bottom) Omicron infections.

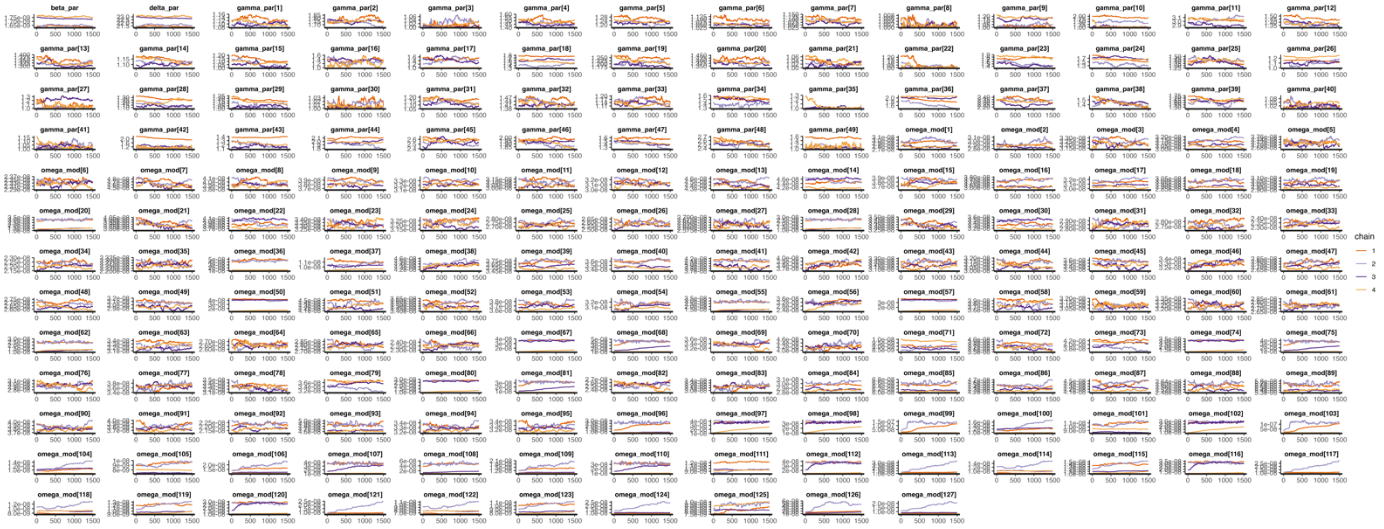

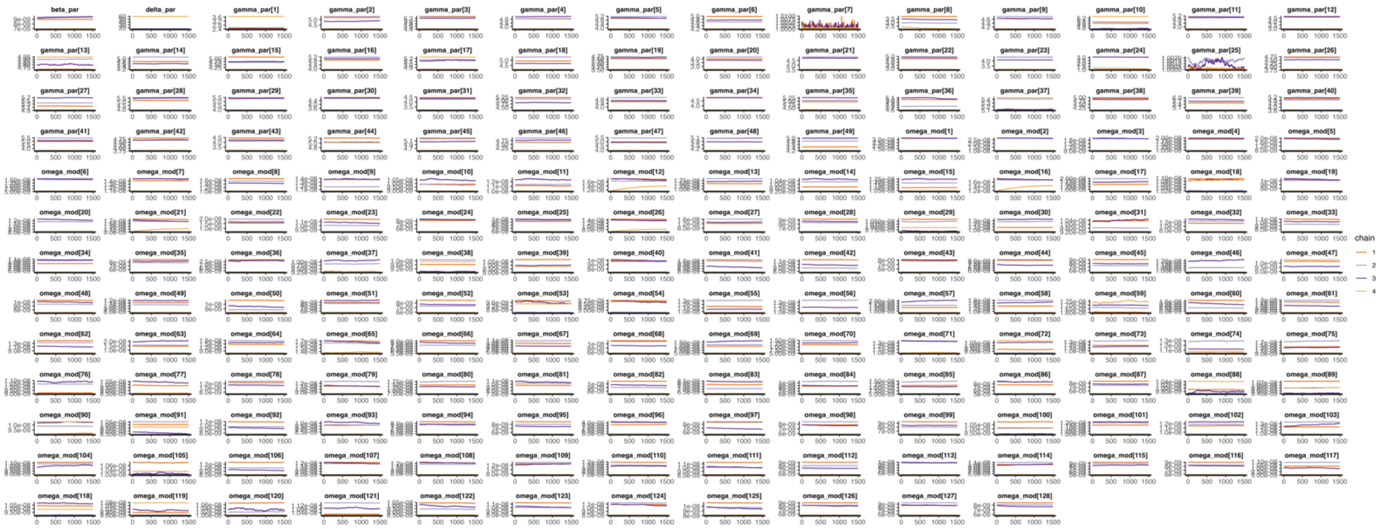

(b) R-hat plot for virus neutralisation model. (Left) Delta infections, (Right) Omicron infections.

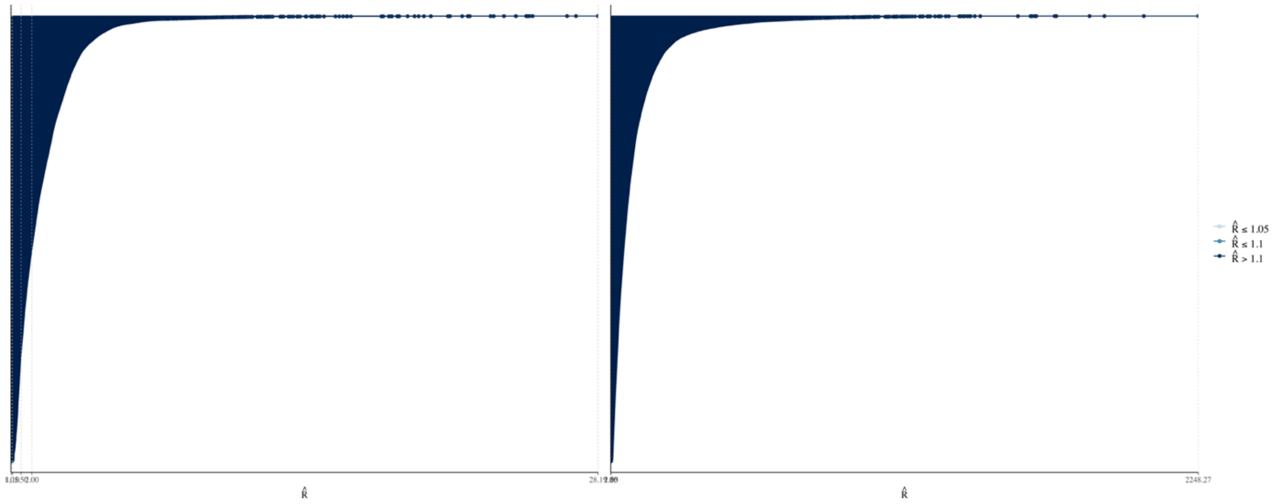

S1 Table. Summary of log10(viral load) decrease rate per day (R) and $\log_{10}$(viral load) before start of decrease (I) for each vaccination history and age subgroup for Delta and Omicron infections.
(See attached excel file)

S2 Table. Summary of the four model types considered, and the levels at which the four fitted parameters were estimated.

Model Type	1	2	3	4
VOC-specific parameter	β, c	β, c	β, c	β, c
Vaccination history-specific parameter	γ, ω	γ	ω	-
Age-modified, vaccination history-specific parameter		ω	γ	γ, ω

The parameters are infection rate of target cells (β), natural clearance rate of free virus (c), infected cell clearance by immune response (γ), and growth rate of immunity (ω) (for model equations, see Methods).

Fig S11. Observed viral load and output from model fit to dataset which only includes the first swab taken per patient for Delta and Omicron infections. Grey dots represent observed viral loads. Black lines are median posterior for virus dynamics. Green lines are samples from the posterior of uninfected target cell dynamics. Purple lines are samples from the posterior of immune response dynamics. Red lines are samples from the posterior of virus dynamics. (a) Delta infections, (b) Omicron infections.

(a) Delta

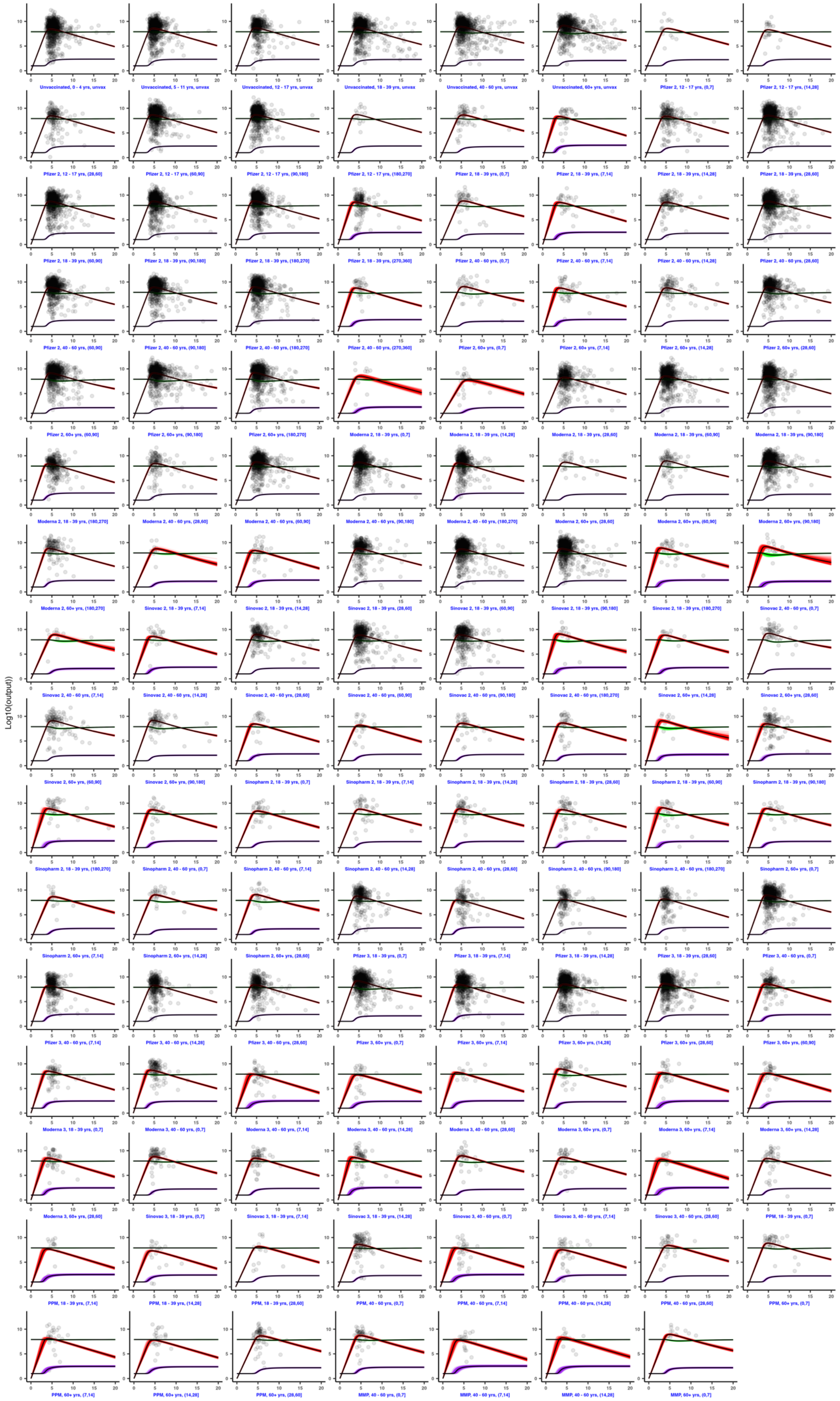
Log10(output))
Unvaccinated, 0 - 4 yrs, unvax
Unvaccinated, 5 - 11 yrs, unvax
Unvaccinated, 12 - 17 yrs, unvax
Unvaccinated, 18 - 39 yrs, unvax
Unvaccinated, 40 - 60 yrs, unvax
Unvaccinated, 60+ yrs, unvax
Pfizer 2, 12 - 17 yrs, (0,7]
Pfizer 2, 12 - 17 yrs, (14,28]
Pfizer 2, 12 - 17 yrs, (28,60]
Pfizer 2, 12 - 17 yrs, (60,90]
Pfizer 2, 12 - 17 yrs, (90,180]
Pfizer 2, 12 - 17 yrs, (180,270]
Pfizer 2, 18 - 39 yrs, (0,7]
Pfizer 2, 18 - 39 yrs, (7,14]
Pfizer 2, 18 - 39 yrs, (14,28]
Pfizer 2, 18 - 39 yrs, (28,60]
Pfizer 2, 18 - 39 yrs, (60,90]
Pfizer 2, 18 - 39 yrs, (90,180]
Pfizer 2, 18 - 39 yrs, (180,270]
Pfizer 2, 18 - 39 yrs, (270,360]
Pfizer 2, 40 - 60 yrs, (0,7]
Pfizer 2, 40 - 60 yrs, (7,14]
Pfizer 2, 40 - 60 yrs, (14,28]
Pfizer 2, 40 - 60 yrs, (28,60]
Pfizer 2, 40 - 60 yrs, (60,90]
Pfizer 2, 40 - 60 yrs, (90,180]
Pfizer 2, 40 - 60 yrs, (180,270]
Pfizer 2, 40 - 60 yrs, (270,360]
Pfizer 2, 60+ yrs, (0,7]
Pfizer 2, 60+ yrs, (7,14]
Pfizer 2, 60+ yrs, (14,28]
Pfizer 2, 60+ yrs, (28,60]
Pfizer 2, 60+ yrs, (60,90]
Pfizer 2, 60+ yrs, (90,180]
Pfizer 2, 60+ yrs, (180,270]
Moderna 2, 18 - 39 yrs, (0,7]
Moderna 2, 18 - 39 yrs, (14,28]
Moderna 2, 18 - 39 yrs, (28,60]
Moderna 2, 18 - 39 yrs, (60,90]
Moderna 2, 18 - 39 yrs, (90,180]
Moderna 2, 18 - 39 yrs, (180,270]
Moderna 2, 40 - 60 yrs, (28,60]
Moderna 2, 40 - 60 yrs, (60,90]
Moderna 2, 40 - 60 yrs, (90,180]
Moderna 2, 40 - 60 yrs, (180,270]
Moderna 2, 60+ yrs, (28,60]
Moderna 2, 60+ yrs, (60,90]
Moderna 2, 60+ yrs, (90,180]
Moderna 2, 60+ yrs, (180,270]
Sinovac 2, 18 - 39 yrs, (7,14]
Sinovac 2, 18 - 39 yrs, (14,28]
Sinovac 2, 18 - 39 yrs, (28,60]
Sinovac 2, 18 - 39 yrs, (60,90]
Sinovac 2, 18 - 39 yrs, (90,180]
Sinovac 2, 18 - 39 yrs, (180,270]
Sinovac 2, 40 - 60 yrs, (0,7]
Sinovac 2, 40 - 60 yrs, (7,14]
Sinovac 2, 40 - 60 yrs, (14,28]
Sinovac 2, 40 - 60 yrs, (28,60]
Sinovac 2, 40 - 60 yrs, (60,90]
Sinovac 2, 40 - 60 yrs, (90,180]
Sinovac 2, 40 - 60 yrs, (180,270]
Sinovac 2, 60+ yrs, (14,28]
Sinovac 2, 60+ yrs, (28,60]
Sinovac 2, 60+ yrs, (60,90]
Sinovac 2, 60+ yrs, (90,180]
Sinopharm 2, 18 - 39 yrs, (0,7]
Sinopharm 2, 18 - 39 yrs, (7,14]
Sinopharm 2, 18 - 39 yrs, (14,28]
Sinopharm 2, 18 - 39 yrs, (28,60]
Sinopharm 2, 18 - 39 yrs, (60,90]
Sinopharm 2, 18 - 39 yrs, (90,180]
Sinopharm 2, 18 - 39 yrs, (180,270]
Sinopharm 2, 40 - 60 yrs, (0,7]
Sinopharm 2, 40 - 60 yrs, (7,14]
Sinopharm 2, 40 - 60 yrs, (14,28]
Sinopharm 2, 40 - 60 yrs, (28,60]
Sinopharm 2, 40 - 60 yrs, (90,180]
Sinopharm 2, 40 - 60 yrs, (180,270]
Sinopharm 2, 60+ yrs, (0,7]
Sinopharm 2, 60+ yrs, (7,14]
Sinopharm 2, 60+ yrs, (14,28]
Sinopharm 2, 60+ yrs, (28,60]
Pfizer 3, 18 - 39 yrs, (0,7]
Pfizer 3, 18 - 39 yrs, (7,14]
Pfizer 3, 18 - 39 yrs, (14,28]
Pfizer 3, 18 - 39 yrs, (28,60]
Pfizer 3, 40 - 60 yrs, (0,7]
Pfizer 3, 40 - 60 yrs, (7,14]
Pfizer 3, 40 - 60 yrs, (14,28]
Pfizer 3, 40 - 60 yrs, (28,60]
Pfizer 3, 60+ yrs, (0,7]
Pfizer 3, 60+ yrs, (7,14]
Pfizer 3, 60+ yrs, (14,28]
Pfizer 3, 60+ yrs, (28,60]
Pfizer 3, 60+ yrs, (60,90]
Moderna 3, 18 - 39 yrs, (0,7]
Moderna 3, 40 - 60 yrs, (0,7]
Moderna 3, 40 - 60 yrs, (7,14]
Moderna 3, 40 - 60 yrs, (14,28]
Moderna 3, 40 - 60 yrs, (28,60]
Moderna 3, 60+ yrs, (0,7]
Moderna 3, 60+ yrs, (7,14]
Moderna 3, 60+ yrs, (14,28]
Moderna 3, 60+ yrs, (28,60]
Sinovac 3, 18 - 39 yrs, (0,7]
Sinovac 3, 18 - 39 yrs, (7,14]
Sinovac 3, 18 - 39 yrs, (14,28]
Sinovac 3, 40 - 60 yrs, (0,7]
Sinovac 3, 40 - 60 yrs, (7,14]
Sinovac 3, 40 - 60 yrs, (28,60]
PPM, 18 - 39 yrs, (0,7]
PPM, 18 - 39 yrs, (7,14]
PPM, 18 - 39 yrs, (14,28]
PPM, 18 - 39 yrs, (28,60]
PPM, 40 - 60 yrs, (0,7]
PPM, 40 - 60 yrs, (7,14]
PPM, 40 - 60 yrs, (14,28]
PPM, 40 - 60 yrs, (28,60]
PPM, 60+ yrs, (0,7]
PPM, 60+ yrs, (7,14]
PPM, 60+ yrs, (14,28]
PPM, 60+ yrs, (28,60]
MMP, 40 - 60 yrs, (0,7]
MMP, 40 - 60 yrs, (7,14]
MMP, 40 - 60 yrs, (14,28]
MMP, 60+ yrs, (0,7]

(b) Omicron

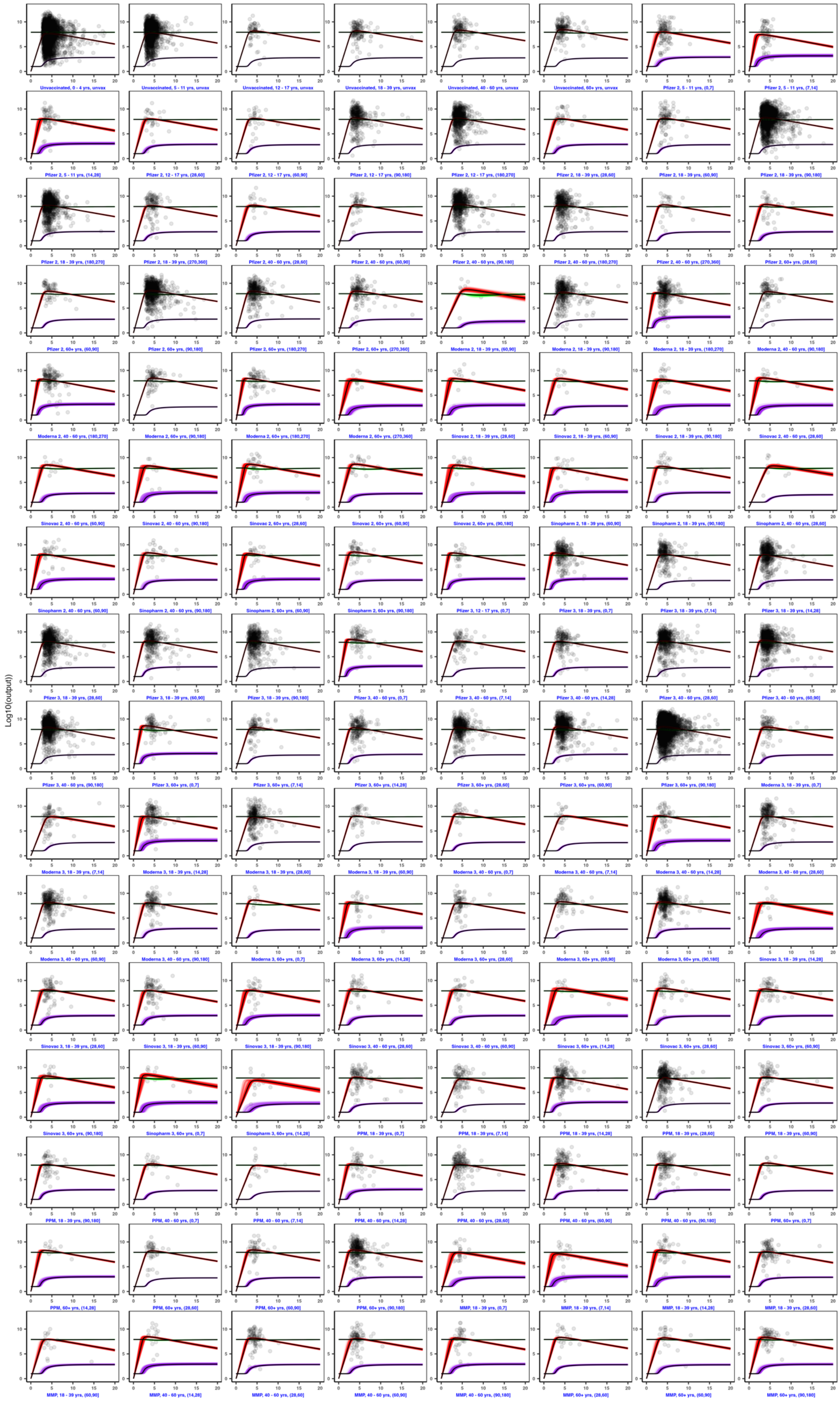

Fig S12. Observed viral load and output from model fit to dataset which only includes patients with one swab for Delta and Omicron infections. Grey dots represent observed viral loads. Black lines are median posterior for virus dynamics. Green lines are samples from the posterior of uninfected target cell dynamics. Purple lines are samples from the posterior of immune response dynamics. Red lines are samples from the posterior of virus dynamics. (a) Delta infections, (b) Omicron infections.

(a) Delta

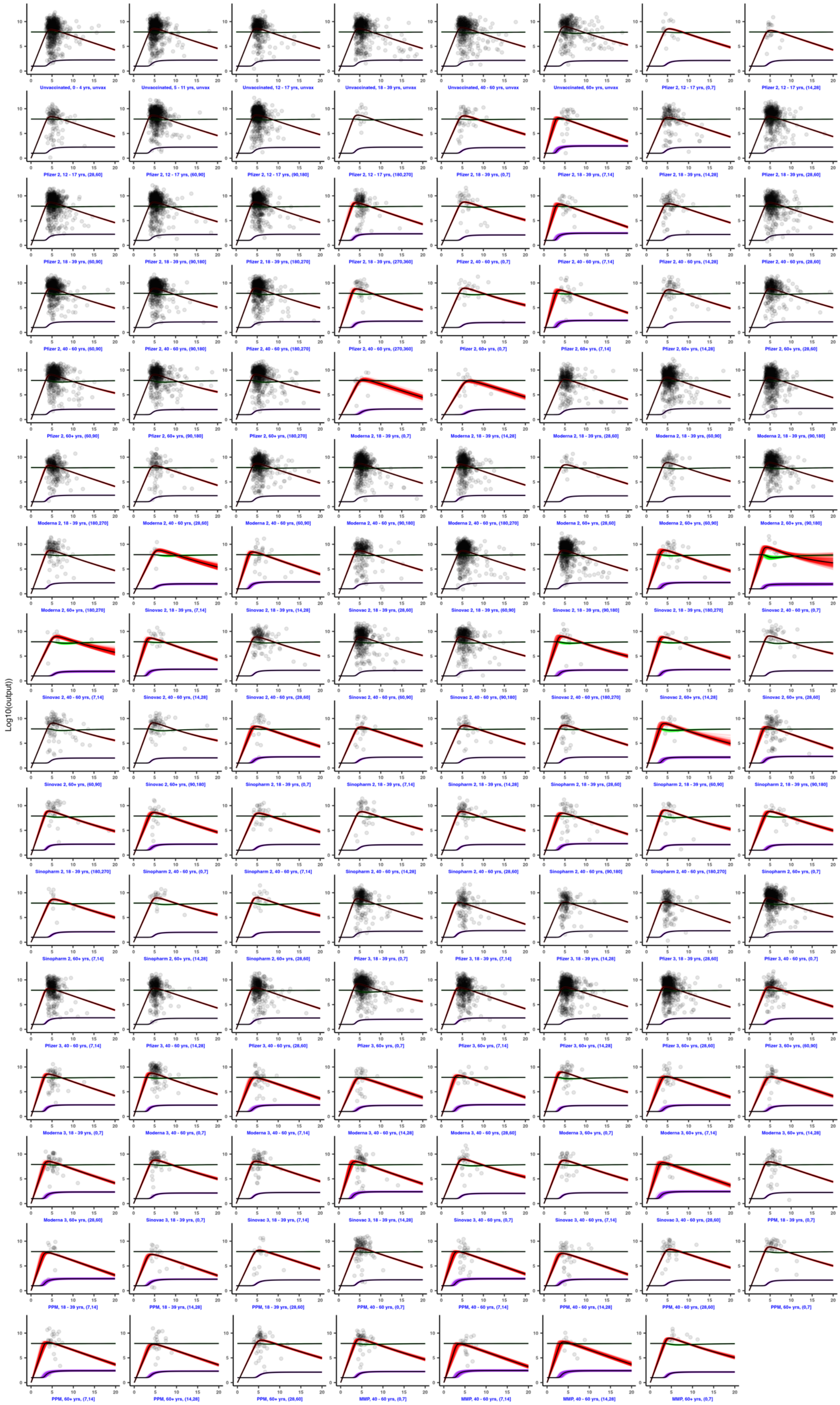
Log10(output))
Unvaccinated, 0 - 4 yrs, unvax
Unvaccinated, 5 - 11 yrs, unvax
Unvaccinated, 12 - 17 yrs, unvax
Unvaccinated, 18 - 39 yrs, unvax
Unvaccinated, 40 - 60 yrs, unvax
Unvaccinated, 60+ yrs, unvax
Pfizer 2, 12 - 17 yrs, (0,7]
Pfizer 2, 12 - 17 yrs, (14,28]
Pfizer 2, 12 - 17 yrs, (28,60]
Pfizer 2, 12 - 17 yrs, (60,90]
Pfizer 2, 12 - 17 yrs, (90,180]
Pfizer 2, 12 - 17 yrs, (180,270]
Pfizer 2, 18 - 39 yrs, (0,7]
Pfizer 2, 18 - 39 yrs, (7,14]
Pfizer 2, 18 - 39 yrs, (14,28]
Pfizer 2, 18 - 39 yrs, (28,60]
Pfizer 2, 18 - 39 yrs, (60,90]
Pfizer 2, 18 - 39 yrs, (90,180]
Pfizer 2, 18 - 39 yrs, (180,270]
Pfizer 2, 18 - 39 yrs, (270,360]
Pfizer 2, 40 - 60 yrs, (0,7]
Pfizer 2, 40 - 60 yrs, (7,14]
Pfizer 2, 40 - 60 yrs, (14,28]
Pfizer 2, 40 - 60 yrs, (28,60]
Pfizer 2, 40 - 60 yrs, (60,90]
Pfizer 2, 40 - 60 yrs, (90,180]
Pfizer 2, 40 - 60 yrs, (180,270]
Pfizer 2, 40 - 60 yrs, (270,360]
Pfizer 2, 60+ yrs, (0,7]
Pfizer 2, 60+ yrs, (7,14]
Pfizer 2, 60+ yrs, (14,28]
Pfizer 2, 60+ yrs, (28,60]
Pfizer 2, 60+ yrs, (60,90]
Pfizer 2, 60+ yrs, (90,180]
Pfizer 2, 60+ yrs, (180,270]
Moderna 2, 18 - 39 yrs, (0,7]
Moderna 2, 18 - 39 yrs, (14,28]
Moderna 2, 18 - 39 yrs, (28,60]
Moderna 2, 18 - 39 yrs, (60,90]
Moderna 2, 18 - 39 yrs, (90,180]
Moderna 2, 18 - 39 yrs, (180,270]
Moderna 2, 40 - 60 yrs, (28,60]
Moderna 2, 40 - 60 yrs, (60,90]
Moderna 2, 40 - 60 yrs, (90,180]
Moderna 2, 40 - 60 yrs, (180,270]
Moderna 2, 60+ yrs, (28,60]
Moderna 2, 60+ yrs, (60,90]
Moderna 2, 60+ yrs, (90,180]
Moderna 2, 60+ yrs, (180,270]
Sinovac 2, 18 - 39 yrs, (7,14]
Sinovac 2, 18 - 39 yrs, (14,28]
Sinovac 2, 18 - 39 yrs, (28,60]
Sinovac 2, 18 - 39 yrs, (60,90]
Sinovac 2, 18 - 39 yrs, (90,180]
Sinovac 2, 18 - 39 yrs, (180,270]
Sinovac 2, 40 - 60 yrs, (0,7]
Sinovac 2, 40 - 60 yrs, (7,14]
Sinovac 2, 40 - 60 yrs, (14,28]
Sinovac 2, 40 - 60 yrs, (28,60]
Sinovac 2, 40 - 60 yrs, (60,90]
Sinovac 2, 40 - 60 yrs, (90,180]
Sinovac 2, 40 - 60 yrs, (180,270]
Sinovac 2, 60+ yrs, (14,28]
Sinovac 2, 60+ yrs, (28,60]
Sinovac 2, 60+ yrs, (60,90]
Sinovac 2, 60+ yrs, (90,180]
Sinopharm 2, 18 - 39 yrs, (0,7]
Sinopharm 2, 18 - 39 yrs, (7,14]
Sinopharm 2, 18 - 39 yrs, (14,28]
Sinopharm 2, 18 - 39 yrs, (28,60]
Sinopharm 2, 18 - 39 yrs, (60,90]
Sinopharm 2, 18 - 39 yrs, (90,180]
Sinopharm 2, 18 - 39 yrs, (180,270]
Sinopharm 2, 40 - 60 yrs, (0,7]
Sinopharm 2, 40 - 60 yrs, (7,14]
Sinopharm 2, 40 - 60 yrs, (14,28]
Sinopharm 2, 40 - 60 yrs, (28,60]
Sinopharm 2, 40 - 60 yrs, (90,180]
Sinopharm 2, 40 - 60 yrs, (180,270]
Sinopharm 2, 60+ yrs, (0,7]
Sinopharm 2, 60+ yrs, (7,14]
Sinopharm 2, 60+ yrs, (14,28]
Sinopharm 2, 60+ yrs, (28,60]
Pfizer 3, 18 - 39 yrs, (0,7]
Pfizer 3, 18 - 39 yrs, (7,14]
Pfizer 3, 18 - 39 yrs, (14,28]
Pfizer 3, 18 - 39 yrs, (28,60]
Pfizer 3, 40 - 60 yrs, (0,7]
Pfizer 3, 40 - 60 yrs, (7,14]
Pfizer 3, 40 - 60 yrs, (14,28]
Pfizer 3, 40 - 60 yrs, (28,60]
Pfizer 3, 60+ yrs, (0,7]
Pfizer 3, 60+ yrs, (7,14]
Pfizer 3, 60+ yrs, (14,28]
Pfizer 3, 60+ yrs, (28,60]
Pfizer 3, 60+ yrs, (60,90]
Moderna 3, 18 - 39 yrs, (0,7]
Moderna 3, 40 - 60 yrs, (0,7]
Moderna 3, 40 - 60 yrs, (7,14]
Moderna 3, 40 - 60 yrs, (14,28]
Moderna 3, 40 - 60 yrs, (28,60]
Moderna 3, 60+ yrs, (0,7]
Moderna 3, 60+ yrs, (7,14]
Moderna 3, 60+ yrs, (14,28]
Moderna 3, 60+ yrs, (28,60]
Sinovac 3, 18 - 39 yrs, (0,7]
Sinovac 3, 18 - 39 yrs, (7,14]
Sinovac 3, 18 - 39 yrs, (14,28]
Sinovac 3, 40 - 60 yrs, (0,7]
Sinovac 3, 40 - 60 yrs, (7,14]
Sinovac 3, 40 - 60 yrs, (28,60]
PPM, 18 - 39 yrs, (0,7]
PPM, 18 - 39 yrs, (7,14]
PPM, 18 - 39 yrs, (14,28]
PPM, 18 - 39 yrs, (28,60]
PPM, 40 - 60 yrs, (0,7]
PPM, 40 - 60 yrs, (7,14]
PPM, 40 - 60 yrs, (14,28]
PPM, 40 - 60 yrs, (28,60]
PPM, 60+ yrs, (0,7]
PPM, 60+ yrs, (7,14]
PPM, 60+ yrs, (14,28]
PPM, 60+ yrs, (28,60]
MMP, 40 - 60 yrs, (0,7]
MMP, 40 - 60 yrs, (7,14]
MMP, 40 - 60 yrs, (14,28]
MMP, 60+ yrs, (0,7]

(b) Omicron

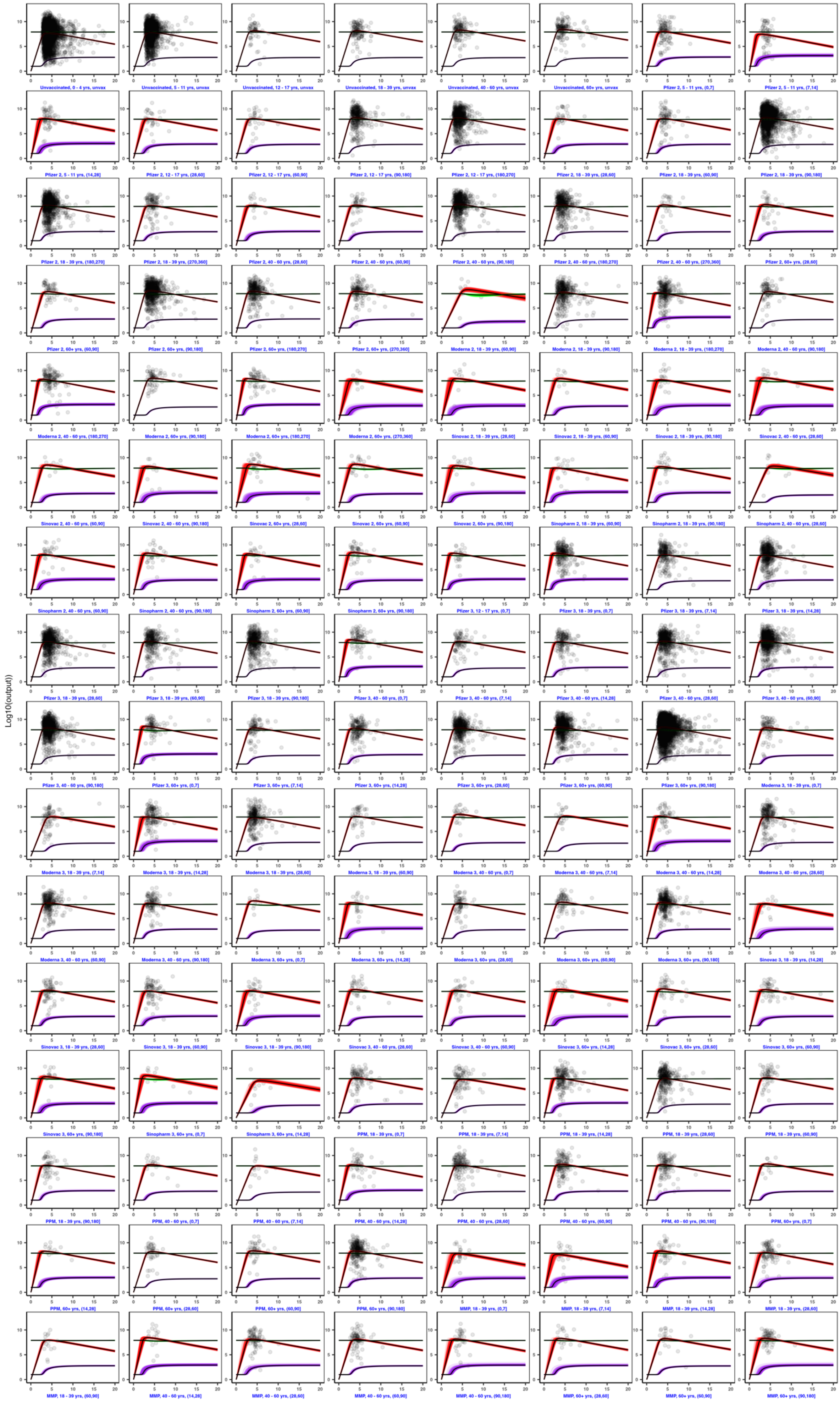

Log10(output1)
Unvaccinated, 0 - 4 yrs, unvax
Unvaccinated, 5 - 11 yrs, unvax
Unvaccinated, 12 - 17 yrs, unvax
Unvaccinated, 18 - 39 yrs, unvax
Unvaccinated, 40 - 60 yrs, unvax
Unvaccinated, 60+ yrs, unvax
Pfizer 2, 5 - 11 yrs, (0,7]
Pfizer 2, 5 - 11 yrs, (7,14]
Pfizer 2, 5 - 11 yrs, (14,28]
Pfizer 2, 12 - 17 yrs, (28,60]
Pfizer 2, 12 - 17 yrs, (60,90]
Pfizer 2, 12 - 17 yrs, (90,180]
Pfizer 2, 12 - 17 yrs, (180,270]
Pfizer 2, 18 - 39 yrs, (28,60]
Pfizer 2, 18 - 39 yrs, (60,90]
Pfizer 2, 18 - 39 yrs, (90,180]
Pfizer 2, 18 - 39 yrs, (180,270]
Pfizer 2, 18 - 39 yrs, (270,360]
Pfizer 2, 40 - 60 yrs, (28,60]
Pfizer 2, 40 - 60 yrs, (60,90]
Pfizer 2, 40 - 60 yrs, (90,180]
Pfizer 2, 40 - 60 yrs, (180,270]
Pfizer 2, 40 - 60 yrs, (270,360]
Pfizer 2, 60+ yrs, (28,60]
Pfizer 2, 60+ yrs, (60,90]
Pfizer 2, 60+ yrs, (90,180]
Pfizer 2, 60+ yrs, (180,270]
Pfizer 2, 60+ yrs, (270,360]
Moderna 2, 18 - 39 yrs, (60,90]
Moderna 2, 18 - 39 yrs, (90,180]
Moderna 2, 18 - 39 yrs, (180,270]
Moderna 2, 40 - 60 yrs, (90,180]
Moderna 2, 40 - 60 yrs, (180,270]
Moderna 2, 60+ yrs, (90,180]
Moderna 2, 60+ yrs, (180,270]
Moderna 2, 60+ yrs, (270,360]
Sinovac 2, 18 - 39 yrs, (28,60]
Sinovac 2, 18 - 39 yrs, (60,90]
Sinovac 2, 18 - 39 yrs, (90,180]
Sinovac 2, 40 - 60 yrs, (28,60]
Sinovac 2, 40 - 60 yrs, (60,90]
Sinovac 2, 40 - 60 yrs, (90,180]
Sinovac 2, 60+ yrs, (28,60]
Sinovac 2, 60+ yrs, (60,90]
Sinovac 2, 60+ yrs, (90,180]
Sinopharm 2, 18 - 39 yrs, (60,90]
Sinopharm 2, 18 - 39 yrs, (90,180]
Sinopharm 2, 40 - 60 yrs, (28,60]
Sinopharm 2, 40 - 60 yrs, (60,90]
Sinopharm 2, 40 - 60 yrs, (90,180]
Sinopharm 2, 60+ yrs, (60,90]
Sinopharm 2, 60+ yrs, (90,180]
Pfizer 3, 12 - 17 yrs, (0,7]
Pfizer 3, 18 - 39 yrs, (0,7]
Pfizer 3, 18 - 39 yrs, (7,14]
Pfizer 3, 18 - 39 yrs, (14,28]
Pfizer 3, 18 - 39 yrs, (28,60]
Pfizer 3, 18 - 39 yrs, (60,90]
Pfizer 3, 18 - 39 yrs, (90,180]
Pfizer 3, 40 - 60 yrs, (0,7]
Pfizer 3, 40 - 60 yrs, (7,14]
Pfizer 3, 40 - 60 yrs, (14,28]
Pfizer 3, 40 - 60 yrs, (28,60]
Pfizer 3, 40 - 60 yrs, (60,90]
Pfizer 3, 40 - 60 yrs, (90,180]
Pfizer 3, 60+ yrs, (0,7]
Pfizer 3, 60+ yrs, (7,14]
Pfizer 3, 60+ yrs, (14,28]
Pfizer 3, 60+ yrs, (28,60]
Pfizer 3, 60+ yrs, (60,90]
Pfizer 3, 60+ yrs, (90,180]
Moderna 3, 18 - 39 yrs, (0,7]
Moderna 3, 18 - 39 yrs, (7,14]
Moderna 3, 18 - 39 yrs, (14,28]
Moderna 3, 18 - 39 yrs, (28,60]
Moderna 3, 18 - 39 yrs, (60,90]
Moderna 3, 40 - 60 yrs, (0,7]
Moderna 3, 40 - 60 yrs, (7,14]
Moderna 3, 40 - 60 yrs, (14,28]
Moderna 3, 40 - 60 yrs, (28,60]
Moderna 3, 40 - 60 yrs, (60,90]
Moderna 3, 40 - 60 yrs, (90,180]
Moderna 3, 60+ yrs, (0,7]
Moderna 3, 60+ yrs, (14,28]
Moderna 3, 60+ yrs, (28,60]
Moderna 3, 60+ yrs, (60,90]
Moderna 3, 60+ yrs, (90,180]
Sinovac 3, 18 - 39 yrs, (14,28]
Sinovac 3, 18 - 39 yrs, (28,60]
Sinovac 3, 18 - 39 yrs, (60,90]
Sinovac 3, 18 - 39 yrs, (90,180]
Sinovac 3, 40 - 60 yrs, (28,60]
Sinovac 3, 40 - 60 yrs, (60,90]
Sinovac 3, 60+ yrs, (14,28]
Sinovac 3, 60+ yrs, (28,60]
Sinovac 3, 60+ yrs, (60,90]
Sinovac 3, 60+ yrs, (90,180]
Sinopharm 3, 60+ yrs, (0,7]
Sinopharm 3, 60+ yrs, (14,28]
PPM, 18 - 39 yrs, (0,7]
PPM, 18 - 39 yrs, (7,14]
PPM, 18 - 39 yrs, (14,28]
PPM, 18 - 39 yrs, (28,60]
PPM, 18 - 39 yrs, (60,90]
PPM, 18 - 39 yrs, (90,180]
PPM, 40 - 60 yrs, (0,7]
PPM, 40 - 60 yrs, (7,14]
PPM, 40 - 60 yrs, (14,28]
PPM, 40 - 60 yrs, (28,60]
PPM, 40 - 60 yrs, (60,90]
PPM, 40 - 60 yrs, (90,180]
PPM, 60+ yrs, (0,7]
PPM, 60+ yrs, (14,28]
PPM, 60+ yrs, (28,60]
PPM, 60+ yrs, (60,90]
PPM, 60+ yrs, (90,180]
MMP, 18 - 39 yrs, (0,7]
MMP, 18 - 39 yrs, (7,14]
MMP, 18 - 39 yrs, (14,28]
MMP, 18 - 39 yrs, (28,60]
MMP, 18 - 39 yrs, (60,90]
MMP, 40 - 60 yrs, (14,28]
MMP, 40 - 60 yrs, (28,60]
MMP, 40 - 60 yrs, (60,90]
MMP, 40 - 60 yrs, (90,180]
MMP, 60+ yrs, (28,60]
MMP, 60+ yrs, (60,90]
MMP, 60+ yrs, (90,180]

Fig S13. **Posterior distributions of estimated parameter values in Model Type 2 with Delta dataset, for a range of standard deviations for normally distributed errors of log-viraemia measurements.** For all plots, red line and shaded region represents the posterior distribution for $\sigma^2 = 0.5$, green line and shaded region represents the posterior distribution of the original fit in which $\sigma^2 = 1$, blue line and shaded region represents the posterior distribution for $\sigma^2 = 2$ (a) Posterior distributions for infection rate of target cells (β) (b) Posterior distributions for natural clearance of virus (c) (c) Posterior distributions for estimated age modifier values ($\theta_{2\text{-}6}$) (d) Posterior distributions for infected cell clearance by immunity (γ) (e) Posterior distributions for growth rate of immunity (ω)

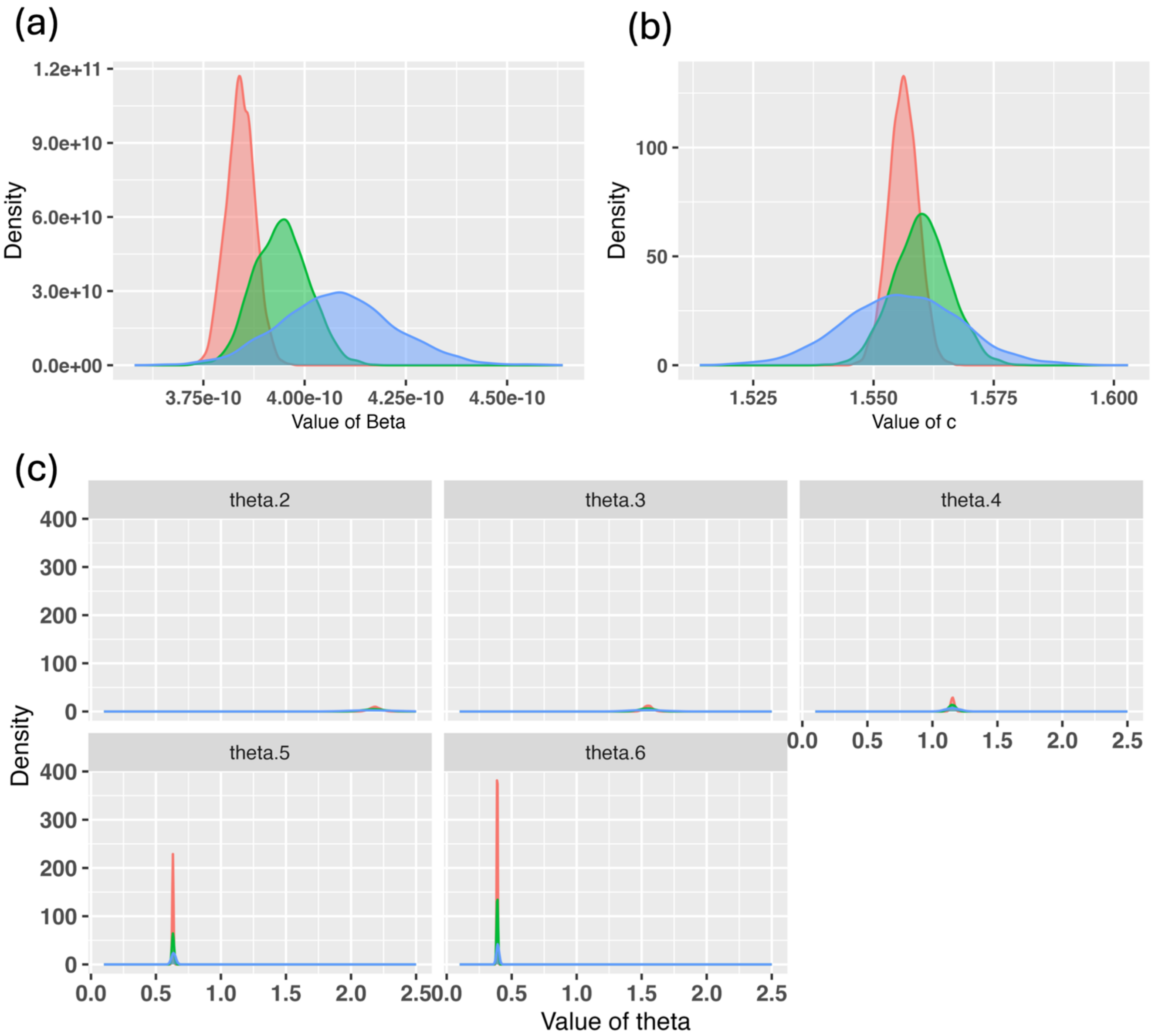

(d)

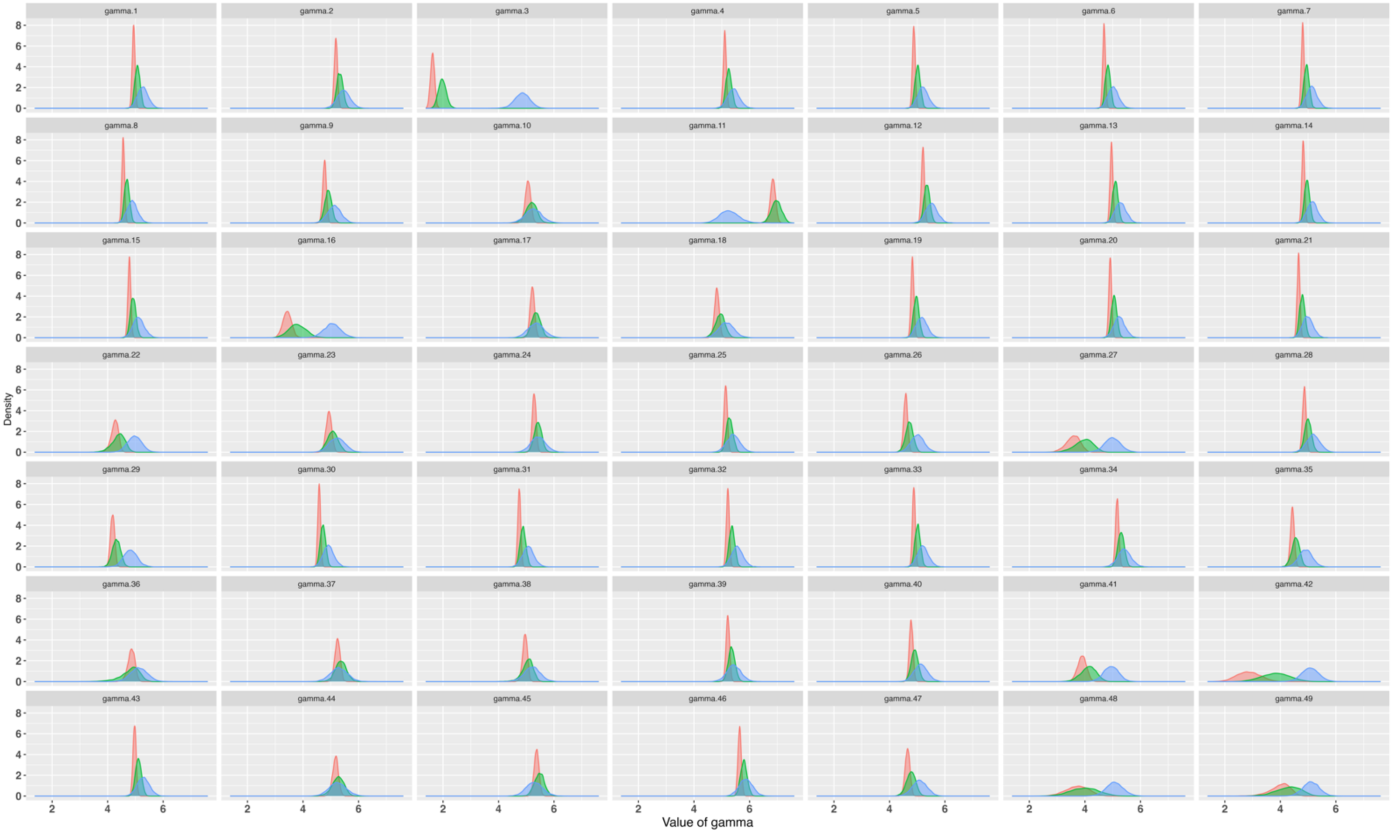
gamma.1
gamma.2
gamma.3
gamma.4
gamma.5
gamma.6
gamma.7
gamma.8
gamma.9
gamma.10
gamma.11
gamma.12
gamma.13
gamma.14
gamma.15
gamma.16
gamma.17
gamma.18
gamma.19
gamma.20
gamma.21
gamma.22
gamma.23
gamma.24
gamma.25
gamma.26
gamma.27
gamma.28
gamma.29
gamma.30
gamma.31
gamma.32
gamma.33
gamma.34
gamma.35
gamma.36
gamma.37
gamma.38
gamma.39
gamma.40
gamma.41
gamma.42
gamma.43
gamma.44
gamma.45
gamma.46
gamma.47
gamma.48
gamma.49
Density
Value of gamma

(e)

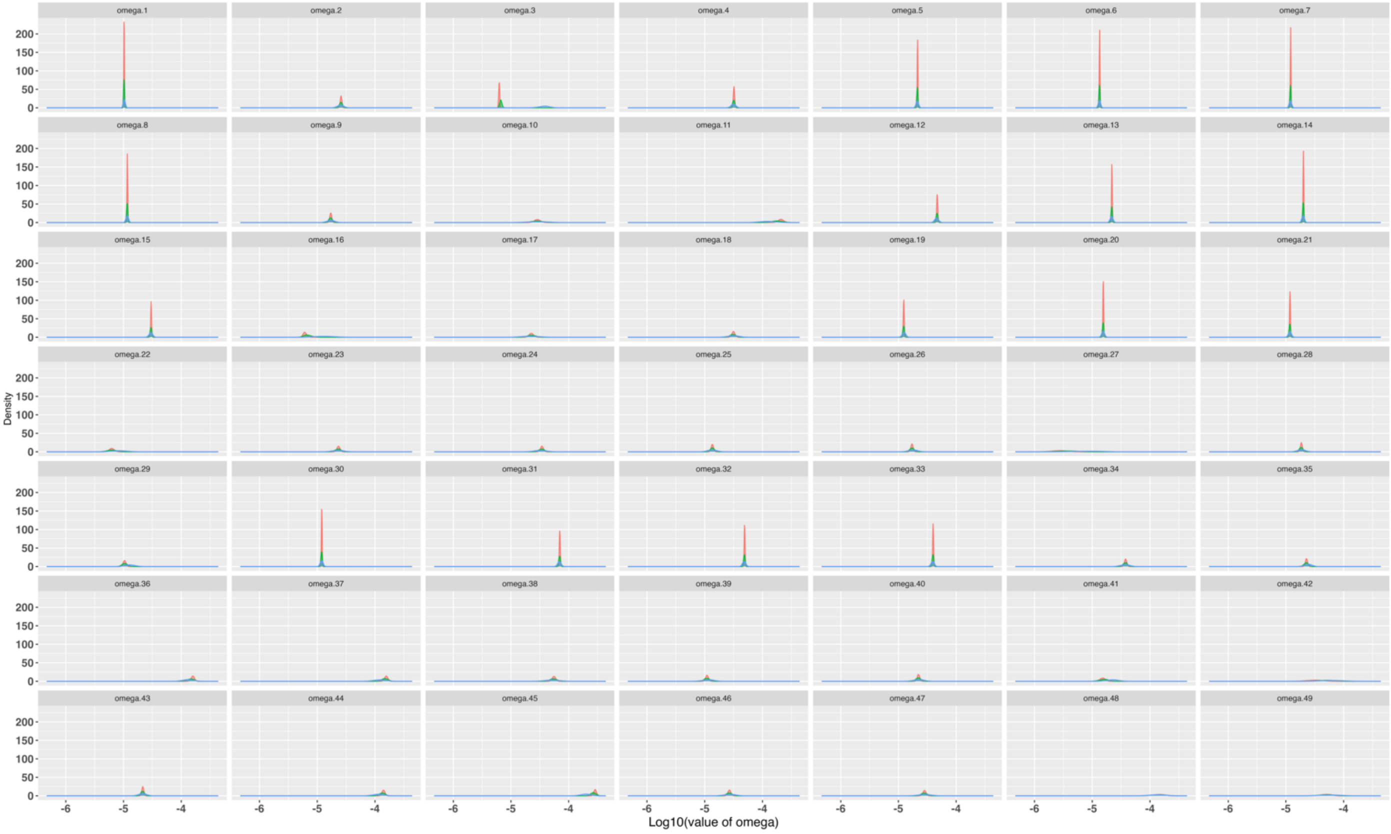
omega.1
omega.2
omega.3
omega.4
omega.5
omega.6
omega.7
omega.8
omega.9
omega.10
omega.11
omega.12
omega.13
omega.14
omega.15
omega.16
omega.17
omega.18
omega.19
omega.20
omega.21
omega.22
omega.23
omega.24
omega.25
omega.26
omega.27
omega.28
omega.29
omega.30
omega.31
omega.32
omega.33
omega.34
omega.35
omega.36
omega.37
omega.38
omega.39
omega.40
omega.41
omega.42
omega.43
omega.44
omega.45
omega.46
omega.47
omega.48
omega.49
Density
Log10(value of omega)

Fig S14. **Posterior distributions of estimated parameter values in Model Type 2 with Omicron dataset, for a range of standard deviations for normally distributed errors of log-viraemia measurements.** For all plots, red line and shaded region represents the posterior distribution for $\sigma^2 = 0.5$, green line and shaded region represents the posterior distribution of the original fit in which $\sigma^2 = 1$, blue line and shaded region represents the posterior distribution for $\sigma^2 = 2$ (a) Posterior distributions for infection rate of target cells (β) (b) Posterior distributions for natural clearance of virus (c) (c) Posterior distributions for estimated age modifier values ($\theta_{2\text{-}6}$) (d) Posterior distributions for infected cell clearance by immunity (γ) (e) Posterior distributions for growth rate of immunity (ω)

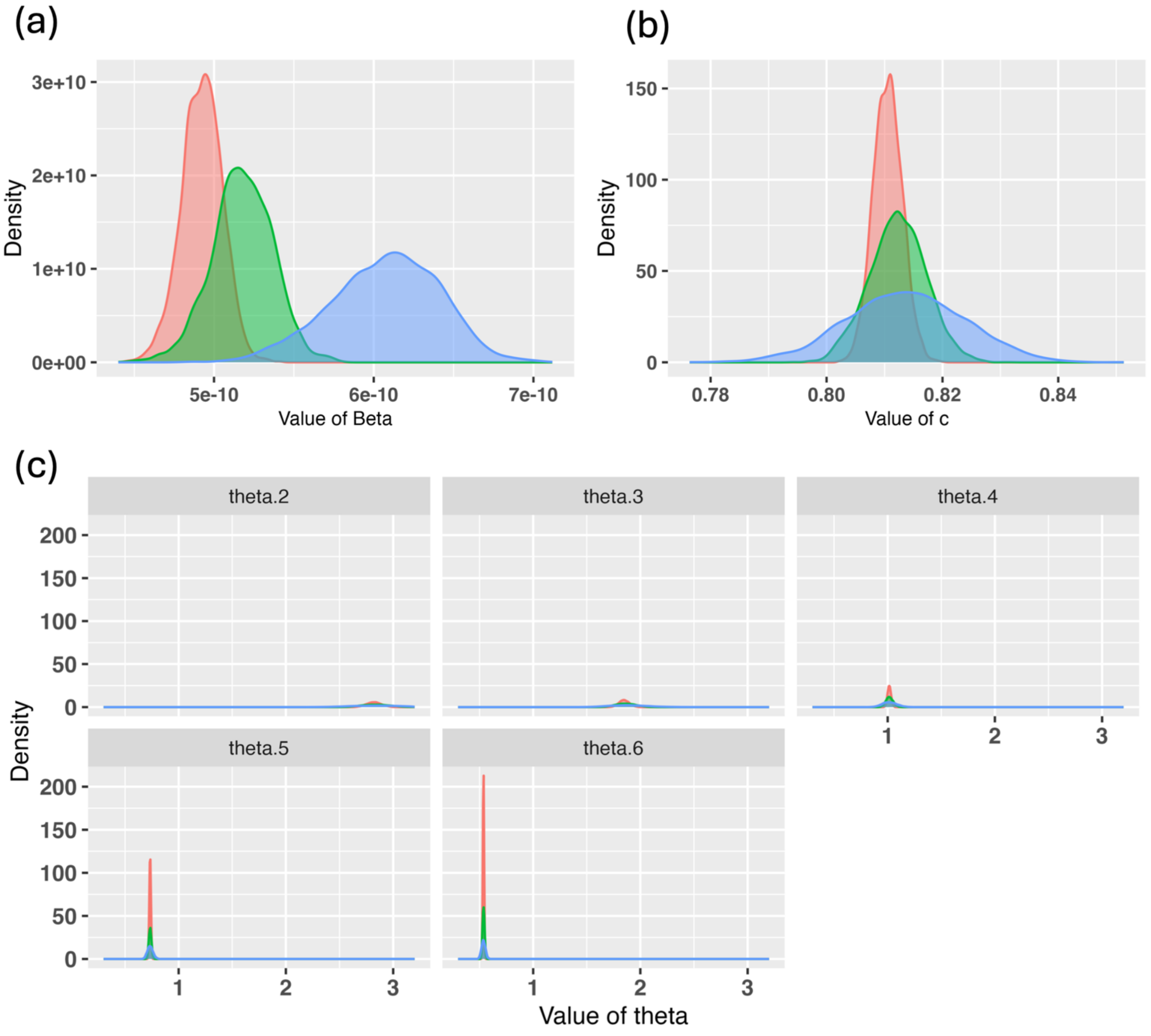

(d)

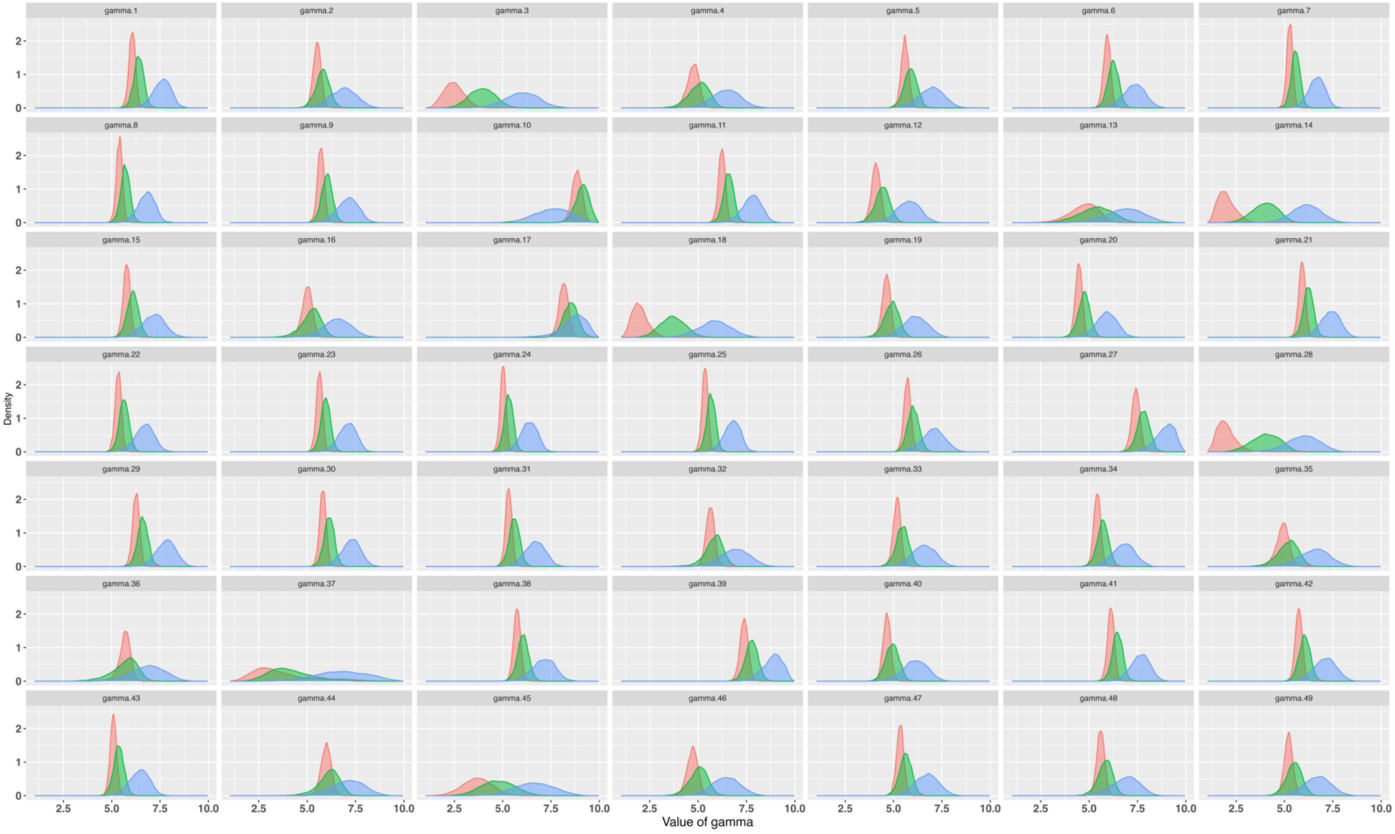

(e)

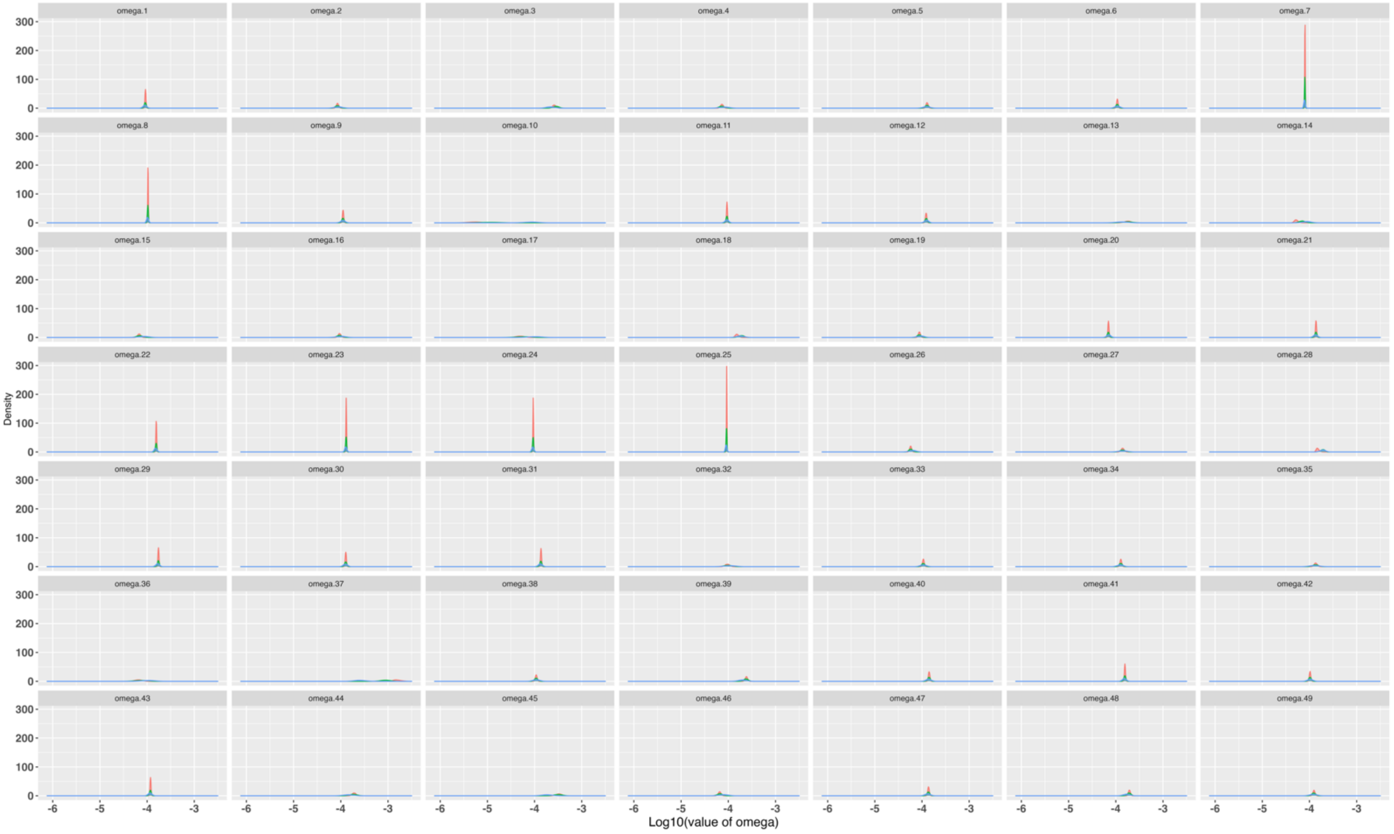

Supplement: Supplementary file 2 — Supplementary Material 2 [file 12879_2024_9572_MOESM2_ESM.pdf]
